# Genetic features of SARS-CoV-2 Alpha, Delta, and Omicron variants and their association with the clinical severity of COVID-19 in Vietnam

**DOI:** 10.1016/j.ijregi.2024.03.003

**Published:** 2024-03-13

**Authors:** Le Van Nam, Trinh Cong Dien, Le Van Nguyen Bang, Pham Ngoc Thach, Le Van Duyet

**Affiliations:** 1 Departments of Infectious Disease, Military Hospital, Hanoi, Vietnam; 2 Luong The Vinh High School, Hanoi, Vietnam; 3 Micobiology and Molecular Biology Department, National Hospital for Tropical Diseases, Hanoi, Vietnam

**Keywords:** SARS-COV-2, COVID-19, Alpha variant, Delta variant, Omicron variant, Disease severity

## Abstract

**Objectives:**

We investigated the genetic variations in the Alpha, Delta, and Omicron variants of SARS-CoV-2 and their association with clinical status and treatment outcomes in patients with COVID-19.

**Methods:**

MiSeq was used to sequence the Alpha, Delta, and Omicron genomes, and MEGA 6.6 was used to define the nucleotide variations. We determined the association between clinical severity and treatment outcomes for the SARS-CoV-2 variants.

**Results:**

The BA.1.1 and BA.2 lineages of the Omicron variant had 57-59 mutations, which is 2-2.7-fold higher than that of the B.1.1.7 (Alpha), B.1.617.2, and AY.57 (Delta) lineages. We found distinct mutations in SARS-CoV-2: five in Alpha (C26305T, G26558T, G7042T, C14120T, and C27509T); seven in Delta (C26408T, C1403T, C5184T, C9891T, T11418C, C11514T, and C22227T); and three in Omicron (C26408T, C8991T, and C25810T). Patients with the Delta variant had a severe rate of 23.8%, a critical rate of 53.7%, and a mortality rate of 38.9%, which were significantly higher than those with the Omicron and Alpha variants.

**Conclusions:**

The Alpha, Delta, and Omicron variants in this study had genetic diversity and differed from the strains reported in other countries, with the Delta variant producing significantly more clinical severity and mortality than the Alpha and Omicron variants.

## Highlights

• The Alpha, Delta, and Omicron variants exhibited alterations that varied among the variants worldwide.
• Severe, critical, and fatal cases were observed only for the Delta and Omicron variants.
• A total of 57 had significantly higher rates of severe, critical, and fatal cases than B.1.617.2, BA.1.1, and BA.2.
• Older age and Delta infection were independent predictors of mortality in patients with COVID-19.

## Introduction

SARS-CoV-2 is a coronavirus that causes severe acute respiratory syndrome. It was detected for the first time in December 2019 in Wuhan, China and was given formal designation by the International Committee for the Classification of Viruses in February 2020 [1]. SARS-CoV-2 caused the COVID-19 pandemic and, as of February 3, 2023, >754 million infections and >6 million deaths were reported [2]. Vietnam had four outbreaks since the first case was reported on January 23, 2020, and the emergence of novel variants has negatively impacted daily life [3].

Patients with SARS-CoV-2 infection can present with a range of clinical presentations, from no symptoms to moderate viral pneumonia and severe respiratory failure, multiorgan system dysfunction, sepsis, and death. In total, 40-45% of the patients who tested positive for the virus were asymptomatic; many remained asymptomatic after virus clearance from the upper respiratory tract and could infect others [4]. Other patients developed symptoms between 2 and 14 days after exposure, with a mean incubation period of approximately 5 days [5]. Disease symptoms varied by region, ethnicity, and nation, particularly, in the elderly and those with chronic conditions, immunodeficiency, or cancer [6].

The SARS-CoV-2 single-stranded RNA virus has a genome size of 26-32 kb, making it the RNA virus with the largest genome [7]. RNA viruses have a faster rate of mutation than DNA viruses, leading to a wide range of mutations across the entire genome [8]. In patients with COVID-19, cumulative alterations between the viral genomes of the parent and its progeny may contribute to the differences in symptoms and clinical course with each viral replication cycle at different time points [9]. There have been statistical reports on new variants, with the hypothesis of increased contagiousness, disease severity, proportion of patients requiring hospitalization for medical care, decreased antibody response ability, and antibody response, as well as preventive vaccines, posing challenges in disease control and vaccine production [10]. Although preliminary studies on SARS-CoV-2 genome sequencing have been performed in Vietnam, few have analyzed the correlation between clinical status and treatment outcomes in patients infected with SARS-CoV-2 variants.

The National Hospital for Tropical Diseases (NHTD) is a central hospital in northern Vietnam that admits and treats patients with COVID-19, particularly, those with severe and critical conditions. Identifying sequence variants of SARS-CoV-2 that produce COVID-19 is important to assess the course of preventive action, diagnosis, prognosis, and therapy. We conducted this study to assess the genetic mutation level of SARS-CoV-2 variants and their association with clinical status and treatment outcomes in patients with COVID-19.

## Material and methods

### Study population

A total of 240 patients with COVID-19 were hospitalized at the NHTD. The patients were clinically assessed and classified, and SARS-CoV-2 genome sequencing was performed. Nasopharyngeal swab samples were obtained upon hospitalization for real-time reverse transcription-polymerase chain reaction (RT-PCR) testing and genome sequencing. Clinical symptoms, regular laboratory testing, and treatment regimen and outcome were collected during the hospital stay.

### Genomic RNA isolation

Viral RNA was isolated directly from throat swabs of patients with COVID-19 using Qiagen kits (QIAamp Viral RNA Mini Kit, Qiagen Sciences, Germantown, MD, USA) in accordance with the manufacturer's protocol. In brief, 140-µl throat swabs were suspended in 560 µl of lysis buffer (buffer AVL) containing carrier RNA, incubated at room temperature (15-25°C) for 10 minutes, mixed with 560 µl ethanol (96-100%), and treated in accordance with the manufacturer's protocol (for microfuge-scale preparations).

### Real-time reverse transcription-polymerase chain reaction (RT-PCR) of SARS-CoV-2

Throat swabs from patients were used for RNA isolation and detection of SARS-CoV-2 with *E* and *RdRp*, using the World Health Organization–recommended Berlin real-time RT-PCR method [11]. For real-time RT-PCR, we used SuperScript III One-Step RT-PCR System with the Platinum Taq DNA Polymerase Kit (Invitrogen, Carlsbad, CA, USA) and two specific primer/probe combinations: E Sarbeco F1/E Sarbeco R2/E Sarbeco P1 (for *E*) and RdRP SARSr-F2/RdRP SARSr-R1/RdRP S (for *RdRp*) (Supplementary Table 1). Thermal cycling involved complementary DNA synthesis cycle at 55°C for 10 minutes, followed by denaturation at 95°C for 3 minutes, then 45 cycles of denaturation at 95°C for 15 seconds, primer/probe binding to the DNA template, and fluorescence collection at 58°C for 30 seconds.

### Whole genome sequencing

The MiSeq sequencer and illuminating reagents were used in this study. SARS-CoV-2 RNA was used as a template to generate complementary DNA, which was cloned using 14 specific primer pairs (Supplementary Table 2). The postpolymerase chain reaction DNA product was confirmed on a 1.5% agarose gel in 1 × Tris-acetate-ethylenediamine tetraacetic acid buffer. The polymerase chain reaction products were measured and normalized for concentration before being cut into small DNA fragments. The small DNA products were attached to Index 1 and Index 2, as specified by the manufacturer. Excess products were purified, and the samples were standardized and quantified. When the library preparation stage was complete, the samples were loaded into the MiSeq machine for sequencing. Concatenated and low-quality sequences were removed from the original data. The viral genome sequence was constructed using fragments read by the CLC software and a reference sequence—SARS-CoV-2 strain from Wuhan, China in 2019 (accession number: NC_045512). The coverage of each nucleotide site in the gene sequence was calculated using SAMtools.

### Clinical data collection and mutation analysis

We collected patient data, including clinical and treatment outcomes, during treatment at the NHTD. The laboratory data collected included SARS-CoV-2 genome sequences, which were systematically documented.

The nucleotide sequences of the 240 SARS-CoV-2 samples were entered into the SARS-CoV-2 database (https://www.gisaid.org/collaborations/enabled-by-hcov-19-data-from-gisaid/) to define variants. Changes in nucleotide sequences of the 240 SARS-CoV-2 samples that circulated in Vietnam were compared with SARS-CoV-2 strains circulating in China, Ghana, the United Kingdom, the United States, and Australia based on *E, M, N, ORF1a, ORF1b, ORF3a, ORF6, ORF7a, ORF7b, ORF8, ORF9b*, and *S* sequences (Global Initiative of Sharing All Influenza Data numbers of these strains are listed in Supplementary Data). MEGA was used to analyze genomic variations in SARS-CoV-2 (version 6.06).

### Phylogenetic analysis

A phylogenetic tree was constructed from the 240 genome sequences of pathogenic SARS-CoV-2 strains, SARS-CoV-2 strains from around the world, and the reference SARS-CoV-2 Wuhan-2019 strain (GenBank number: NC_045512) using Nextclade online software (https://clades.nextstrain.org). Nextclade performs the steps, such as translation, sequence comparison, clade assignment, and phylogenetic sequencing, of genome sequences using the Smith–Waterman algorithm for pair-wise sequence alignment. The resulting phylogenetic tree is visualized using Nextstrain Auspice; each subtree is shown for visualization to find evidence for local and global transmissions.

### Statistical analysis

All data were collected, processed, and analyzed using SPSS 20.0 and Stata 10.0 software. Qualitative variables are presented as frequencies and percentages. Percentages between ≥2 groups were compared using the chi-square or Fisher's exact test; the mean of two independent groups was compared using Student's *t*-test for normally distributed variables and the Mann–Whitney *U* test for non-normally distributed variables. We conducted a univariate analysis to evaluate mortality and survival with age, gender, underlying medical condition, vaccination status, and variations. Then, multivariable logistic regression was used to assess the independence of risk variables for mortality in patients with COVID-19. The patient's death was judged to be related to COVID-19; the other reasons are just risk factors that have been analyzed.

## Results

### Characteristics of patients hospitalized with COVID-19

Of the 240 patients with COVID-19, women (51.7%) outnumbered men (48.3%). The mean age was 46.23 years; the oldest patient was 95 years old and the youngest was 1 year old. The clinical status of patients was mild (38.8%), moderate (25.4%), severe (15.4%), and critical (20.4%). SARS-CoV-2 variants were classified into three groups: 20.0% Alpha (lineage B.1.1.7), 40% Delta (17.5% for lineage B.1.617.2 and 22.5% for lineage AY.57), and 40.1% Omicron (3.8% for BA.1.1 and 36.3% for BA.2). The vaccination status of the 240 patients were as follows: 62.1% unvaccinated, 8.3% received one dose, 17.5% received two doses, and 12.1% received three doses. The average treatment duration was >7 days (up to >95%), whereas >14 days only represented 45%. The treatment outcomes included 65.0% of the patients being cured, 22.1% experiencing symptom improvement, and 12.9% dying (Table 1).

**Table 1:** General characteristics, clinical features, and treatment features of patients with COVID-19.

Characteristics	n (%)
**Gender**	
Male	116 (48.3)
Female	124 (51.7)
**Age (years)**	
Max	95
Min	1
Mean ± SD	46.23 ± 24.20
**Clinical status**	
Mild	93 (38.8)
Moderate	61 (25.4)
Severe	37 (15.4)
Critical	49 (20.4)
**SARS-CoV-2 variants**	
B.1.1.7 (Alpha)	48 (20.0)
B.1.617.2 (Delta)	42 (17.5)
AY.57 (Delta)	54 (22.5)
BA.1.1 (Omicron)	9 (3.8)
BA.2 (Omicron)	87 (36.3)
**COVID-19 vaccine**	
None	149 (62.1)
1 dose	20 (8.3)
2 doses	42 (17.5)
3 doses	29 (12.1)
**Treatment duration**	
<7 days	11 (4.6)
7-14 days	121 (50.4)
>14 days	108 (45.0)
**Treatment outcomes**	
Cure	156 (65.0)
Symptom relief	52 (22.1)
Death	31 (12.9)

### Genetic features of patients hospitalized with COVID-19

The Alpha variant (lineage B.1.1.7) has 31 mutations: 10 in *S*; eight in *ORF1a*; four in *N* and *ORF8*; two in *ORF1b*; one each in *E, M*, and *ORF7a*; and zero in *ORF3a, ORF6, ORF7b*, and *ORF9b*. In the Delta variant, lineages B.1.617.2 and AY.57 had 22 and 29 mutations, respectively. The number of mutations and mutation types in *ORF1a, ORF1b, E*, and *S* varied. The Omicron variant had the highest mutation number in lineages BA.1.1 (n = 57) and BA.2 (n = 59), and *ORF1a* and *ORF3a* had variable mutation numbers. The Omicron variant had three nonmutant genes in lineages BA.1.1 and BA.2: *ORF7a, ORF7b*, and *ORF8* (Table 2).

**Table 2:** Genome mutations in SARS-CoV-2 variants Alpha, Delta, and Omicron.

Genes	Alpha	Delta		Omicron	
	B.1.1.7	B.1.617.2	AY.57	BA.1.1	BA.2
***E***	1	0	1	2	2
***M***	1	1	1	2	2
***N***	4	3	3	6	6
***ORF1a***	8	1	5	11	12
***ORF1b***	2	2	3	4	4
***ORF3a***	0	1	1	1	2
***ORF6***	0	0	0	1	1
***ORF7a***	1	1	1	0	0
***ORF7b***	0	0	0	0	0
***ORF8***	4	2	2	0	0
***ORF9b***	0	1	1	4	4
***S***	10	10	11	26	26
**Total**	**31**	**22**	**29**	**57**	**59**

Our data demonstrated that patients with the Alpha variant (B.1.1.7) have a younger median age than those with the Delta (B.1.617.2, AY.57) and Omicron (BA.1.1, BA.2) variants. Patients with the Delta and Omicron variants were older than those with the Alpha variant. However, the statistical analysis revealed that age differences between patents with Alpha, Delta, and Omicron variants were not statistically significant (*P* >0.05) (Supplement Table 1). Furthermore, differences between patients with comorbidities, such as diabetes, cardiovascular disease, cancer, pneumonia, stroke, cancer, and kidney disease, and Alpha, Delta, and Omicron variants were statistically insignificant (Supplement Table 1).

A comparison of genomic sequences of the 240 SARS-CoV-2 Alpha, Delta, and Omicron variants with those of SARS-CoV-2 Alpha, Delta, and Omicron variants on other continents revealed significant differences. In this study, we found five new mutations in the Alpha variant: C26305T (*E*), G26558T (*M*), G7042T (*ORF1a*), C14120T (*ORF1b*), and C27509T (*ORF7*a). These alterations were 100% different from the Alpha variant of SARS-CoV-2 that circulated in China and the United States, 80% different from those in Ghana and Australia, and 60% different from those in the UK during January 2021 to April 2021 (Table 3). Similarly, the Delta variant comprised mutations in two genes: *ORF1a* (C1403T, C5184T, C9891T, T11418C, and C11514T) and *S* (C22227T), which are very similar to the Delta variant in China and the United States but differ from those in Ghana, the United Kingdom, and Australia. In this study, the mutation in *E* (C26408T) was only detected in SARS-CoV-2 (Table 4). In the Omicron variant, three mutations were found: in *E* (C26408T), *ORF1*a (C8991T), and *ORF3*a (C25810T), which were not found in several SARS-CoV-2 strains from the five countries studied (Table 3).

**Table 3:** Differences in mutations in SARS-CoV-2 in this study and circulating SARS-CoV-2 strains in other countries.

Variants	Genes	Mutations in this study	China (23)^a^	Ghana (251)^a^	UK (630)^a^	USA (679)^a^	Australia (514)^a^
**Alpha**	*E*	C26305**T** (L21F)	C	C	C	C	C
	*M*	G26558**T** (E12D)	G	G	G	G	G
	*ORF1a*	G7042**T** (M2259I)	G	G	**T**	G	G
	*ORF1b*	C14120**T** (P218L)	C	T	**T**	C	**T**
	*ORF7a*	C27509**T** (T39I)	C	C	C	C	C

			**China** (326)^a^	**Ghana** (188)^a^	**UK** (571)^a^	**USA** (497)^a^	**Australia** (259)^a^

**Delta**	*E*	C26408**T** (S55F)	C	C	C	C	C
	*ORF1a*	C1403**T** (P380S)	**T**	C	C	**T**	C
		C5184**T** (P1640L)	**T**	C	C	**T**	C
		C9891**T** (A3209V)	**T**	C	C	**T**	C
		T11418**C** (V3718A)	**C**	T	T	**C**	T
		C11514**T** (T3750I)	**T**	C	C	**T**	C
	*S*	C22227**T** (A222V)	**T**	C	C	**T**	C

			**China** (567)^a^	**Ghana** (41)^a^	**UK** (532)^a^	**USA** (595)^a^	**Australia** (518)^a^

**Omicron**	*E*	C26408**T** (S55F)	C	C	C	C	C
	*ORF1a*	C8991**T** (A2909V)	C	C	C	C	C
	*ORF3a*	C25810**T** (L140F)	C	C	C	C	C

a Numbers of SARS-CoV-2 strains with GISAID numbers (see the GISAID numbers in Supplementary Data). Abbreviations: GISAID, Global Initiative of Sharing All Influenza Data.

**Table 4:** Correlation of SARS-CoV-2 variants with vaccination, clinical severity, treatment duration, and outcome.

Variants	B.1.1.7 n = 48	B.1.617.2 n = 42	AY.57 n = 54	BA.1.1 n = 9	BA.2 n = 87	*P*
**Vaccination, n (%)**						
None	48 (100.0)	24 (57.1)	27 (50.0)	3 (33.3)	47 (54.0)	**<0.001**
1 dose	0 (0.0)	9 (21.4)	8 (14.8)	0 (0.0)	3 (3.4)	**0.001**
2 doses	0 (0.0)	9 (21.4)	18 (33.3)	2 (22.2)	13 (14.9)	**<0.001**
3 doses	0 (0.0)	0 (0.0)	1 (1.9)	4 (44.4)	24 (27.6)	**<0.001**
**Clinical statu*s*, n (%)**						
Mild	15 (31.2)	17 (40.5)	7 (13.0)	7 (77.8)	47 (54.0)	**<0.001**
Moderate	28 (58.3)	7 (16.7)	6 (11.1)	1 (11.1)	19 (21.8)	**<0.001**
Severe	2 (4.2)	10 (23.8)	12 (22.2)	0 (0)	13 (14.9)	**0.006**
Critical	3 (6.3)	8 (19.0)	29 (53.7)	1 (11.1)	8 (9.3)	**<0.001**
**Treatment duration (day), n (%)**						
<7	0 (0.0)	0 (0.0)	5 (9.3)	0 (0.0)	6 (6.9)	>0.05
7-14	1 (2.0)	17 (40.5)	30 (55.6)	5 (55.6)	68 (78.2)	**<0.001**
>14	47 (98.0)	25 (59.5)	19 (35.1)	4 (44.4)	13 (14.9)	**<0.001**
**Treatment outcome, n (%)**						
Cure	47 (98.0)	32 (76.2)	23 (42.6)	6 (66.7)	48 (55.2)	**<0.001**
Relief	1 (2.0)	4 (9.5)	10 (18.5)	2 (22.2)	36 (41.4)	**<0.001**
Death	0 (0.0)	6 (14.3)	21 (38.9)	1 (1.1)	3 (3.4)	**<0.001**

**Figure 1:**
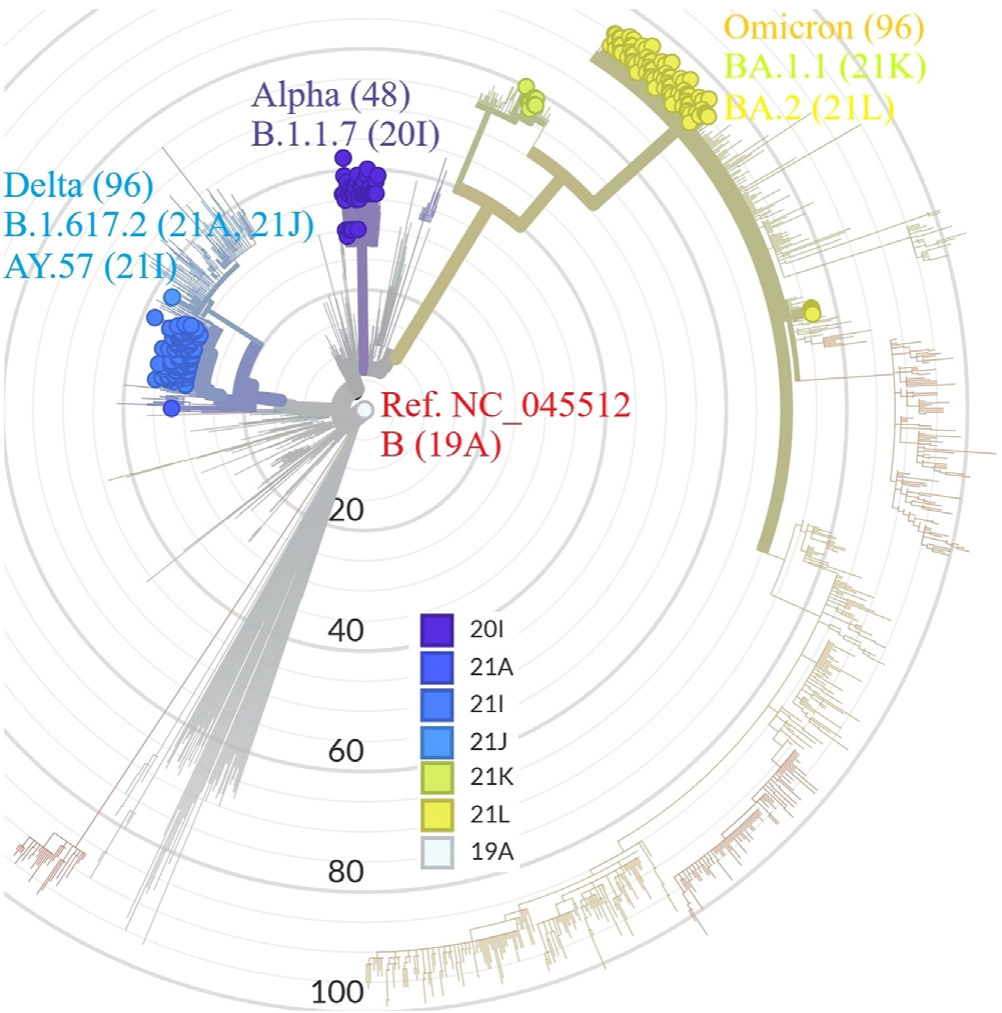
Analysis of the phylogenetic variations of SARS-CoV-2 Alpha, Delta, and Omicron. All SARS-CoV-2 are colored according to their assigned clade in the tree, which was created by the Nextstrain tool. The 2019 SARS-CoV-2 reference sequence in Wuhan is shown in red (accession number NC 045512), purple for Alpha, yellow and green for Omicron, and blue for Delta.

The nucleotide sequences of 48 Alpha, 96 Delta, and 96 Omicron variants were combined with global sequences to determine the location of Vietnam variants in a phylogenetic tree. The variants in this study are highlighted in blue (Delta variant), purple (Alpha), and yellow-green (Omicron) in the phylogenetic tree (Figure 1). The NextStrain clades are represented in different colors, with varying numbers of mutations. In this study, variants were found in three clades—Alpha, Delta, and Omicron—each of which comprised subclades, showing several occurrences of global transmission, followed by local transmission. This indicates the global nature of COVID-19, whereby SARS-CoV-2 swiftly spread worldwide after developing in a region of a certain country. Figure 1 displays the sequences of the Alpha, Delta, and Omicron variants in various hues that correspond to mutation numbers between 0 and 59. Only one mutation was found in the initial sequence from a student in Wuhan (situated in clade 19A) [12]. Other variations found in this study included Alpha (clade 20I), Delta (clades 21A, 21I, and 21J), and Omicron (clades 21K and 21L) variants.

### Clinical status and treatment outcomes of patients with COVID-19

In Alpha and Delta variants, 0% and 1.9% of the patients received all three doses of the vaccine, respectively; however, almost 50% of the patients with the Delta and Omicron variants were not vaccinated. Patients with the Omicron variant received vaccination at a higher rate than those with the Alpha and Delta variants, although the percentage of three-dose vaccination was low (<50%). Although these patients were unvaccinated, the clinical status of patients with the Alpha variant were mild (31.2%) and moderate (58.3%); approximately 10.5% of the patients progressed to severe or critical. Of the three variants, patients with the Delta variant had the highest risk of severe and critical progression (23.8-53.7%), although 33.3-57.1% of the patients received at least one dose of the vaccine. AY.57 had a significantly larger proportion of serious patients than B.1.617.2 (53.7% vs 19.0%) (Table 4). Patients with the Omicron variant had a lower rate of severe and critical status than those with the Delta variant (11.1% and 24.2%, respectively) but a higher rate than those with the Alpha variant. Treatment time for patients infected with the Alpha variant was 98% (>14 days), the Omicron variant 44.4% (BA.1.1) and 14.9% (BA.2), and the Delta variant 59.5% (B.1.617.2) and 35.1% (AY.57). Patients with the Delta variant had the highest death rates of 38.9% (AY.57) and 14.3% (B.1.617.2), whereas those with the Omicron variant had mortality rates of 1.1% (BA.1.1) and 3.4% (B.1.617.2) (BA.2). The Alpha variant had the highest percentage of patient recovery (98%); only 2.0% were in remission of symptoms, and no fatalities were found.

The model assessing the risk of mortality in patients with COVID-19 demonstrates that age (hazard ratio 1.05, 95% confidence interval 1.03-1.08]) and infection with the Delta variant (hazard ratio 12.49, 95% confidence interval 3.95-39.51) are independent factors for the prognosis of death in patients with COVID-19 (*P* <0.001). There was a statistically significant difference between the survival and death groups when comparing univariate comorbidity (*P* <0.001) (Supplement Table 2). When comorbidity was included in the multivariate regression analysis model, comorbidities had no predictive value in patients with COVID-19 (*P* >0.05). In the univariate analysis, the proportion of patients who received a full dose of vaccine in the death and survival groups did not differ significantly. Similarly, in multivariate regression analysis, this was not one of the indicators predicting mortality in patients with COVID-19 (Supplement Table 2).

Furthermore, we compared several clinical features, such as the rate of full immunization, illness severity, and treatment outcome, with a number of studies from different countries and found that the fatality rate of Delta variant in our study was 28.1%, whereas previous studies found fatality rates of 0.0%, 0.7%, 1.0%, and 5.0%, respectively (Supplement Table 3). Furthermore, the rate of severe and critical disease related to Delta variant infection was 61.5%, which is comparable to Radhakrishnan's research (62.3%) (Supplement Table 3). In our study, the rate of patients infected with Omicron with full vaccination was 44.8%, which is higher than the rates in the studies of Esper (73.3%) and Skarbinski (78%) but lower than Radhakrishman (10.7%) and Bahl (8.3%) (Supplement Table 3).

## Discussion

Only B.1.1.7 of 20I (V1) was detected in the Alpha variant but not in the other lineages (Q lineage), possibly owing to the predominance and popularity of B.1.1.7. The majority of B.1.1 was found by Al-Rashedi [13] in Iraq, Feng [14] in China, and Sander [15] in Benin. Because of its dominance, the B.1.1.7 lineage has become the most popular Alpha variant and was classified as a “variant of concern” and “variant being monitored.” Alpha has 31 mutations (six deletions and 25 substitutions), Delta has 29 mutations (five deletions and 24 substitutions), and Omicron has 59 mutations (10 deletions and 49 substitutions). When the mutant properties of Alpha, Delta, and Omicron variants were compared, a significant disparity was observed in terms of mutant types across the whole genome. However, several common mutations were found among them, including *N* (G2881A/T–R203K/M), *ORF1b* (C14408T–P314L), and *S* (A23403G–D614G and C23604A–P681H) (data not shown).

The Delta variant appeared in June 2021 with the two main lineages, AY.57 and B.1.617.2, which were first found in India and became variant of concern and variant being monitored shortly thereafter [16]. According to Zhan [17], Elliott [18], and Safari [19], the Delta variant appeared in the outbreak of the B.1.617.2 lineage. Our study recorded two lineages, AY.57 (21I) and B.1.617.2 (21A, 21J), belonging to the Delta variant in Vietnam during August 2021 to March 2022, although other studies have recorded the common lineage as B.1.617.2. At the NHTD, with sequencing data from thousands of samples, we found that the circulating current of the Delta variant was mainly B.1.617.2 only in the early stages of circulation. Thereafter, AY.57 was the most popular circulating line until it was replaced by the Omicron variant.

Compared with other reports, the Omicron variant had the largest genetic variety. The initial variant of Omicron was designated BA.1, and subsequent variants were named BA.2, BA.3, BA.4, and BA.5. *S* contains the most mutations in the B.1.1.529 lineage, with 25 mutations. Notably, *S* retained comparable alterations with the Alpha variant, such as N501Y and P681H, and the Delta variant, such as T478K [20]. We found that the Omicron variant has 57-59 mutations, with four, three, and three deletions in *S, ORF1a*, and *N*, respectively. In the Omicron variant, *S* has the most mutations (n = 26), followed by *ORF1a* (n = 12), *N* (n = 6), and *ORF1b* and *ORF9b* (n = 4 each). In fact, the virus exists as two types: sylvatic and urban. Studies on the genetic evolution of these two types have demonstrated that the mutation rate of urban type, which causes disease in humans, is faster than that of the sylvatic type, which is compatible with the level of circulatory behavior and rate of viral reproduction.

There are considerable inconsistencies between the data presented on the Pango lineage system and the genetic tree, particularly, for mutations in *S* in all three variants. Data from Weng et al. [21], Nikolaidis et al. [22], and Chakraborty et al*.* [23] have revealed the rate and type of mutation change among variants. We found several exclusive alterations in SARS-CoV-2 in this study; this is a transmission trait with a short viral life cycle that has been documented in several studies over the years.

Based on the examination of clinical signs and the diagnostic criteria of the Ministry of Health, Vietnam, there are four clinical levels: mild, moderate, severe, and critical. Despite not being vaccinated, the number of patients infected with the Alpha variant with severe and critical status was substantially lower than those infected with the Delta variant, particularly, the AY.57 lineage, with 22.2% and 53.7% of the patients in the severe and critical category, respectively. The same was observed in the group of patients with the Omicron variant. Our findings are consistent with reports on the severity of infection with various SARS-CoV-2 variants—the Delta variant had a 1.85-3.0–fold higher incidence of severe transmission than the Alpha variant [24]. Vaccination against COVID-19 was shown to be unrelated to disease severity and patient mortality for Alpha, Delta, and Omicron variants. These data are similar to those reported by Hyams [25] and Lauring [26].

The number of patients with the Alpha variant who recovered was quite high (98%), whereas the recovery rate ranged from 42.6% to 76.2% for Delta and Omicron infection, respectively. Patients with the B.1.617.2 variant recovered faster than those with the AY.57, as well as BA.1.1 and BA.2, variant. However, the Delta variant had mortality rates of 38.9% (AY.57) and 14.3% (B.1.617.2)—significantly higher than that of the Omicron variant (BA.1.1: 1.1% and BA.2: 3.4%). According to data from Lauring et al*.* [26], the proportion of adult patients who died from infection with the Delta variant (13.2%) was higher than that of patients with the Alpha (4.3%) and Omicron (5.1%) variants, and there was no association between vaccination and death. Nevertheless, in a study of pediatric patients conducted by Bahl et al*.* [27], there was no difference in mortality between Alpha, Delta, and Omicron variants. Li et al. [28] found no difference in the rate of critical patients between the Delta and Omicron variants in patients aged <17 years; however, in patients aged >18 years, the Delta variant exhibited a much higher rate of severe status (29.9%) than the Omicron variant (10.3%).

In this study, we developed a model to assess the risk of death in patients with COVID-19 and found that older age and infection with the Delta variant are two independent factors that are useful for predicting death. Comorbidities and full vaccination are not factors in the deaths of patients with COVID-19. Hu et al. [29] reported that older age was an independent factor in severe and fatal cases. Radhakrishnan et al. [30] found that infection with the Delta variant resulted in a higher mortality rate than infection with other variants. Therefore, developing a clinical model to predict outcome in patients with COVID-19, particularly, with severe and critical clinical circumstances, is crucial.

Our study has several limitations. First, the sample size is small because many sequencing samples were not completed because of the duration of the circulating Alpha variant; therefore, only 48 Alpha samples were analyzed. Second, owing to the lack of data, there has not been an in-depth analysis of the relationship between the genomic features of SARS-CoV-2 and several clinical features, such as degree of fever, respiratory system damage, complications, changes in the parathyroid index, and clinical features (biochemistry, hematology, and immunology).

In conclusion, our study demonstrated that the Alpha, Delta, and Omicron variants exhibited alterations in many genes, with especially high rates in *S* and *ORF1a*, with varied rates and types of mutations among the variants worldwide. Severe, critical, and fatal cases were observed only for the Delta and Omicron variants, with a significantly higher incidence with the Delta variant than the Omicron variant. AY.57 (Delta) had significantly higher rates of severe, critical, and fatal cases than B.1.617.2 (Delta) and BA.1.1 and BA.2 (Omicron). Old age and infection with the Delta variant were independent predictors of mortality in patients with COVID-19.

## Declarations of competing interest

The authors have no competing interests to declare.

## Appendix Supplementary materials

Image, application 1

Image, application 2

## References

[1] Hu B, Guo H, Zhou P, Shi ZL. (2021). Characteristics of SARS-CoV-2 and COVID-19. Nat Rev Microbiol.

[2] World Health Organization. Coronavirus (COVID-19) dashboard. Geneva: World Health Organization, 2023.

[3] Minh LHN, Khoi Quan N, Le TN, Khanh PNQ, Huy NT (2021). COVID-19 timeline of Vietnam: important milestones through four waves of the pandemic and lesson learned. Front Public Health.

[4] Oran DP, Topol EJ. (2020). Prevalence of asymptomatic SARS-CoV-2 infection: a narrative review. Ann Intern Med.

[5] Backer JA, Klinkenberg D, Wallinga J. (2020). Incubation period of 2019 novel coronavirus (2019-nCoV) infections among travellers from Wuhan, China, 20–28 January 2020. Euro Surveill.

[6] Irizar DPP, Kapadia D, Bécares L, Sze S, Taylor H, Amele S (2023). Ethnic inequalities in COVID-19 infection, hospitalisation, intensive care admission, and death: a global systematic review and meta-analysis of over 200 million study participants. EClinicalMedicine.

[7] Naqvia KFA, Mohammada T, Fatimac U, Singhd IK, Singhe A, Atiff SM (2020). Insights into SARS-CoV-2 genome, structure, evolution, pathogenesis and therapies: structural genomics approach. BBA Mol Basis Dis.

[8] Peck KM, Lauring AS. (2018). Complexities of viral mutation rates. J Virol.

[9] Maurya R, Mishra P, Swaminathan A, Ravi V, Saifi S, Kanakan A (2022). SARS-CoV-2 mutations and COVID-19 clinical outcome: mutation global frequency dynamics and structural modulation hold the key. Front Cell Infect Microbiol.

[10] Lipsitch M, Krammer F, Regev-Yochay G, Lustig Y, Balicer RD. (2022). SARS-CoV-2 breakthrough infections in vaccinated individuals: measurement, causes and impact. Nat Rev Immunol.

[11] World Health Organization (2020).

[12] Nguyen TT, Pham TN, TD Van, Nguyen TT, Nguyen DTN, Le HNM (2020). Genetic diversity of SARS-CoV-2 and clinical, epidemiological characteristics of COVID-19 patients in Hanoi, Vietnam. PLoS One.

[13] Al-Rashedi NAM, Alburkat H, Hadi AO, Munahi MG, Jasim A, Hameed A (2022). High prevalence of an alpha variant lineage with a premature stop codon in ORF7a in Iraq, winter 2020-2021. PLoS One.

[14] Feng Z, Cui S, Lyu B, Liang Z, Li F, Shen L (2022). Genomic characteristics of SARS-CoV-2 in Beijing, China, 2021. Biosaf Health.

[15] Sander AL, Yadouleton A, de Oliveira Filho EF, Tchibozo C, Hounkanrin G, Badou Y (2021). Mutations associated with SARS-CoV-2 variants of concern, Benin, early 2021. Emerg Infect Dis.

[16] Chavda VP, Bezbaruah R, Deka K, Nongrang L, Kalita T. (2022). The delta and omicron variants of SARS-CoV-2: what we know so far. Vaccines (Basel).

[17] Zhan Y, Yin H, Yin JY. (2022). B.1.617.2 (Delta) Variant of SARS-CoV-2: features, transmission and potential strategies. Int J Biol Sci.

[18] Elliott P, Haw D, Wang H, Eales O, Walters CE, Ainslie KEC (2021). Exponential growth, high prevalence of SARS-CoV-2, and vaccine effectiveness associated with the Delta variant. Science.

[19] Safari I, Elahi E. (2022). Evolution of the SARS-CoV-2 genome and emergence of variants of concern. Arch Virol.

[20] Manjunath R, Gaonkar SL, Saleh EAM, Husain K. (2022). A comprehensive review on Covid-19 Omicron (B.1.1.529) variant. Saudi J Biol Sci.

[21] Weng S, Shang J, Cheng Y, Zhou H, Ji C, Yang R (2022). Genetic differentiation and diversity of SARS-CoV-2 Omicron variant in its early outbreak. Biosaf Health.

[22] Nikolaidis M, Papakyriakou A, Chlichlia K, Markoulatos P, Oliver SG, Amoutzias GD. (2022). Comparative analysis of SARS-CoV-2 variants of concern, including omicron, highlights their common and distinctive amino acid substitution patterns, especially at the spike ORF. Viruses.

[23] Chakraborty C, Bhattacharya M, Sharma AR, Dhama K, Lee SS. (2022). Continent-wide evolutionary trends of emerging SARS-CoV-2 variants: dynamic profiles from Alpha to Omicron. GeroScience.

[24] Twohig KA, Nyberg T, Zaidi A, Thelwall S, Sinnathamby MA, Aliabadi S (2022). Hospital admission and emergency care attendance risk for SARS-CoV-2 delta (B.1.617.2) compared with alpha (B.1.1.7) variants of concern: a cohort study. Lancet Infect Dis.

[25] Hyams C, Challen R, Marlow R, Nguyen J, Begier E, Southern J (2023). Severity of Omicron (B.1.1.529) and Delta (B.1.617.2) SARS-CoV-2 infection among hospitalised adults: a prospective cohort study in Bristol, United Kingdom. Lancet Reg Health Eur.

[26] Lauring AS, Tenforde MW, Chappell JD, Gaglani M, Ginde AA, McNeal T (2022). Clinical severity of, and effectiveness of mRNA vaccines against, Covid-19 from omicron, delta, and alpha SARS-CoV-2 variants in the United States: prospective observational study. BMJ.

[27] Bahl A, Mielke N, Johnson S, Desai A, Qu L. (2023). Severe COVID-19 outcomes in pediatrics: an observational cohort analysis comparing Alpha, Delta, and Omicron variants. Lancet Reg Health Am.

[28] Li M, Liu Q, Wu D, Tang L, Wang X, Yan T (2022). Association of COVID-19 vaccination and clinical severity of patients infected with delta or omicron variants - China, May 21, 2021–February 28, 2022. China CDC Wkly.

[29] Hu C, Liu Z, Jiang Y, Shi O, Zhang X, Xu K (2021). Early prediction of mortality risk among patients with severe COVID-19, using machine learning. Int J Epidemiol.

[30] Radhakrishnan N, Liu M, Idowu B, Bansari A, Rathi K, Magar S (2023). Comparison of the clinical characteristics of SARS-CoV-2 Delta (B.1.617.2) and Omicron (B.1.1.529) infected patients from a single hospitalist service. BMC Infect Dis.

