# Supplementary material for: Genetic features of SARS-CoV-2 Alpha, Delta, and Omicron variants and their association with the clinical severity of COVID-19 in Vietnam
Source: IJID Reg. 2024 Mar 13;11:100348. doi: 10.1016/j.ijregi.2024.03.003 (PMC11004080; doi:10.1016/j.ijregi.2024.03.003)
Supplement: Supplementary file 1 [file mmc1.docx]

**Supplement data**

Reference sequences for SARS-CoV-2 variant comparisons between Vietnam and other countries

1. **Alpha**

- China

| EPI_ISL_2405168 | EPI_ISL_2405172 | EPI_ISL_2405173 | EPI_ISL_2405177 | EPI_ISL_2432957 | EPI_ISL_8582079 | EPI_ISL_9910219 |
| --- | --- | --- | --- | --- | --- | --- |
| EPI_ISL_2405169 | EPI_ISL_8582073 | EPI_ISL_2405174 | EPI_ISL_2426143 | EPI_ISL_2438545 | EPI_ISL_8582080 | EPI_ISL_11799982 |
| EPI_ISL_2405170 | EPI_ISL_2432956 | EPI_ISL_2405175 | EPI_ISL_2432955 | EPI_ISL_2500849 | EPI_ISL_9910211 | EPI_ISL_16405160 |
| EPI_ISL_2405171 | EPI_ISL_2405176 |  |  |  |  |  |

- Ghana

| EPI_ISL_1255264 | EPI_ISL_2422574 | EPI_ISL_2836860 | EPI_ISL_2361911 | EPI_ISL_2873870 | EPI_ISL_3268103 | EPI_ISL_8065527 |
| --- | --- | --- | --- | --- | --- | --- |
| EPI_ISL_1255265 | EPI_ISL_2422575 | EPI_ISL_2836867 | EPI_ISL_2361912 | EPI_ISL_2873871 | EPI_ISL_3268104 | EPI_ISL_8065528 |
| EPI_ISL_1255266 | EPI_ISL_2422577 | EPI_ISL_2836871 | EPI_ISL_2361914 | EPI_ISL_2873872 | EPI_ISL_4602029 | EPI_ISL_8065529 |
| EPI_ISL_1255267 | EPI_ISL_2422578 | EPI_ISL_2836874 | EPI_ISL_2361915 | EPI_ISL_2873873 | EPI_ISL_4602031 | EPI_ISL_8065535 |
| EPI_ISL_1255269 | EPI_ISL_2422579 | EPI_ISL_2836878 | EPI_ISL_2361916 | EPI_ISL_2968000 | EPI_ISL_4602040 | EPI_ISL_8065537 |
| EPI_ISL_1255271 | EPI_ISL_2422581 | EPI_ISL_2836881 | EPI_ISL_2361917 | EPI_ISL_2535790 | EPI_ISL_4602041 | EPI_ISL_8065579 |
| EPI_ISL_1255272 | EPI_ISL_2422582 | EPI_ISL_2836896 | EPI_ISL_2361919 | EPI_ISL_2535792 | EPI_ISL_4602042 | EPI_ISL_8065581 |
| EPI_ISL_1255273 | EPI_ISL_2422583 | EPI_ISL_2836898 | EPI_ISL_2361920 | EPI_ISL_2535793 | EPI_ISL_4602050 | EPI_ISL_9702365 |
| EPI_ISL_1255275 | EPI_ISL_2422585 | EPI_ISL_2836899 | EPI_ISL_2376386 | EPI_ISL_2535795 | EPI_ISL_4602059 | EPI_ISL_9702366 |
| EPI_ISL_1255277 | EPI_ISL_2422586 | EPI_ISL_2836904 | EPI_ISL_2376387 | EPI_ISL_2535796 | EPI_ISL_4602060 | EPI_ISL_9702367 |
| EPI_ISL_2001074 | EPI_ISL_2422588 | EPI_ISL_2836905 | EPI_ISL_2376388 | EPI_ISL_4602046 | EPI_ISL_4602064 | EPI_ISL_9702368 |
| EPI_ISL_2001075 | EPI_ISL_2422589 | EPI_ISL_2836906 | EPI_ISL_2422501 | EPI_ISL_2968006 | EPI_ISL_4602065 | EPI_ISL_9702370 |
| EPI_ISL_2001076 | EPI_ISL_2422593 | EPI_ISL_2836950 | EPI_ISL_2422504 | EPI_ISL_2968022 | EPI_ISL_4602066 | EPI_ISL_9702371 |
| EPI_ISL_2001079 | EPI_ISL_2422604 | EPI_ISL_2864611 | EPI_ISL_2422511 | EPI_ISL_2968026 | EPI_ISL_4602070 | EPI_ISL_9702372 |
| EPI_ISL_2001080 | EPI_ISL_2422607 | EPI_ISL_2864613 | EPI_ISL_2422512 | EPI_ISL_2535798 | EPI_ISL_4602071 | EPI_ISL_9702373 |
| EPI_ISL_2001081 | EPI_ISL_2422608 | EPI_ISL_2864621 | EPI_ISL_2422517 | EPI_ISL_2535800 | EPI_ISL_4602086 | EPI_ISL_9702374 |
| EPI_ISL_2001082 | EPI_ISL_2422615 | EPI_ISL_2864628 | EPI_ISL_2422518 | EPI_ISL_2535802 | EPI_ISL_4602087 | EPI_ISL_9702375 |
| EPI_ISL_2001083 | EPI_ISL_2422618 | EPI_ISL_2864638 | EPI_ISL_2422519 | EPI_ISL_2535803 | EPI_ISL_4602089 | EPI_ISL_9702378 |
| EPI_ISL_2001084 | EPI_ISL_2422621 | EPI_ISL_2864650 | EPI_ISL_2422533 | EPI_ISL_2535804 | EPI_ISL_5334609 | EPI_ISL_9702379 |
| EPI_ISL_2001085 | EPI_ISL_2422627 | EPI_ISL_2864652 | EPI_ISL_2422534 | EPI_ISL_2535805 | EPI_ISL_5334610 | EPI_ISL_9702380 |
| EPI_ISL_2001086 | EPI_ISL_2508373 | EPI_ISL_2864659 | EPI_ISL_2422536 | EPI_ISL_2535806 | EPI_ISL_5334611 | EPI_ISL_9702384 |
| EPI_ISL_2001087 | EPI_ISL_2508374 | EPI_ISL_2864661 | EPI_ISL_2422537 | EPI_ISL_2535807 | EPI_ISL_5334612 | EPI_ISL_9702396 |
| EPI_ISL_2001088 | EPI_ISL_2508377 | EPI_ISL_2864663 | EPI_ISL_2422542 | EPI_ISL_2535808 | EPI_ISL_5334613 | EPI_ISL_9702402 |
| EPI_ISL_2001089 | EPI_ISL_2508379 | EPI_ISL_2864665 | EPI_ISL_2422544 | EPI_ISL_2535809 | EPI_ISL_5334614 | EPI_ISL_9702404 |
| EPI_ISL_2001090 | EPI_ISL_2508380 | EPI_ISL_2864669 | EPI_ISL_2422545 | EPI_ISL_2535812 | EPI_ISL_5751084 | EPI_ISL_9702405 |
| EPI_ISL_2001091 | EPI_ISL_2508381 | EPI_ISL_2864671 | EPI_ISL_2422546 | EPI_ISL_2535813 | EPI_ISL_5751093 | EPI_ISL_9702406 |
| EPI_ISL_2001092 | EPI_ISL_2508382 | EPI_ISL_2864676 | EPI_ISL_2422547 | EPI_ISL_2535815 | EPI_ISL_5751094 | EPI_ISL_9702412 |
| EPI_ISL_2001093 | EPI_ISL_2508383 | EPI_ISL_2864684 | EPI_ISL_2422550 | EPI_ISL_2535816 | EPI_ISL_5751097 | EPI_ISL_9980064 |
| EPI_ISL_2001095 | EPI_ISL_2508384 | EPI_ISL_2873840 | EPI_ISL_2422551 | EPI_ISL_2535817 | EPI_ISL_5751099 | EPI_ISL_10334556 |
| EPI_ISL_2001098 | EPI_ISL_2508385 | EPI_ISL_2873842 | EPI_ISL_2422553 | EPI_ISL_2535818 | EPI_ISL_5751107 | EPI_ISL_10334559 |
| EPI_ISL_2285840 | EPI_ISL_2508387 | EPI_ISL_2873843 | EPI_ISL_2422554 | EPI_ISL_2535819 | EPI_ISL_5751112 | EPI_ISL_10334561 |
| EPI_ISL_2285842 | EPI_ISL_2508388 | EPI_ISL_2873844 | EPI_ISL_2422556 | EPI_ISL_2535820 | EPI_ISL_5751121 | EPI_ISL_10334563 |
| EPI_ISL_2285843 | EPI_ISL_2508389 | EPI_ISL_2873845 | EPI_ISL_2422557 | EPI_ISL_2535821 | EPI_ISL_5751142 | EPI_ISL_10334568 |
| EPI_ISL_2285844 | EPI_ISL_2508390 | EPI_ISL_2873847 | EPI_ISL_2422558 | EPI_ISL_2535823 | EPI_ISL_5751161 | EPI_ISL_10334607 |
| EPI_ISL_2285850 | EPI_ISL_2508391 | EPI_ISL_2873848 | EPI_ISL_2422560 | EPI_ISL_2535824 | EPI_ISL_5751170 | EPI_ISL_10334612 |
| EPI_ISL_2285851 | EPI_ISL_2508392 | EPI_ISL_2873849 | EPI_ISL_2422561 | EPI_ISL_2535825 | EPI_ISL_5751176 | EPI_ISL_10334615 |
| EPI_ISL_2285852 | EPI_ISL_2508393 | EPI_ISL_2873850 | EPI_ISL_2422565 | EPI_ISL_2535826 | EPI_ISL_5751178 | EPI_ISL_10438324 |
| EPI_ISL_2285853 | EPI_ISL_2508394 | EPI_ISL_2873853 | EPI_ISL_2422566 | EPI_ISL_2535827 | EPI_ISL_5751179 | EPI_ISL_10438335 |
| EPI_ISL_2285855 | EPI_ISL_2508395 | EPI_ISL_2873854 | EPI_ISL_2422568 | EPI_ISL_2535828 | EPI_ISL_5751186 | EPI_ISL_10438336 |
| EPI_ISL_2285864 | EPI_ISL_2508396 | EPI_ISL_2873855 | EPI_ISL_2422570 | EPI_ISL_2535829 | EPI_ISL_5751193 | EPI_ISL_10438349 |
| EPI_ISL_2285865 | EPI_ISL_2535782 | EPI_ISL_2873857 | EPI_ISL_2422571 | EPI_ISL_2535830 | EPI_ISL_5751201 | EPI_ISL_10438357 |
| EPI_ISL_2285867 | EPI_ISL_2535783 | EPI_ISL_2873859 | EPI_ISL_2422572 | EPI_ISL_2535831 | EPI_ISL_5751204 | EPI_ISL_10438373 |
| EPI_ISL_2285868 | EPI_ISL_2535784 | EPI_ISL_2873860 | EPI_ISL_2836852 | EPI_ISL_2535832 | EPI_ISL_5751215 | EPI_ISL_10438378 |
| EPI_ISL_2285869 | EPI_ISL_2535785 | EPI_ISL_2873862 | EPI_ISL_2836856 | EPI_ISL_2535833 | EPI_ISL_6088176 | EPI_ISL_10438401 |
| EPI_ISL_2361904 | EPI_ISL_2535786 | EPI_ISL_2873863 | EPI_ISL_2836857 | EPI_ISL_2535834 | EPI_ISL_6088231 | EPI_ISL_10438404 |
| EPI_ISL_2361906 | EPI_ISL_2535787 | EPI_ISL_2873866 | EPI_ISL_4602044 | EPI_ISL_2535835 | EPI_ISL_6088269 | EPI_ISL_10438558 |
| EPI_ISL_2361908 | EPI_ISL_2535788 | EPI_ISL_2873868 | EPI_ISL_8065521 | EPI_ISL_2535836 | EPI_ISL_6088300 | EPI_ISL_15887002 |
| EPI_ISL_2361909 | EPI_ISL_2535789 | EPI_ISL_2873869 | EPI_ISL_8065524 | EPI_ISL_2535837 | EPI_ISL_6088309 | EPI_ISL_15887005 |
| EPI_ISL_18042704 | EPI_ISL_8065519 | EPI_ISL_15887013 | EPI_ISL_8065520 | EPI_ISL_15887017 | EPI_ISL_6088350 | EPI_ISL_15887006 |
| EPI_ISL_18508185 |  |  |  |  |  |  |

- England

| EPI_ISL_924084 | EPI_ISL_945009 | EPI_ISL_945729 | EPI_ISL_945939 | EPI_ISL_946208 | EPI_ISL_949669 | EPI_ISL_956426 |
| --- | --- | --- | --- | --- | --- | --- |
| EPI_ISL_924101 | EPI_ISL_945010 | EPI_ISL_945730 | EPI_ISL_945940 | EPI_ISL_946210 | EPI_ISL_949670 | EPI_ISL_956428 |
| EPI_ISL_924108 | EPI_ISL_945012 | EPI_ISL_945733 | EPI_ISL_945944 | EPI_ISL_946211 | EPI_ISL_949717 | EPI_ISL_956429 |
| EPI_ISL_924128 | EPI_ISL_945013 | EPI_ISL_945735 | EPI_ISL_945945 | EPI_ISL_946213 | EPI_ISL_949719 | EPI_ISL_956431 |
| EPI_ISL_924164 | EPI_ISL_945015 | EPI_ISL_945736 | EPI_ISL_945950 | EPI_ISL_946215 | EPI_ISL_949728 | EPI_ISL_956432 |
| EPI_ISL_924176 | EPI_ISL_945021 | EPI_ISL_945737 | EPI_ISL_945951 | EPI_ISL_946218 | EPI_ISL_949795 | EPI_ISL_956433 |
| EPI_ISL_924215 | EPI_ISL_945022 | EPI_ISL_945738 | EPI_ISL_945954 | EPI_ISL_946220 | EPI_ISL_949796 | EPI_ISL_956434 |
| EPI_ISL_924245 | EPI_ISL_945024 | EPI_ISL_945741 | EPI_ISL_945958 | EPI_ISL_946224 | EPI_ISL_949801 | EPI_ISL_956436 |
| EPI_ISL_924252 | EPI_ISL_945028 | EPI_ISL_945742 | EPI_ISL_945961 | EPI_ISL_946225 | EPI_ISL_949803 | EPI_ISL_956437 |
| EPI_ISL_924265 | EPI_ISL_945031 | EPI_ISL_945743 | EPI_ISL_945962 | EPI_ISL_946227 | EPI_ISL_949804 | EPI_ISL_956438 |
| EPI_ISL_924279 | EPI_ISL_945035 | EPI_ISL_945744 | EPI_ISL_945963 | EPI_ISL_946229 | EPI_ISL_949805 | EPI_ISL_956439 |
| EPI_ISL_924302 | EPI_ISL_945039 | EPI_ISL_945747 | EPI_ISL_945965 | EPI_ISL_946231 | EPI_ISL_949806 | EPI_ISL_956440 |
| EPI_ISL_924384 | EPI_ISL_945042 | EPI_ISL_945753 | EPI_ISL_945967 | EPI_ISL_946232 | EPI_ISL_949807 | EPI_ISL_956441 |
| EPI_ISL_944793 | EPI_ISL_945044 | EPI_ISL_945755 | EPI_ISL_945969 | EPI_ISL_946233 | EPI_ISL_949808 | EPI_ISL_956442 |
| EPI_ISL_944795 | EPI_ISL_945048 | EPI_ISL_945757 | EPI_ISL_945971 | EPI_ISL_946234 | EPI_ISL_949809 | EPI_ISL_956444 |
| EPI_ISL_944796 | EPI_ISL_945374 | EPI_ISL_945758 | EPI_ISL_945972 | EPI_ISL_946243 | EPI_ISL_949810 | EPI_ISL_956445 |
| EPI_ISL_944802 | EPI_ISL_945375 | EPI_ISL_945759 | EPI_ISL_945976 | EPI_ISL_946254 | EPI_ISL_949915 | EPI_ISL_956447 |
| EPI_ISL_944803 | EPI_ISL_945376 | EPI_ISL_945763 | EPI_ISL_945980 | EPI_ISL_946260 | EPI_ISL_949916 | EPI_ISL_956451 |
| EPI_ISL_944817 | EPI_ISL_945379 | EPI_ISL_945768 | EPI_ISL_945983 | EPI_ISL_946264 | EPI_ISL_949918 | EPI_ISL_956453 |
| EPI_ISL_944823 | EPI_ISL_945386 | EPI_ISL_945771 | EPI_ISL_945984 | EPI_ISL_946270 | EPI_ISL_949921 | EPI_ISL_956454 |
| EPI_ISL_944825 | EPI_ISL_945387 | EPI_ISL_945772 | EPI_ISL_945988 | EPI_ISL_946271 | EPI_ISL_949922 | EPI_ISL_956458 |
| EPI_ISL_944827 | EPI_ISL_945389 | EPI_ISL_945776 | EPI_ISL_945990 | EPI_ISL_946274 | EPI_ISL_949923 | EPI_ISL_956459 |
| EPI_ISL_944828 | EPI_ISL_945402 | EPI_ISL_945777 | EPI_ISL_945993 | EPI_ISL_946281 | EPI_ISL_949936 | EPI_ISL_956460 |
| EPI_ISL_944829 | EPI_ISL_945408 | EPI_ISL_945781 | EPI_ISL_945994 | EPI_ISL_946284 | EPI_ISL_949949 | EPI_ISL_956461 |
| EPI_ISL_944831 | EPI_ISL_945419 | EPI_ISL_945788 | EPI_ISL_945997 | EPI_ISL_946285 | EPI_ISL_950173 | EPI_ISL_956462 |
| EPI_ISL_944833 | EPI_ISL_945420 | EPI_ISL_945792 | EPI_ISL_945998 | EPI_ISL_946286 | EPI_ISL_950177 | EPI_ISL_956466 |
| EPI_ISL_944834 | EPI_ISL_945422 | EPI_ISL_945795 | EPI_ISL_945999 | EPI_ISL_946292 | EPI_ISL_950195 | EPI_ISL_956469 |
| EPI_ISL_944836 | EPI_ISL_945423 | EPI_ISL_945796 | EPI_ISL_946000 | EPI_ISL_946298 | EPI_ISL_950198 | EPI_ISL_956472 |
| EPI_ISL_944837 | EPI_ISL_945433 | EPI_ISL_945799 | EPI_ISL_946002 | EPI_ISL_946301 | EPI_ISL_950200 | EPI_ISL_956474 |
| EPI_ISL_944839 | EPI_ISL_945444 | EPI_ISL_945800 | EPI_ISL_946005 | EPI_ISL_946302 | EPI_ISL_950201 | EPI_ISL_956475 |
| EPI_ISL_944845 | EPI_ISL_945446 | EPI_ISL_945802 | EPI_ISL_946006 | EPI_ISL_946304 | EPI_ISL_950203 | EPI_ISL_956476 |
| EPI_ISL_944848 | EPI_ISL_945449 | EPI_ISL_945803 | EPI_ISL_946007 | EPI_ISL_946319 | EPI_ISL_950206 | EPI_ISL_956477 |
| EPI_ISL_944849 | EPI_ISL_945458 | EPI_ISL_945804 | EPI_ISL_946008 | EPI_ISL_946326 | EPI_ISL_950208 | EPI_ISL_956478 |
| EPI_ISL_944854 | EPI_ISL_945459 | EPI_ISL_945808 | EPI_ISL_946009 | EPI_ISL_946328 | EPI_ISL_950210 | EPI_ISL_956479 |
| EPI_ISL_944856 | EPI_ISL_945466 | EPI_ISL_945809 | EPI_ISL_946010 | EPI_ISL_946333 | EPI_ISL_950402 | EPI_ISL_956480 |
| EPI_ISL_944861 | EPI_ISL_945467 | EPI_ISL_945812 | EPI_ISL_946014 | EPI_ISL_946335 | EPI_ISL_950403 | EPI_ISL_956481 |
| EPI_ISL_944867 | EPI_ISL_945468 | EPI_ISL_945813 | EPI_ISL_946021 | EPI_ISL_946338 | EPI_ISL_950404 | EPI_ISL_956483 |
| EPI_ISL_944872 | EPI_ISL_945469 | EPI_ISL_945816 | EPI_ISL_946023 | EPI_ISL_946339 | EPI_ISL_950405 | EPI_ISL_956485 |
| EPI_ISL_944874 | EPI_ISL_945475 | EPI_ISL_945817 | EPI_ISL_946026 | EPI_ISL_946351 | EPI_ISL_950406 | EPI_ISL_956488 |
| EPI_ISL_944879 | EPI_ISL_945480 | EPI_ISL_945818 | EPI_ISL_946028 | EPI_ISL_946356 | EPI_ISL_950407 | EPI_ISL_956490 |
| EPI_ISL_944882 | EPI_ISL_945485 | EPI_ISL_945821 | EPI_ISL_946030 | EPI_ISL_946357 | EPI_ISL_950425 | EPI_ISL_956492 |
| EPI_ISL_944884 | EPI_ISL_945490 | EPI_ISL_945822 | EPI_ISL_946037 | EPI_ISL_946360 | EPI_ISL_950646 | EPI_ISL_956493 |
| EPI_ISL_944885 | EPI_ISL_945494 | EPI_ISL_945824 | EPI_ISL_946041 | EPI_ISL_946361 | EPI_ISL_950647 | EPI_ISL_956494 |
| EPI_ISL_944887 | EPI_ISL_945498 | EPI_ISL_945826 | EPI_ISL_946048 | EPI_ISL_946365 | EPI_ISL_950648 | EPI_ISL_956496 |
| EPI_ISL_944888 | EPI_ISL_945502 | EPI_ISL_945828 | EPI_ISL_946049 | EPI_ISL_946366 | EPI_ISL_950649 | EPI_ISL_956497 |
| EPI_ISL_944895 | EPI_ISL_945506 | EPI_ISL_945831 | EPI_ISL_946052 | EPI_ISL_946367 | EPI_ISL_950650 | EPI_ISL_956499 |
| EPI_ISL_944900 | EPI_ISL_945508 | EPI_ISL_945835 | EPI_ISL_946055 | EPI_ISL_946371 | EPI_ISL_950651 | EPI_ISL_956500 |
| EPI_ISL_944909 | EPI_ISL_945514 | EPI_ISL_945841 | EPI_ISL_946057 | EPI_ISL_946374 | EPI_ISL_950762 | EPI_ISL_956503 |
| EPI_ISL_944910 | EPI_ISL_945518 | EPI_ISL_945843 | EPI_ISL_946058 | EPI_ISL_946375 | EPI_ISL_951974 | EPI_ISL_956504 |
| EPI_ISL_944913 | EPI_ISL_945522 | EPI_ISL_945844 | EPI_ISL_946066 | EPI_ISL_946378 | EPI_ISL_951985 | EPI_ISL_956507 |
| EPI_ISL_944917 | EPI_ISL_945525 | EPI_ISL_945846 | EPI_ISL_946067 | EPI_ISL_946379 | EPI_ISL_951986 | EPI_ISL_956509 |
| EPI_ISL_944918 | EPI_ISL_945529 | EPI_ISL_945848 | EPI_ISL_946068 | EPI_ISL_946380 | EPI_ISL_952042 | EPI_ISL_956510 |
| EPI_ISL_944919 | EPI_ISL_945539 | EPI_ISL_945849 | EPI_ISL_946072 | EPI_ISL_946385 | EPI_ISL_952043 | EPI_ISL_956511 |
| EPI_ISL_944924 | EPI_ISL_945543 | EPI_ISL_945850 | EPI_ISL_946086 | EPI_ISL_946391 | EPI_ISL_952044 | EPI_ISL_956513 |
| EPI_ISL_944928 | EPI_ISL_945545 | EPI_ISL_945851 | EPI_ISL_946087 | EPI_ISL_946397 | EPI_ISL_952045 | EPI_ISL_956514 |
| EPI_ISL_944930 | EPI_ISL_945548 | EPI_ISL_945853 | EPI_ISL_946088 | EPI_ISL_946400 | EPI_ISL_952047 | EPI_ISL_956516 |
| EPI_ISL_944933 | EPI_ISL_945549 | EPI_ISL_945860 | EPI_ISL_946090 | EPI_ISL_946405 | EPI_ISL_952048 | EPI_ISL_956517 |
| EPI_ISL_944934 | EPI_ISL_945555 | EPI_ISL_945862 | EPI_ISL_946093 | EPI_ISL_946407 | EPI_ISL_952049 | EPI_ISL_956518 |
| EPI_ISL_944944 | EPI_ISL_945561 | EPI_ISL_945863 | EPI_ISL_946096 | EPI_ISL_946410 | EPI_ISL_952050 | EPI_ISL_956519 |
| EPI_ISL_944945 | EPI_ISL_945563 | EPI_ISL_945865 | EPI_ISL_946106 | EPI_ISL_946411 | EPI_ISL_952051 | EPI_ISL_956520 |
| EPI_ISL_944947 | EPI_ISL_945569 | EPI_ISL_945867 | EPI_ISL_946109 | EPI_ISL_949405 | EPI_ISL_952053 | EPI_ISL_956521 |
| EPI_ISL_944951 | EPI_ISL_945579 | EPI_ISL_945868 | EPI_ISL_946110 | EPI_ISL_949543 | EPI_ISL_952058 | EPI_ISL_956523 |
| EPI_ISL_944955 | EPI_ISL_945582 | EPI_ISL_945869 | EPI_ISL_946112 | EPI_ISL_949545 | EPI_ISL_952059 | EPI_ISL_956524 |
| EPI_ISL_944956 | EPI_ISL_945587 | EPI_ISL_945870 | EPI_ISL_946113 | EPI_ISL_949547 | EPI_ISL_952060 | EPI_ISL_956525 |
| EPI_ISL_944959 | EPI_ISL_945591 | EPI_ISL_945871 | EPI_ISL_946115 | EPI_ISL_949551 | EPI_ISL_952957 | EPI_ISL_956527 |
| EPI_ISL_944961 | EPI_ISL_945597 | EPI_ISL_945872 | EPI_ISL_946123 | EPI_ISL_949552 | EPI_ISL_952959 | EPI_ISL_956528 |
| EPI_ISL_944962 | EPI_ISL_945621 | EPI_ISL_945873 | EPI_ISL_946124 | EPI_ISL_949567 | EPI_ISL_952986 | EPI_ISL_956530 |
| EPI_ISL_944963 | EPI_ISL_945627 | EPI_ISL_945874 | EPI_ISL_946126 | EPI_ISL_949575 | EPI_ISL_952988 | EPI_ISL_956531 |
| EPI_ISL_944964 | EPI_ISL_945635 | EPI_ISL_945877 | EPI_ISL_946127 | EPI_ISL_949579 | EPI_ISL_952991 | EPI_ISL_956533 |
| EPI_ISL_944967 | EPI_ISL_945636 | EPI_ISL_945878 | EPI_ISL_946135 | EPI_ISL_949580 | EPI_ISL_953003 | EPI_ISL_956534 |
| EPI_ISL_944977 | EPI_ISL_945648 | EPI_ISL_945879 | EPI_ISL_946145 | EPI_ISL_949581 | EPI_ISL_953006 | EPI_ISL_956536 |
| EPI_ISL_944980 | EPI_ISL_945650 | EPI_ISL_945882 | EPI_ISL_946147 | EPI_ISL_949583 | EPI_ISL_953017 | EPI_ISL_956538 |
| EPI_ISL_944982 | EPI_ISL_945656 | EPI_ISL_945885 | EPI_ISL_946150 | EPI_ISL_949584 | EPI_ISL_953240 | EPI_ISL_956540 |
| EPI_ISL_944983 | EPI_ISL_945664 | EPI_ISL_945889 | EPI_ISL_946157 | EPI_ISL_949585 | EPI_ISL_953249 | EPI_ISL_956542 |
| EPI_ISL_944984 | EPI_ISL_945668 | EPI_ISL_945895 | EPI_ISL_946160 | EPI_ISL_949586 | EPI_ISL_953250 | EPI_ISL_956544 |
| EPI_ISL_944987 | EPI_ISL_945672 | EPI_ISL_945898 | EPI_ISL_946162 | EPI_ISL_949587 | EPI_ISL_953252 | EPI_ISL_956545 |
| EPI_ISL_944988 | EPI_ISL_945674 | EPI_ISL_945899 | EPI_ISL_946169 | EPI_ISL_949593 | EPI_ISL_953255 | EPI_ISL_956547 |
| EPI_ISL_944989 | EPI_ISL_945677 | EPI_ISL_945900 | EPI_ISL_946173 | EPI_ISL_949594 | EPI_ISL_953256 | EPI_ISL_956548 |
| EPI_ISL_944990 | EPI_ISL_945678 | EPI_ISL_945904 | EPI_ISL_946174 | EPI_ISL_949595 | EPI_ISL_953257 | EPI_ISL_956550 |
| EPI_ISL_944991 | EPI_ISL_945681 | EPI_ISL_945906 | EPI_ISL_946176 | EPI_ISL_949599 | EPI_ISL_953258 | EPI_ISL_956551 |
| EPI_ISL_944992 | EPI_ISL_945682 | EPI_ISL_945907 | EPI_ISL_946177 | EPI_ISL_949600 | EPI_ISL_953264 | EPI_ISL_956552 |
| EPI_ISL_944993 | EPI_ISL_945692 | EPI_ISL_945908 | EPI_ISL_946180 | EPI_ISL_949602 | EPI_ISL_953265 | EPI_ISL_956553 |
| EPI_ISL_944994 | EPI_ISL_945696 | EPI_ISL_945909 | EPI_ISL_946181 | EPI_ISL_949616 | EPI_ISL_953269 | EPI_ISL_956554 |
| EPI_ISL_944995 | EPI_ISL_945697 | EPI_ISL_945910 | EPI_ISL_946182 | EPI_ISL_949617 | EPI_ISL_953270 | EPI_ISL_956555 |
| EPI_ISL_945000 | EPI_ISL_945698 | EPI_ISL_945914 | EPI_ISL_946188 | EPI_ISL_949619 | EPI_ISL_953271 | EPI_ISL_956557 |
| EPI_ISL_945001 | EPI_ISL_945704 | EPI_ISL_945920 | EPI_ISL_946190 | EPI_ISL_949623 | EPI_ISL_953282 | EPI_ISL_956558 |
| EPI_ISL_945003 | EPI_ISL_945718 | EPI_ISL_945921 | EPI_ISL_946192 | EPI_ISL_949662 | EPI_ISL_956422 | EPI_ISL_956560 |
| EPI_ISL_945005 | EPI_ISL_945722 | EPI_ISL_945926 | EPI_ISL_946196 | EPI_ISL_949664 | EPI_ISL_956423 | EPI_ISL_956561 |
| EPI_ISL_945006 | EPI_ISL_945726 | EPI_ISL_945933 | EPI_ISL_946202 | EPI_ISL_949666 | EPI_ISL_956424 | EPI_ISL_956563 |
| EPI_ISL_945008 | EPI_ISL_945728 | EPI_ISL_945938 | EPI_ISL_946206 | EPI_ISL_949668 | EPI_ISL_956425 | EPI_ISL_956564 |

- USA

| EPI_ISL_911372 | EPI_ISL_1014808 | EPI_ISL_1067807 | EPI_ISL_1158368 | EPI_ISL_1194419 | EPI_ISL_1379270 | EPI_ISL_1814034 |
| --- | --- | --- | --- | --- | --- | --- |
| EPI_ISL_911378 | EPI_ISL_1014809 | EPI_ISL_1069328 | EPI_ISL_1158369 | EPI_ISL_1194733 | EPI_ISL_1379271 | EPI_ISL_1814102 |
| EPI_ISL_942354 | EPI_ISL_1015565 | EPI_ISL_1080155 | EPI_ISL_1158372 | EPI_ISL_1195948 | EPI_ISL_1379272 | EPI_ISL_1814181 |
| EPI_ISL_955111 | EPI_ISL_1015567 | EPI_ISL_1080159 | EPI_ISL_1158373 | EPI_ISL_1195949 | EPI_ISL_1379273 | EPI_ISL_1814352 |
| EPI_ISL_955218 | EPI_ISL_1016013 | EPI_ISL_1080170 | EPI_ISL_1158374 | EPI_ISL_1196877 | EPI_ISL_1379275 | EPI_ISL_1814621 |
| EPI_ISL_955290 | EPI_ISL_1016014 | EPI_ISL_1080175 | EPI_ISL_1158375 | EPI_ISL_1196878 | EPI_ISL_1379276 | EPI_ISL_1814703 |
| EPI_ISL_955292 | EPI_ISL_1016015 | EPI_ISL_1080217 | EPI_ISL_1158520 | EPI_ISL_1201881 | EPI_ISL_1379278 | EPI_ISL_1814786 |
| EPI_ISL_955308 | EPI_ISL_1016075 | EPI_ISL_1080231 | EPI_ISL_1158523 | EPI_ISL_1203834 | EPI_ISL_1379280 | EPI_ISL_1814970 |
| EPI_ISL_955906 | EPI_ISL_1016169 | EPI_ISL_1080255 | EPI_ISL_1163732 | EPI_ISL_1205289 | EPI_ISL_1379285 | EPI_ISL_1815123 |
| EPI_ISL_955909 | EPI_ISL_1016175 | EPI_ISL_1080321 | EPI_ISL_1167297 | EPI_ISL_1224771 | EPI_ISL_1379289 | EPI_ISL_1815571 |
| EPI_ISL_955925 | EPI_ISL_1016176 | EPI_ISL_1080337 | EPI_ISL_1168724 | EPI_ISL_1224822 | EPI_ISL_1379292 | EPI_ISL_1815934 |
| EPI_ISL_955926 | EPI_ISL_1016198 | EPI_ISL_1081045 | EPI_ISL_1168727 | EPI_ISL_1224914 | EPI_ISL_1379293 | EPI_ISL_1816195 |
| EPI_ISL_955927 | EPI_ISL_1016253 | EPI_ISL_1081046 | EPI_ISL_1168746 | EPI_ISL_1224916 | EPI_ISL_1379298 | EPI_ISL_1816305 |
| EPI_ISL_955937 | EPI_ISL_1016693 | EPI_ISL_1081047 | EPI_ISL_1168747 | EPI_ISL_1225013 | EPI_ISL_1379305 | EPI_ISL_1816625 |
| EPI_ISL_962458 | EPI_ISL_1016694 | EPI_ISL_1081048 | EPI_ISL_1168748 | EPI_ISL_1225016 | EPI_ISL_1379310 | EPI_ISL_1816663 |
| EPI_ISL_965155 | EPI_ISL_1016696 | EPI_ISL_1081050 | EPI_ISL_1168749 | EPI_ISL_1225019 | EPI_ISL_1379311 | EPI_ISL_1824749 |
| EPI_ISL_965156 | EPI_ISL_1016697 | EPI_ISL_1081052 | EPI_ISL_1168750 | EPI_ISL_1225020 | EPI_ISL_1379314 | EPI_ISL_1910988 |
| EPI_ISL_966310 | EPI_ISL_1016698 | EPI_ISL_1081053 | EPI_ISL_1168751 | EPI_ISL_1225640 | EPI_ISL_1379315 | EPI_ISL_1912833 |
| EPI_ISL_966397 | EPI_ISL_1016699 | EPI_ISL_1081054 | EPI_ISL_1168752 | EPI_ISL_1225654 | EPI_ISL_1379316 | EPI_ISL_2008040 |
| EPI_ISL_967770 | EPI_ISL_1016700 | EPI_ISL_1081055 | EPI_ISL_1168753 | EPI_ISL_1226605 | EPI_ISL_1379317 | EPI_ISL_2102563 |
| EPI_ISL_979457 | EPI_ISL_1016701 | EPI_ISL_1081056 | EPI_ISL_1168754 | EPI_ISL_1233309 | EPI_ISL_1379318 | EPI_ISL_2112525 |
| EPI_ISL_981082 | EPI_ISL_1016702 | EPI_ISL_1081057 | EPI_ISL_1168755 | EPI_ISL_1233439 | EPI_ISL_1379329 | EPI_ISL_2157607 |
| EPI_ISL_981119 | EPI_ISL_1016703 | EPI_ISL_1081058 | EPI_ISL_1168756 | EPI_ISL_1233871 | EPI_ISL_1379333 | EPI_ISL_2157608 |
| EPI_ISL_983374 | EPI_ISL_1016704 | EPI_ISL_1081059 | EPI_ISL_1168757 | EPI_ISL_1235057 | EPI_ISL_1379334 | EPI_ISL_2157609 |
| EPI_ISL_983653 | EPI_ISL_1016705 | EPI_ISL_1081060 | EPI_ISL_1168758 | EPI_ISL_1235701 | EPI_ISL_1394608 | EPI_ISL_2157610 |
| EPI_ISL_983654 | EPI_ISL_1016706 | EPI_ISL_1081061 | EPI_ISL_1168759 | EPI_ISL_1235755 | EPI_ISL_1394613 | EPI_ISL_2157611 |
| EPI_ISL_983655 | EPI_ISL_1016707 | EPI_ISL_1081062 | EPI_ISL_1168760 | EPI_ISL_1235770 | EPI_ISL_1394628 | EPI_ISL_2157612 |
| EPI_ISL_983656 | EPI_ISL_1016708 | EPI_ISL_1081064 | EPI_ISL_1168761 | EPI_ISL_1235783 | EPI_ISL_1400806 | EPI_ISL_2283202 |
| EPI_ISL_983660 | EPI_ISL_1016709 | EPI_ISL_1081066 | EPI_ISL_1169490 | EPI_ISL_1235837 | EPI_ISL_1405664 | EPI_ISL_2340663 |
| EPI_ISL_983665 | EPI_ISL_1016710 | EPI_ISL_1081067 | EPI_ISL_1182027 | EPI_ISL_1235869 | EPI_ISL_1443812 | EPI_ISL_2379258 |
| EPI_ISL_983666 | EPI_ISL_1016711 | EPI_ISL_1081068 | EPI_ISL_1182028 | EPI_ISL_1239920 | EPI_ISL_1446985 | EPI_ISL_2427503 |
| EPI_ISL_983667 | EPI_ISL_1016712 | EPI_ISL_1081069 | EPI_ISL_1193261 | EPI_ISL_1239921 | EPI_ISL_1447054 | EPI_ISL_2458173 |
| EPI_ISL_983668 | EPI_ISL_1016713 | EPI_ISL_1081070 | EPI_ISL_1193262 | EPI_ISL_1239922 | EPI_ISL_1483795 | EPI_ISL_2603364 |
| EPI_ISL_983669 | EPI_ISL_1016714 | EPI_ISL_1081071 | EPI_ISL_1193263 | EPI_ISL_1239923 | EPI_ISL_1501983 | EPI_ISL_2634210 |
| EPI_ISL_983670 | EPI_ISL_1016715 | EPI_ISL_1081072 | EPI_ISL_1193264 | EPI_ISL_1239924 | EPI_ISL_1503561 | EPI_ISL_2715598 |
| EPI_ISL_983671 | EPI_ISL_1016716 | EPI_ISL_1081073 | EPI_ISL_1193265 | EPI_ISL_1239925 | EPI_ISL_1503562 | EPI_ISL_2715793 |
| EPI_ISL_983678 | EPI_ISL_1016717 | EPI_ISL_1081074 | EPI_ISL_1193266 | EPI_ISL_1239926 | EPI_ISL_1509046 | EPI_ISL_2715809 |
| EPI_ISL_983679 | EPI_ISL_1016718 | EPI_ISL_1081075 | EPI_ISL_1193267 | EPI_ISL_1239927 | EPI_ISL_1522343 | EPI_ISL_2715811 |
| EPI_ISL_983680 | EPI_ISL_1016719 | EPI_ISL_1081076 | EPI_ISL_1193268 | EPI_ISL_1239928 | EPI_ISL_1526912 | EPI_ISL_2715826 |
| EPI_ISL_983708 | EPI_ISL_1016720 | EPI_ISL_1081077 | EPI_ISL_1193269 | EPI_ISL_1239929 | EPI_ISL_1584484 | EPI_ISL_2715918 |
| EPI_ISL_983709 | EPI_ISL_1016721 | EPI_ISL_1081078 | EPI_ISL_1193270 | EPI_ISL_1239940 | EPI_ISL_1592261 | EPI_ISL_2715924 |
| EPI_ISL_983714 | EPI_ISL_1016722 | EPI_ISL_1081079 | EPI_ISL_1193272 | EPI_ISL_1239954 | EPI_ISL_1592273 | EPI_ISL_2715937 |
| EPI_ISL_983721 | EPI_ISL_1016786 | EPI_ISL_1081080 | EPI_ISL_1193273 | EPI_ISL_1239968 | EPI_ISL_1592277 | EPI_ISL_2715960 |
| EPI_ISL_983722 | EPI_ISL_1016789 | EPI_ISL_1081081 | EPI_ISL_1193275 | EPI_ISL_1239970 | EPI_ISL_1592300 | EPI_ISL_2715966 |
| EPI_ISL_983723 | EPI_ISL_1016790 | EPI_ISL_1081082 | EPI_ISL_1193276 | EPI_ISL_1278020 | EPI_ISL_1592302 | EPI_ISL_2715999 |
| EPI_ISL_983724 | EPI_ISL_1016791 | EPI_ISL_1081083 | EPI_ISL_1193277 | EPI_ISL_1291310 | EPI_ISL_1592308 | EPI_ISL_2716128 |
| EPI_ISL_983725 | EPI_ISL_1016792 | EPI_ISL_1081084 | EPI_ISL_1193278 | EPI_ISL_1291318 | EPI_ISL_1592317 | EPI_ISL_2716172 |
| EPI_ISL_983732 | EPI_ISL_1016793 | EPI_ISL_1081085 | EPI_ISL_1193279 | EPI_ISL_1291322 | EPI_ISL_1592318 | EPI_ISL_2716199 |
| EPI_ISL_984189 | EPI_ISL_1016821 | EPI_ISL_1081086 | EPI_ISL_1193280 | EPI_ISL_1291327 | EPI_ISL_1592334 | EPI_ISL_2759471 |
| EPI_ISL_984241 | EPI_ISL_1017989 | EPI_ISL_1081087 | EPI_ISL_1193281 | EPI_ISL_1291336 | EPI_ISL_1592364 | EPI_ISL_2759637 |
| EPI_ISL_984298 | EPI_ISL_1018057 | EPI_ISL_1081088 | EPI_ISL_1193282 | EPI_ISL_1291337 | EPI_ISL_1592369 | EPI_ISL_2778248 |
| EPI_ISL_984299 | EPI_ISL_1032991 | EPI_ISL_1081089 | EPI_ISL_1193284 | EPI_ISL_1291339 | EPI_ISL_1592395 | EPI_ISL_2869057 |
| EPI_ISL_984753 | EPI_ISL_1034279 | EPI_ISL_1081090 | EPI_ISL_1193286 | EPI_ISL_1291340 | EPI_ISL_1620647 | EPI_ISL_2876494 |
| EPI_ISL_984754 | EPI_ISL_1035846 | EPI_ISL_1081091 | EPI_ISL_1193287 | EPI_ISL_1291357 | EPI_ISL_1620650 | EPI_ISL_2920401 |
| EPI_ISL_984919 | EPI_ISL_1035858 | EPI_ISL_1081092 | EPI_ISL_1193288 | EPI_ISL_1291364 | EPI_ISL_1622889 | EPI_ISL_2920410 |
| EPI_ISL_984921 | EPI_ISL_1036686 | EPI_ISL_1081093 | EPI_ISL_1193289 | EPI_ISL_1291366 | EPI_ISL_1622893 | EPI_ISL_2920413 |
| EPI_ISL_984948 | EPI_ISL_1036688 | EPI_ISL_1081094 | EPI_ISL_1193290 | EPI_ISL_1291371 | EPI_ISL_1622895 | EPI_ISL_2920423 |
| EPI_ISL_985236 | EPI_ISL_1036689 | EPI_ISL_1081095 | EPI_ISL_1193291 | EPI_ISL_1291372 | EPI_ISL_1622896 | EPI_ISL_3021482 |
| EPI_ISL_985253 | EPI_ISL_1036690 | EPI_ISL_1081096 | EPI_ISL_1193292 | EPI_ISL_1291374 | EPI_ISL_1622897 | EPI_ISL_3022390 |
| EPI_ISL_985286 | EPI_ISL_1036691 | EPI_ISL_1081266 | EPI_ISL_1193293 | EPI_ISL_1291375 | EPI_ISL_1624211 | EPI_ISL_3215838 |
| EPI_ISL_994831 | EPI_ISL_1036692 | EPI_ISL_1085166 | EPI_ISL_1193294 | EPI_ISL_1291376 | EPI_ISL_1624212 | EPI_ISL_3215846 |
| EPI_ISL_994836 | EPI_ISL_1036693 | EPI_ISL_1091896 | EPI_ISL_1193295 | EPI_ISL_1291833 | EPI_ISL_1624213 | EPI_ISL_3246235 |
| EPI_ISL_994857 | EPI_ISL_1036720 | EPI_ISL_1091973 | EPI_ISL_1194379 | EPI_ISL_1295202 | EPI_ISL_1626882 | EPI_ISL_3246236 |
| EPI_ISL_994875 | EPI_ISL_1036721 | EPI_ISL_1097518 | EPI_ISL_1194380 | EPI_ISL_1295840 | EPI_ISL_1626962 | EPI_ISL_3353953 |
| EPI_ISL_994882 | EPI_ISL_1036722 | EPI_ISL_1097519 | EPI_ISL_1194381 | EPI_ISL_1300344 | EPI_ISL_1626965 | EPI_ISL_3353965 |
| EPI_ISL_994889 | EPI_ISL_1036723 | EPI_ISL_1097520 | EPI_ISL_1194382 | EPI_ISL_1300353 | EPI_ISL_1626968 | EPI_ISL_3354010 |
| EPI_ISL_994897 | EPI_ISL_1038985 | EPI_ISL_1097521 | EPI_ISL_1194383 | EPI_ISL_1300356 | EPI_ISL_1626970 | EPI_ISL_3354029 |
| EPI_ISL_994902 | EPI_ISL_1038987 | EPI_ISL_1097522 | EPI_ISL_1194384 | EPI_ISL_1300357 | EPI_ISL_1626971 | EPI_ISL_3354030 |
| EPI_ISL_1008851 | EPI_ISL_1039738 | EPI_ISL_1097523 | EPI_ISL_1194385 | EPI_ISL_1300367 | EPI_ISL_1626975 | EPI_ISL_3493633 |
| EPI_ISL_1008893 | EPI_ISL_1049205 | EPI_ISL_1110039 | EPI_ISL_1194386 | EPI_ISL_1300368 | EPI_ISL_1626981 | EPI_ISL_3504126 |
| EPI_ISL_1008895 | EPI_ISL_1049209 | EPI_ISL_1110040 | EPI_ISL_1194387 | EPI_ISL_1300371 | EPI_ISL_1626983 | EPI_ISL_3670661 |
| EPI_ISL_1008897 | EPI_ISL_1049213 | EPI_ISL_1114455 | EPI_ISL_1194388 | EPI_ISL_1300379 | EPI_ISL_1626987 | EPI_ISL_3670662 |
| EPI_ISL_1008901 | EPI_ISL_1049695 | EPI_ISL_1114457 | EPI_ISL_1194389 | EPI_ISL_1300385 | EPI_ISL_1708957 | EPI_ISL_3670663 |
| EPI_ISL_1008904 | EPI_ISL_1053433 | EPI_ISL_1114458 | EPI_ISL_1194390 | EPI_ISL_1300386 | EPI_ISL_1710710 | EPI_ISL_3670664 |
| EPI_ISL_1008905 | EPI_ISL_1053434 | EPI_ISL_1114459 | EPI_ISL_1194391 | EPI_ISL_1300387 | EPI_ISL_1710711 | EPI_ISL_3670665 |
| EPI_ISL_1008906 | EPI_ISL_1058780 | EPI_ISL_1121784 | EPI_ISL_1194392 | EPI_ISL_1300405 | EPI_ISL_1710712 | EPI_ISL_3670676 |
| EPI_ISL_1008915 | EPI_ISL_1058833 | EPI_ISL_1121805 | EPI_ISL_1194393 | EPI_ISL_1300804 | EPI_ISL_1710713 | EPI_ISL_3670790 |
| EPI_ISL_1008921 | EPI_ISL_1058841 | EPI_ISL_1132738 | EPI_ISL_1194394 | EPI_ISL_1303800 | EPI_ISL_1710719 | EPI_ISL_3670791 |
| EPI_ISL_1008923 | EPI_ISL_1058860 | EPI_ISL_1132741 | EPI_ISL_1194395 | EPI_ISL_1306229 | EPI_ISL_1710720 | EPI_ISL_3670792 |
| EPI_ISL_1009731 | EPI_ISL_1058951 | EPI_ISL_1132796 | EPI_ISL_1194396 | EPI_ISL_1321811 | EPI_ISL_1812895 | EPI_ISL_3670793 |
| EPI_ISL_1009733 | EPI_ISL_1060791 | EPI_ISL_1157404 | EPI_ISL_1194397 | EPI_ISL_1321820 | EPI_ISL_1813060 | EPI_ISL_4096754 |
| EPI_ISL_1009734 | EPI_ISL_1064344 | EPI_ISL_1158358 | EPI_ISL_1194398 | EPI_ISL_1321821 | EPI_ISL_1813179 | EPI_ISL_4183212 |
| EPI_ISL_1009738 | EPI_ISL_1064371 | EPI_ISL_1158359 | EPI_ISL_1194399 | EPI_ISL_1321823 | EPI_ISL_1813272 | EPI_ISL_4261987 |
| EPI_ISL_1009749 | EPI_ISL_1064375 | EPI_ISL_1158360 | EPI_ISL_1194400 | EPI_ISL_1321824 | EPI_ISL_1813455 | EPI_ISL_4961477 |
| EPI_ISL_1009827 | EPI_ISL_1064386 | EPI_ISL_1158361 | EPI_ISL_1194401 | EPI_ISL_1323431 | EPI_ISL_1813519 | EPI_ISL_4961561 |
| EPI_ISL_1009957 | EPI_ISL_1064387 | EPI_ISL_1158363 | EPI_ISL_1194402 | EPI_ISL_1358957 | EPI_ISL_1813672 | EPI_ISL_4961795 |
| EPI_ISL_1010524 | EPI_ISL_1064778 | EPI_ISL_1158364 | EPI_ISL_1194404 | EPI_ISL_1364519 | EPI_ISL_1813816 | EPI_ISL_4962015 |
| EPI_ISL_1011681 | EPI_ISL_1067761 | EPI_ISL_1158365 | EPI_ISL_1194406 | EPI_ISL_1373845 | EPI_ISL_1813959 | EPI_ISL_4962036 |
| EPI_ISL_1014767 | EPI_ISL_1067788 | EPI_ISL_1158366 | EPI_ISL_1194409 | EPI_ISL_1373857 | EPI_ISL_1814013 | EPI_ISL_4962049 |
| EPI_ISL_1014807 | EPI_ISL_1067791 | EPI_ISL_1158367 | EPI_ISL_1194412 | EPI_ISL_1373868 | EPI_ISL_1814023 | EPI_ISL_4962053 |
| EPI_ISL_4962141 | EPI_ISL_5863382 | EPI_ISL_12312423 | EPI_ISL_12978440 | EPI_ISL_13678142 | EPI_ISL_7703869 | EPI_ISL_12977625 |
| EPI_ISL_4962389 | EPI_ISL_5863383 | EPI_ISL_12975751 | EPI_ISL_12978835 | EPI_ISL_14793799 | EPI_ISL_9158418 | EPI_ISL_12977716 |
| EPI_ISL_4962409 | EPI_ISL_6566190 | EPI_ISL_12976209 | EPI_ISL_12978864 | EPI_ISL_15033023 | EPI_ISL_12175982 | EPI_ISL_12978027 |
| EPI_ISL_4962422 | EPI_ISL_6566191 | EPI_ISL_12977220 | EPI_ISL_12978886 | EPI_ISL_17332576 | EPI_ISL_12979026 | EPI_ISL_12978155 |
| EPI_ISL_4962613 | EPI_ISL_6566193 | EPI_ISL_12977371 | EPI_ISL_12978887 | EPI_ISL_17332577 | EPI_ISL_13595651 | EPI_ISL_12978350 |
| EPI_ISL_5703095 | EPI_ISL_6582194 | EPI_ISL_12977448 | EPI_ISL_12978890 | EPI_ISL_17577657 | EPI_ISL_13675357 | EPI_ISL_12978398 |
| EPI_ISL_5703299 | EPI_ISL_6582563 | EPI_ISL_12977509 | EPI_ISL_12979014 | EPI_ISL_17577669 | EPI_ISL_13678132 | EPI_ISL_5703323 |

- Australia

| EPI_ISL_933799 | EPI_ISL_1295936 | EPI_ISL_1633351 | EPI_ISL_1913204 | EPI_ISL_2650459 | EPI_ISL_3568431 | EPI_ISL_4254501 |
| --- | --- | --- | --- | --- | --- | --- |
| EPI_ISL_933801 | EPI_ISL_1306127 | EPI_ISL_1633352 | EPI_ISL_1913212 | EPI_ISL_2650461 | EPI_ISL_3568432 | EPI_ISL_4254502 |
| EPI_ISL_962180 | EPI_ISL_1306129 | EPI_ISL_1633659 | EPI_ISL_2001058 | EPI_ISL_2650462 | EPI_ISL_3568434 | EPI_ISL_4254504 |
| EPI_ISL_968212 | EPI_ISL_1306130 | EPI_ISL_1633675 | EPI_ISL_2107442 | EPI_ISL_2650463 | EPI_ISL_3568435 | EPI_ISL_4254505 |
| EPI_ISL_979356 | EPI_ISL_1306132 | EPI_ISL_1633677 | EPI_ISL_2107445 | EPI_ISL_2661515 | EPI_ISL_3568436 | EPI_ISL_4254507 |
| EPI_ISL_979357 | EPI_ISL_1315071 | EPI_ISL_1633678 | EPI_ISL_2107449 | EPI_ISL_2661517 | EPI_ISL_3568437 | EPI_ISL_4254513 |
| EPI_ISL_979359 | EPI_ISL_1340871 | EPI_ISL_1633679 | EPI_ISL_2107450 | EPI_ISL_2661521 | EPI_ISL_3568438 | EPI_ISL_4303406 |
| EPI_ISL_979360 | EPI_ISL_1340873 | EPI_ISL_1633682 | EPI_ISL_2107452 | EPI_ISL_2661524 | EPI_ISL_3568443 | EPI_ISL_5200556 |
| EPI_ISL_979361 | EPI_ISL_1340878 | EPI_ISL_1633683 | EPI_ISL_2107453 | EPI_ISL_2661525 | EPI_ISL_3568445 | EPI_ISL_5288649 |
| EPI_ISL_979362 | EPI_ISL_1341507 | EPI_ISL_1660399 | EPI_ISL_2107457 | EPI_ISL_2675252 | EPI_ISL_3568453 | EPI_ISL_5288704 |
| EPI_ISL_979363 | EPI_ISL_1364538 | EPI_ISL_1660401 | EPI_ISL_2107459 | EPI_ISL_2675253 | EPI_ISL_3568509 | EPI_ISL_5288773 |
| EPI_ISL_979364 | EPI_ISL_1364540 | EPI_ISL_1660402 | EPI_ISL_2107502 | EPI_ISL_2675254 | EPI_ISL_3568548 | EPI_ISL_5288779 |
| EPI_ISL_985235 | EPI_ISL_1364541 | EPI_ISL_1660403 | EPI_ISL_2107503 | EPI_ISL_2675255 | EPI_ISL_3568550 | EPI_ISL_5288788 |
| EPI_ISL_1010703 | EPI_ISL_1366740 | EPI_ISL_1660409 | EPI_ISL_2107504 | EPI_ISL_2695786 | EPI_ISL_3614817 | EPI_ISL_5288795 |
| EPI_ISL_1010712 | EPI_ISL_1383237 | EPI_ISL_1660410 | EPI_ISL_2107505 | EPI_ISL_2695787 | EPI_ISL_3614826 | EPI_ISL_5288806 |
| EPI_ISL_1010713 | EPI_ISL_1383240 | EPI_ISL_1660411 | EPI_ISL_2107530 | EPI_ISL_2695788 | EPI_ISL_3614831 | EPI_ISL_5288813 |
| EPI_ISL_1010714 | EPI_ISL_1383241 | EPI_ISL_1660476 | EPI_ISL_2107531 | EPI_ISL_2695789 | EPI_ISL_4254499 | EPI_ISL_5288821 |
| EPI_ISL_1010715 | EPI_ISL_1383243 | EPI_ISL_1660477 | EPI_ISL_2107532 | EPI_ISL_2696273 | EPI_ISL_4254500 | EPI_ISL_5288827 |
| EPI_ISL_1033027 | EPI_ISL_1383244 | EPI_ISL_1660478 | EPI_ISL_2107533 | EPI_ISL_2727637 | EPI_ISL_2462366 | EPI_ISL_5288834 |
| EPI_ISL_1033145 | EPI_ISL_1383245 | EPI_ISL_1669116 | EPI_ISL_2107534 | EPI_ISL_2811889 | EPI_ISL_2462408 | EPI_ISL_5288840 |
| EPI_ISL_1033146 | EPI_ISL_1383246 | EPI_ISL_1669120 | EPI_ISL_2226229 | EPI_ISL_2811892 | EPI_ISL_2462412 | EPI_ISL_5288848 |
| EPI_ISL_1033147 | EPI_ISL_1384811 | EPI_ISL_1669123 | EPI_ISL_2249258 | EPI_ISL_2811893 | EPI_ISL_2462413 | EPI_ISL_5288853 |
| EPI_ISL_1033148 | EPI_ISL_1396519 | EPI_ISL_1669124 | EPI_ISL_2250186 | EPI_ISL_2811895 | EPI_ISL_2462419 | EPI_ISL_5288861 |
| EPI_ISL_1033149 | EPI_ISL_1396522 | EPI_ISL_1669126 | EPI_ISL_2250187 | EPI_ISL_2811896 | EPI_ISL_2462420 | EPI_ISL_5288868 |
| EPI_ISL_1033150 | EPI_ISL_1396525 | EPI_ISL_1672850 | EPI_ISL_2250194 | EPI_ISL_2811899 | EPI_ISL_2462422 | EPI_ISL_5288876 |
| EPI_ISL_1033151 | EPI_ISL_1396530 | EPI_ISL_1704673 | EPI_ISL_2250205 | EPI_ISL_2811900 | EPI_ISL_2462424 | EPI_ISL_5288885 |
| EPI_ISL_1033152 | EPI_ISL_1396531 | EPI_ISL_1704675 | EPI_ISL_2250215 | EPI_ISL_2811902 | EPI_ISL_2462426 | EPI_ISL_5288889 |
| EPI_ISL_1033153 | EPI_ISL_1396532 | EPI_ISL_1704676 | EPI_ISL_2250222 | EPI_ISL_2811904 | EPI_ISL_2462427 | EPI_ISL_5288896 |
| EPI_ISL_1033154 | EPI_ISL_1396533 | EPI_ISL_1704677 | EPI_ISL_2250223 | EPI_ISL_2811905 | EPI_ISL_2462428 | EPI_ISL_5288905 |
| EPI_ISL_1033155 | EPI_ISL_1397953 | EPI_ISL_1704678 | EPI_ISL_2250224 | EPI_ISL_2811906 | EPI_ISL_2462429 | EPI_ISL_5288912 |
| EPI_ISL_1033156 | EPI_ISL_1397954 | EPI_ISL_1704787 | EPI_ISL_2250225 | EPI_ISL_2839571 | EPI_ISL_2462430 | EPI_ISL_5288919 |
| EPI_ISL_1033157 | EPI_ISL_1397955 | EPI_ISL_1704788 | EPI_ISL_2250226 | EPI_ISL_2842837 | EPI_ISL_2462431 | EPI_ISL_5288924 |
| EPI_ISL_1061033 | EPI_ISL_1397956 | EPI_ISL_1704789 | EPI_ISL_2250228 | EPI_ISL_2842838 | EPI_ISL_2534585 | EPI_ISL_5288932 |
| EPI_ISL_1061036 | EPI_ISL_1397957 | EPI_ISL_1704790 | EPI_ISL_2250229 | EPI_ISL_2842841 | EPI_ISL_2603822 | EPI_ISL_5288941 |
| EPI_ISL_1061403 | EPI_ISL_1404915 | EPI_ISL_1704791 | EPI_ISL_2250231 | EPI_ISL_2842842 | EPI_ISL_2603823 | EPI_ISL_5288950 |
| EPI_ISL_1069392 | EPI_ISL_1404916 | EPI_ISL_1704792 | EPI_ISL_2250232 | EPI_ISL_2842843 | EPI_ISL_2629000 | EPI_ISL_5288955 |
| EPI_ISL_1069393 | EPI_ISL_1404917 | EPI_ISL_1704793 | EPI_ISL_2250233 | EPI_ISL_2842844 | EPI_ISL_2629001 | EPI_ISL_5314294 |
| EPI_ISL_1098798 | EPI_ISL_1404918 | EPI_ISL_1704795 | EPI_ISL_2274247 | EPI_ISL_2842846 | EPI_ISL_2650451 | EPI_ISL_5314306 |
| EPI_ISL_1098799 | EPI_ISL_1404919 | EPI_ISL_1704796 | EPI_ISL_2321158 | EPI_ISL_2896242 | EPI_ISL_2650452 | EPI_ISL_5314308 |
| EPI_ISL_1121977 | EPI_ISL_1404920 | EPI_ISL_1704797 | EPI_ISL_2321163 | EPI_ISL_2896243 | EPI_ISL_2650453 | EPI_ISL_5314310 |
| EPI_ISL_1121978 | EPI_ISL_1404921 | EPI_ISL_1704799 | EPI_ISL_2321167 | EPI_ISL_2907548 | EPI_ISL_1904446 | EPI_ISL_5314317 |
| EPI_ISL_1121979 | EPI_ISL_1404922 | EPI_ISL_1704800 | EPI_ISL_2321173 | EPI_ISL_2920927 | EPI_ISL_1904447 | EPI_ISL_5314319 |
| EPI_ISL_1121984 | EPI_ISL_1416321 | EPI_ISL_1704801 | EPI_ISL_2322898 | EPI_ISL_2920928 | EPI_ISL_1904448 | EPI_ISL_5314373 |
| EPI_ISL_1121985 | EPI_ISL_1424002 | EPI_ISL_1704802 | EPI_ISL_2379273 | EPI_ISL_2920930 | EPI_ISL_1904450 | EPI_ISL_5314398 |
| EPI_ISL_1121986 | EPI_ISL_1424003 | EPI_ISL_1704803 | EPI_ISL_2402460 | EPI_ISL_2920931 | EPI_ISL_1904451 | EPI_ISL_5314406 |
| EPI_ISL_1121988 | EPI_ISL_1424004 | EPI_ISL_1704804 | EPI_ISL_2404987 | EPI_ISL_2920935 | EPI_ISL_1904452 | EPI_ISL_5314411 |
| EPI_ISL_1159376 | EPI_ISL_1424005 | EPI_ISL_1704806 | EPI_ISL_2404988 | EPI_ISL_2920937 | EPI_ISL_1904453 | EPI_ISL_5314417 |
| EPI_ISL_1170949 | EPI_ISL_1424504 | EPI_ISL_1704807 | EPI_ISL_2404989 | EPI_ISL_2920944 | EPI_ISL_1904456 | EPI_ISL_5314437 |
| EPI_ISL_1170953 | EPI_ISL_1424507 | EPI_ISL_1704808 | EPI_ISL_2404992 | EPI_ISL_2920955 | EPI_ISL_1904461 | EPI_ISL_5314444 |
| EPI_ISL_1170954 | EPI_ISL_1447288 | EPI_ISL_1706364 | EPI_ISL_2455225 | EPI_ISL_2920963 | EPI_ISL_1904471 | EPI_ISL_5314449 |
| EPI_ISL_1184094 | EPI_ISL_1447289 | EPI_ISL_1706367 | EPI_ISL_2455226 | EPI_ISL_2920986 | EPI_ISL_1910857 | EPI_ISL_5314455 |
| EPI_ISL_1184095 | EPI_ISL_1447290 | EPI_ISL_1716515 | EPI_ISL_2455228 | EPI_ISL_2920996 | EPI_ISL_1911180 | EPI_ISL_5314469 |
| EPI_ISL_1184096 | EPI_ISL_1448429 | EPI_ISL_1716518 | EPI_ISL_2455230 | EPI_ISL_2920997 | EPI_ISL_1911181 | EPI_ISL_5656411 |
| EPI_ISL_1184097 | EPI_ISL_1465880 | EPI_ISL_1750963 | EPI_ISL_2462337 | EPI_ISL_2920998 | EPI_ISL_1911184 | EPI_ISL_5656414 |
| EPI_ISL_1184098 | EPI_ISL_1465883 | EPI_ISL_1750966 | EPI_ISL_2462338 | EPI_ISL_2920999 | EPI_ISL_1911186 | EPI_ISL_5656416 |
| EPI_ISL_1184504 | EPI_ISL_1465886 | EPI_ISL_1750967 | EPI_ISL_2462339 | EPI_ISL_2956009 | EPI_ISL_1911188 | EPI_ISL_5656417 |
| EPI_ISL_1184505 | EPI_ISL_1465887 | EPI_ISL_1750968 | EPI_ISL_2462340 | EPI_ISL_2956012 | EPI_ISL_1913108 | EPI_ISL_5656418 |
| EPI_ISL_1184506 | EPI_ISL_1465888 | EPI_ISL_1754862 | EPI_ISL_2462341 | EPI_ISL_3011179 | EPI_ISL_1913175 | EPI_ISL_9285760 |
| EPI_ISL_1196417 | EPI_ISL_1465889 | EPI_ISL_1754864 | EPI_ISL_2462347 | EPI_ISL_3072004 | EPI_ISL_1913200 | EPI_ISL_9285761 |
| EPI_ISL_1196418 | EPI_ISL_1465890 | EPI_ISL_1754865 | EPI_ISL_2462350 | EPI_ISL_3161813 | EPI_ISL_1913201 | EPI_ISL_9285762 |
| EPI_ISL_1198800 | EPI_ISL_1465892 | EPI_ISL_1756021 | EPI_ISL_2462351 | EPI_ISL_3184935 | EPI_ISL_1913202 | EPI_ISL_9285767 |
| EPI_ISL_1198803 | EPI_ISL_1465893 | EPI_ISL_1756025 | EPI_ISL_2462354 | EPI_ISL_3200830 | EPI_ISL_1913203 | EPI_ISL_12275590 |
| EPI_ISL_1205303 | EPI_ISL_1465894 | EPI_ISL_1805698 | EPI_ISL_2462355 | EPI_ISL_3374204 | EPI_ISL_1543927 | EPI_ISL_17880831 |
| EPI_ISL_1222764 | EPI_ISL_1465896 | EPI_ISL_1805699 | EPI_ISL_2462356 | EPI_ISL_3417428 | EPI_ISL_1563679 | EPI_ISL_17880832 |
| EPI_ISL_1240991 | EPI_ISL_1483027 | EPI_ISL_1805700 | EPI_ISL_2462357 | EPI_ISL_3568425 | EPI_ISL_1563680 | EPI_ISL_17880835 |
| EPI_ISL_1240992 | EPI_ISL_1483028 | EPI_ISL_1816919 | EPI_ISL_2462360 | EPI_ISL_3568427 | EPI_ISL_1563681 | EPI_ISL_17880836 |
| EPI_ISL_1241836 | EPI_ISL_1483029 | EPI_ISL_1828699 | EPI_ISL_2462361 | EPI_ISL_3568429 | EPI_ISL_1615593 | EPI_ISL_17880837 |
| EPI_ISL_1241838 | EPI_ISL_1483031 | EPI_ISL_1904445 | EPI_ISL_2462362 | EPI_ISL_3568430 | EPI_ISL_1615594 | EPI_ISL_17880838 |
| EPI_ISL_1241839 | EPI_ISL_1483700 | EPI_ISL_1255310 | EPI_ISL_1293049 | EPI_ISL_1516884 | EPI_ISL_1620802 | EPI_ISL_1249993 |
| EPI_ISL_1241840 | EPI_ISL_1494718 | EPI_ISL_1273076 | EPI_ISL_1295930 | EPI_ISL_1516886 | EPI_ISL_1620803 | EPI_ISL_1249996 |
| EPI_ISL_1241841 | EPI_ISL_1494719 | EPI_ISL_1273083 | EPI_ISL_1295931 | EPI_ISL_1516889 | EPI_ISL_1620806 | EPI_ISL_1250002 |
| EPI_ISL_1241842 | EPI_ISL_1508992 | EPI_ISL_1293045 | EPI_ISL_1295933 | EPI_ISL_1543924 | EPI_ISL_1620807 | EPI_ISL_1250005 |
| EPI_ISL_1249986 | EPI_ISL_1516882 | EPI_ISL_1293048 | EPI_ISL_1295935 | EPI_ISL_1543925 | EPI_ISL_1633350 | EPI_ISL_1255309 |
| EPI_ISL_1249988 | EPI_ISL_1516883 | EPI_ISL_1249990 |  |  |  |  |

1. **Delta**

- China

| EPI_ISL_11873936 | EPI_ISL_15775727 | EPI_ISL_15775817 | EPI_ISL_13008956 | EPI_ISL_15775776 | EPI_ISL_15775866 | EPI_ISL_15775912 |
| --- | --- | --- | --- | --- | --- | --- |
| EPI_ISL_15756118 | EPI_ISL_15775728 | EPI_ISL_15775818 | EPI_ISL_13008957 | EPI_ISL_15775777 | EPI_ISL_15775867 | EPI_ISL_15775913 |
| EPI_ISL_5462229 | EPI_ISL_15775729 | EPI_ISL_15775819 | EPI_ISL_13008959 | EPI_ISL_15775778 | EPI_ISL_15775868 | EPI_ISL_15775914 |
| EPI_ISL_5462234 | EPI_ISL_15775730 | EPI_ISL_15775820 | EPI_ISL_13008961 | EPI_ISL_15775779 | EPI_ISL_15775869 | EPI_ISL_15775915 |
| EPI_ISL_5462235 | EPI_ISL_15775731 | EPI_ISL_15775821 | EPI_ISL_13008963 | EPI_ISL_15775780 | EPI_ISL_15775870 | EPI_ISL_15775916 |
| EPI_ISL_5462239 | EPI_ISL_15775732 | EPI_ISL_15775822 | EPI_ISL_13008964 | EPI_ISL_15775781 | EPI_ISL_15775871 | EPI_ISL_15775917 |
| EPI_ISL_5462260 | EPI_ISL_15775733 | EPI_ISL_15775823 | EPI_ISL_13008965 | EPI_ISL_15775782 | EPI_ISL_15775872 | EPI_ISL_15775918 |
| EPI_ISL_8582070 | EPI_ISL_15775734 | EPI_ISL_15775824 | EPI_ISL_13008967 | EPI_ISL_15775783 | EPI_ISL_15775873 | EPI_ISL_15775919 |
| EPI_ISL_11873903 | EPI_ISL_15775735 | EPI_ISL_15775825 | EPI_ISL_15756113 | EPI_ISL_15775784 | EPI_ISL_15775874 | EPI_ISL_15775920 |
| EPI_ISL_11873909 | EPI_ISL_15775736 | EPI_ISL_15775826 | EPI_ISL_15756122 | EPI_ISL_15775785 | EPI_ISL_15775875 | EPI_ISL_15775921 |
| EPI_ISL_13008894 | EPI_ISL_15775737 | EPI_ISL_15775827 | EPI_ISL_15756123 | EPI_ISL_15775786 | EPI_ISL_15775876 | EPI_ISL_15775922 |
| EPI_ISL_13008895 | EPI_ISL_15775738 | EPI_ISL_15775828 | EPI_ISL_15756988 | EPI_ISL_15775787 | EPI_ISL_15775877 | EPI_ISL_15775923 |
| EPI_ISL_13008896 | EPI_ISL_15775739 | EPI_ISL_15775829 | EPI_ISL_15756989 | EPI_ISL_15775788 | EPI_ISL_15775878 | EPI_ISL_15775924 |
| EPI_ISL_13008897 | EPI_ISL_15775740 | EPI_ISL_15775830 | EPI_ISL_15775697 | EPI_ISL_15775789 | EPI_ISL_15775879 | EPI_ISL_15775925 |
| EPI_ISL_13008898 | EPI_ISL_15775741 | EPI_ISL_15775831 | EPI_ISL_15775698 | EPI_ISL_15775790 | EPI_ISL_15775880 | EPI_ISL_15775926 |
| EPI_ISL_13008899 | EPI_ISL_15775742 | EPI_ISL_15775832 | EPI_ISL_15775699 | EPI_ISL_15775791 | EPI_ISL_15775881 | EPI_ISL_15775927 |
| EPI_ISL_13008900 | EPI_ISL_15775743 | EPI_ISL_15775833 | EPI_ISL_15775700 | EPI_ISL_15775792 | EPI_ISL_15775882 | EPI_ISL_15775928 |
| EPI_ISL_13008902 | EPI_ISL_15775744 | EPI_ISL_15775834 | EPI_ISL_15775701 | EPI_ISL_15775793 | EPI_ISL_15775883 | EPI_ISL_15775929 |
| EPI_ISL_13008905 | EPI_ISL_15775745 | EPI_ISL_15775835 | EPI_ISL_15775702 | EPI_ISL_15775794 | EPI_ISL_15775884 | EPI_ISL_15775930 |
| EPI_ISL_13008906 | EPI_ISL_15775746 | EPI_ISL_15775836 | EPI_ISL_15775703 | EPI_ISL_15775795 | EPI_ISL_15775885 | EPI_ISL_15775931 |
| EPI_ISL_13008907 | EPI_ISL_15775747 | EPI_ISL_15775837 | EPI_ISL_15775704 | EPI_ISL_15775796 | EPI_ISL_15775886 | EPI_ISL_15775932 |
| EPI_ISL_13008909 | EPI_ISL_15775748 | EPI_ISL_15775838 | EPI_ISL_15775705 | EPI_ISL_15775797 | EPI_ISL_15775887 | EPI_ISL_15775933 |
| EPI_ISL_13008910 | EPI_ISL_15775749 | EPI_ISL_15775839 | EPI_ISL_15775706 | EPI_ISL_15775798 | EPI_ISL_15775888 | EPI_ISL_15775934 |
| EPI_ISL_13008911 | EPI_ISL_15775750 | EPI_ISL_15775840 | EPI_ISL_15775707 | EPI_ISL_15775799 | EPI_ISL_15775889 | EPI_ISL_15775935 |
| EPI_ISL_13008912 | EPI_ISL_15775751 | EPI_ISL_15775841 | EPI_ISL_15775708 | EPI_ISL_15775800 | EPI_ISL_15775890 | EPI_ISL_15775936 |
| EPI_ISL_13008913 | EPI_ISL_15775752 | EPI_ISL_15775842 | EPI_ISL_15775709 | EPI_ISL_15775801 | EPI_ISL_15775891 | EPI_ISL_15783992 |
| EPI_ISL_13008914 | EPI_ISL_15775753 | EPI_ISL_15775843 | EPI_ISL_15775710 | EPI_ISL_15775802 | EPI_ISL_15775892 | EPI_ISL_15783993 |
| EPI_ISL_13008915 | EPI_ISL_15775754 | EPI_ISL_15775844 | EPI_ISL_15775711 | EPI_ISL_15775803 | EPI_ISL_15775893 | EPI_ISL_15783994 |
| EPI_ISL_13008916 | EPI_ISL_15775755 | EPI_ISL_15775845 | EPI_ISL_15775712 | EPI_ISL_15775804 | EPI_ISL_15775894 | EPI_ISL_15783995 |
| EPI_ISL_13008917 | EPI_ISL_15775756 | EPI_ISL_15775846 | EPI_ISL_15775713 | EPI_ISL_15775805 | EPI_ISL_15775895 | EPI_ISL_15783996 |
| EPI_ISL_13008918 | EPI_ISL_15775757 | EPI_ISL_15775847 | EPI_ISL_15775714 | EPI_ISL_15775806 | EPI_ISL_15775896 | EPI_ISL_15783997 |
| EPI_ISL_13008919 | EPI_ISL_15775758 | EPI_ISL_15775848 | EPI_ISL_15775715 | EPI_ISL_15775807 | EPI_ISL_15775897 | EPI_ISL_15783998 |
| EPI_ISL_13008925 | EPI_ISL_15775759 | EPI_ISL_15775849 | EPI_ISL_15775716 | EPI_ISL_15775808 | EPI_ISL_15775898 | EPI_ISL_15783999 |
| EPI_ISL_13008927 | EPI_ISL_15775760 | EPI_ISL_15775850 | EPI_ISL_15775717 | EPI_ISL_15775809 | EPI_ISL_15775899 | EPI_ISL_15784000 |
| EPI_ISL_13008928 | EPI_ISL_15775761 | EPI_ISL_15775851 | EPI_ISL_15775718 | EPI_ISL_15775810 | EPI_ISL_15775900 | EPI_ISL_15784001 |
| EPI_ISL_13008931 | EPI_ISL_15775762 | EPI_ISL_15775852 | EPI_ISL_15775719 | EPI_ISL_15775811 | EPI_ISL_15775901 | EPI_ISL_15784002 |
| EPI_ISL_13008935 | EPI_ISL_15775763 | EPI_ISL_15775853 | EPI_ISL_15775720 | EPI_ISL_15775812 | EPI_ISL_15775902 | EPI_ISL_15784003 |
| EPI_ISL_13008937 | EPI_ISL_15775764 | EPI_ISL_15775854 | EPI_ISL_15775721 | EPI_ISL_15775813 | EPI_ISL_15775903 | EPI_ISL_15784004 |
| EPI_ISL_13008938 | EPI_ISL_15775765 | EPI_ISL_15775855 | EPI_ISL_15775722 | EPI_ISL_15775814 | EPI_ISL_15775904 | EPI_ISL_15784005 |
| EPI_ISL_13008939 | EPI_ISL_15775766 | EPI_ISL_15775856 | EPI_ISL_15775723 | EPI_ISL_15775815 | EPI_ISL_15775905 | EPI_ISL_15784006 |
| EPI_ISL_13008940 | EPI_ISL_15775767 | EPI_ISL_15775857 | EPI_ISL_15775724 | EPI_ISL_15775816 | EPI_ISL_15775906 | EPI_ISL_15784007 |
| EPI_ISL_13008942 | EPI_ISL_15775768 | EPI_ISL_15775858 | EPI_ISL_15775725 | EPI_ISL_15775910 | EPI_ISL_15775863 | EPI_ISL_15784008 |
| EPI_ISL_13008943 | EPI_ISL_15775769 | EPI_ISL_15775859 | EPI_ISL_15775726 | EPI_ISL_15775911 | EPI_ISL_15775864 | EPI_ISL_15784009 |
| EPI_ISL_13008949 | EPI_ISL_15775770 | EPI_ISL_15775860 | EPI_ISL_13008954 | EPI_ISL_17261527 | EPI_ISL_15775865 | EPI_ISL_15784010 |
| EPI_ISL_13008951 | EPI_ISL_15775771 | EPI_ISL_15775861 | EPI_ISL_13008955 | EPI_ISL_17261530 | EPI_ISL_15775907 | EPI_ISL_15784011 |
| EPI_ISL_13008952 | EPI_ISL_15775772 | EPI_ISL_15775862 | EPI_ISL_15775775 | EPI_ISL_17261512 | EPI_ISL_15775908 | EPI_ISL_15784012 |
| EPI_ISL_13008953 | EPI_ISL_15775773 | EPI_ISL_15775774 | EPI_ISL_15775909 |  |  |  |

- Ghana

| EPI_ISL_4919697 | EPI_ISL_7661054 | EPI_ISL_8065769 | EPI_ISL_8065923 | EPI_ISL_10334572 | EPI_ISL_8065883 | EPI_ISL_10438347 |
| --- | --- | --- | --- | --- | --- | --- |
| EPI_ISL_4919698 | EPI_ISL_7661056 | EPI_ISL_8065770 | EPI_ISL_8065926 | EPI_ISL_10334584 | EPI_ISL_8065893 | EPI_ISL_11765401 |
| EPI_ISL_4919702 | EPI_ISL_7661078 | EPI_ISL_8065773 | EPI_ISL_8065927 | EPI_ISL_10334589 | EPI_ISL_8065894 | EPI_ISL_12692027 |
| EPI_ISL_4919703 | EPI_ISL_7661084 | EPI_ISL_8065782 | EPI_ISL_8065930 | EPI_ISL_10334590 | EPI_ISL_8065897 | EPI_ISL_12692046 |
| EPI_ISL_4919705 | EPI_ISL_7661093 | EPI_ISL_8065787 | EPI_ISL_8065934 | EPI_ISL_8065874 | EPI_ISL_8065898 | EPI_ISL_12692076 |
| EPI_ISL_4919708 | EPI_ISL_7661097 | EPI_ISL_8065790 | EPI_ISL_8065936 | EPI_ISL_8065875 | EPI_ISL_8065903 | EPI_ISL_12692077 |
| EPI_ISL_6088126 | EPI_ISL_7661100 | EPI_ISL_8065793 | EPI_ISL_8065937 | EPI_ISL_8065877 | EPI_ISL_8065904 | EPI_ISL_12692080 |
| EPI_ISL_6088133 | EPI_ISL_7661104 | EPI_ISL_8065794 | EPI_ISL_8065939 | EPI_ISL_8065879 | EPI_ISL_8065908 | EPI_ISL_12692082 |
| EPI_ISL_6088417 | EPI_ISL_7661108 | EPI_ISL_8065796 | EPI_ISL_8065940 | EPI_ISL_8065719 | EPI_ISL_8065909 | EPI_ISL_12692085 |
| EPI_ISL_6088440 | EPI_ISL_8065675 | EPI_ISL_8065806 | EPI_ISL_8065943 | EPI_ISL_8065721 | EPI_ISL_8065911 | EPI_ISL_12692086 |
| EPI_ISL_6088473 | EPI_ISL_8065677 | EPI_ISL_8065815 | EPI_ISL_8065946 | EPI_ISL_8065722 | EPI_ISL_8065915 | EPI_ISL_12692087 |
| EPI_ISL_6088487 | EPI_ISL_8065678 | EPI_ISL_8065817 | EPI_ISL_8065947 | EPI_ISL_8065723 | EPI_ISL_8065919 | EPI_ISL_12692088 |
| EPI_ISL_6088519 | EPI_ISL_8065683 | EPI_ISL_8065818 | EPI_ISL_8065952 | EPI_ISL_6944060 | EPI_ISL_8065727 | EPI_ISL_12692095 |
| EPI_ISL_6939025 | EPI_ISL_8065692 | EPI_ISL_8065826 | EPI_ISL_8065954 | EPI_ISL_6944061 | EPI_ISL_8065728 | EPI_ISL_12692099 |
| EPI_ISL_6939028 | EPI_ISL_8065693 | EPI_ISL_8065827 | EPI_ISL_8065955 | EPI_ISL_6944062 | EPI_ISL_8065733 | EPI_ISL_8065920 |
| EPI_ISL_6939037 | EPI_ISL_8065694 | EPI_ISL_8065832 | EPI_ISL_8065956 | EPI_ISL_7130064 | EPI_ISL_8065734 | EPI_ISL_8065922 |
| EPI_ISL_6939040 | EPI_ISL_8065695 | EPI_ISL_8065839 | EPI_ISL_8065964 | EPI_ISL_7130066 | EPI_ISL_8065735 | EPI_ISL_13392373 |
| EPI_ISL_6944016 | EPI_ISL_8065696 | EPI_ISL_8065840 | EPI_ISL_8065965 | EPI_ISL_7130067 | EPI_ISL_8065740 | EPI_ISL_13480636 |
| EPI_ISL_6944018 | EPI_ISL_8065697 | EPI_ISL_8065841 | EPI_ISL_10334288 | EPI_ISL_7130068 | EPI_ISL_8065741 | EPI_ISL_13480642 |
| EPI_ISL_6944020 | EPI_ISL_8065699 | EPI_ISL_8065843 | EPI_ISL_10334296 | EPI_ISL_7130076 | EPI_ISL_8065750 | EPI_ISL_13480652 |
| EPI_ISL_6944025 | EPI_ISL_8065702 | EPI_ISL_8065844 | EPI_ISL_10334299 | EPI_ISL_7130077 | EPI_ISL_8065751 | EPI_ISL_13480656 |
| EPI_ISL_6944026 | EPI_ISL_8065703 | EPI_ISL_8065849 | EPI_ISL_10334300 | EPI_ISL_7130078 | EPI_ISL_8065752 | EPI_ISL_13480659 |
| EPI_ISL_6944029 | EPI_ISL_8065708 | EPI_ISL_8065850 | EPI_ISL_10334301 | EPI_ISL_7130080 | EPI_ISL_8065754 | EPI_ISL_13480663 |
| EPI_ISL_6944038 | EPI_ISL_8065710 | EPI_ISL_8065853 | EPI_ISL_10334313 | EPI_ISL_7130081 | EPI_ISL_8065755 | EPI_ISL_13480667 |
| EPI_ISL_6944041 | EPI_ISL_8065712 | EPI_ISL_8065859 | EPI_ISL_10334372 | EPI_ISL_7130084 | EPI_ISL_8065760 | EPI_ISL_15014130 |
| EPI_ISL_6944047 | EPI_ISL_8065713 | EPI_ISL_8065860 | EPI_ISL_10334375 | EPI_ISL_7130090 | EPI_ISL_8065761 | EPI_ISL_15887025 |
| EPI_ISL_6944050 | EPI_ISL_8065717 | EPI_ISL_8065873 | EPI_ISL_10334552 | EPI_ISL_7130091 | EPI_ISL_15887039 | EPI_ISL_15887035 |
| EPI_ISL_6944054 | EPI_ISL_7130098 | EPI_ISL_15887045 | EPI_ISL_15887042 | EPI_ISL_7130097 | EPI_ISL_15887038 |  |

- England

| EPI_ISL_5480184 | EPI_ISL_3776546 | EPI_ISL_3952838 | EPI_ISL_4113242 | EPI_ISL_4129011 | EPI_ISL_4131529 | EPI_ISL_10151248 |
| --- | --- | --- | --- | --- | --- | --- |
| EPI_ISL_7161397 | EPI_ISL_3776579 | EPI_ISL_3953160 | EPI_ISL_4113337 | EPI_ISL_4129536 | EPI_ISL_4131538 | EPI_ISL_10151252 |
| EPI_ISL_8045382 | EPI_ISL_3776604 | EPI_ISL_3953223 | EPI_ISL_4114001 | EPI_ISL_4129551 | EPI_ISL_4132737 | EPI_ISL_10151253 |
| EPI_ISL_3726569 | EPI_ISL_3776614 | EPI_ISL_3953589 | EPI_ISL_4115156 | EPI_ISL_4129553 | EPI_ISL_4132928 | EPI_ISL_10151328 |
| EPI_ISL_3726593 | EPI_ISL_3776640 | EPI_ISL_3953702 | EPI_ISL_4115730 | EPI_ISL_4129561 | EPI_ISL_4133893 | EPI_ISL_10151330 |
| EPI_ISL_3726657 | EPI_ISL_3776659 | EPI_ISL_3953996 | EPI_ISL_4115924 | EPI_ISL_4129565 | EPI_ISL_4134083 | EPI_ISL_10151838 |
| EPI_ISL_3727066 | EPI_ISL_3784038 | EPI_ISL_3954494 | EPI_ISL_4116896 | EPI_ISL_4129571 | EPI_ISL_4134520 | EPI_ISL_10151840 |
| EPI_ISL_3727085 | EPI_ISL_3785024 | EPI_ISL_3954739 | EPI_ISL_4116901 | EPI_ISL_4129581 | EPI_ISL_4134527 | EPI_ISL_10152043 |
| EPI_ISL_3727086 | EPI_ISL_3807335 | EPI_ISL_3954741 | EPI_ISL_4116919 | EPI_ISL_4129603 | EPI_ISL_4134698 | EPI_ISL_10152070 |
| EPI_ISL_3727122 | EPI_ISL_3807478 | EPI_ISL_3954759 | EPI_ISL_4116981 | EPI_ISL_4129608 | EPI_ISL_4134763 | EPI_ISL_10152072 |
| EPI_ISL_3727125 | EPI_ISL_3807847 | EPI_ISL_3954860 | EPI_ISL_4117031 | EPI_ISL_4129693 | EPI_ISL_4134853 | EPI_ISL_10152089 |
| EPI_ISL_3727176 | EPI_ISL_3807860 | EPI_ISL_3954878 | EPI_ISL_4117039 | EPI_ISL_4129766 | EPI_ISL_4135500 | EPI_ISL_10152155 |
| EPI_ISL_3727737 | EPI_ISL_3808109 | EPI_ISL_3954921 | EPI_ISL_4117147 | EPI_ISL_4129767 | EPI_ISL_4135511 | EPI_ISL_10152398 |
| EPI_ISL_3727833 | EPI_ISL_3808210 | EPI_ISL_3954924 | EPI_ISL_4117167 | EPI_ISL_4129770 | EPI_ISL_4135894 | EPI_ISL_10152403 |
| EPI_ISL_3727962 | EPI_ISL_3808566 | EPI_ISL_3954925 | EPI_ISL_4117216 | EPI_ISL_4129786 | EPI_ISL_4135918 | EPI_ISL_10152528 |
| EPI_ISL_3728059 | EPI_ISL_3808580 | EPI_ISL_3954926 | EPI_ISL_4117267 | EPI_ISL_4129792 | EPI_ISL_4136100 | EPI_ISL_10152568 |
| EPI_ISL_3728071 | EPI_ISL_3808597 | EPI_ISL_3954957 | EPI_ISL_4117271 | EPI_ISL_4129797 | EPI_ISL_4136119 | EPI_ISL_10152569 |
| EPI_ISL_3728346 | EPI_ISL_3808603 | EPI_ISL_3954988 | EPI_ISL_4117294 | EPI_ISL_4129799 | EPI_ISL_4136373 | EPI_ISL_10152697 |
| EPI_ISL_3728359 | EPI_ISL_3808639 | EPI_ISL_3955068 | EPI_ISL_4117331 | EPI_ISL_4129801 | EPI_ISL_4136474 | EPI_ISL_10152699 |
| EPI_ISL_3728476 | EPI_ISL_3808754 | EPI_ISL_3955131 | EPI_ISL_4117821 | EPI_ISL_4129802 | EPI_ISL_4305217 | EPI_ISL_10152758 |
| EPI_ISL_3728562 | EPI_ISL_3808815 | EPI_ISL_3955189 | EPI_ISL_4119199 | EPI_ISL_4129806 | EPI_ISL_4305247 | EPI_ISL_10152947 |
| EPI_ISL_3728568 | EPI_ISL_3808907 | EPI_ISL_3955379 | EPI_ISL_4119205 | EPI_ISL_4129808 | EPI_ISL_4305275 | EPI_ISL_10152948 |
| EPI_ISL_3762922 | EPI_ISL_3808916 | EPI_ISL_3955481 | EPI_ISL_4119207 | EPI_ISL_4129831 | EPI_ISL_4305278 | EPI_ISL_10153026 |
| EPI_ISL_3763126 | EPI_ISL_3808983 | EPI_ISL_3955721 | EPI_ISL_4119585 | EPI_ISL_4129844 | EPI_ISL_4309450 | EPI_ISL_10153027 |
| EPI_ISL_3763277 | EPI_ISL_3809041 | EPI_ISL_3955779 | EPI_ISL_4119843 | EPI_ISL_4129850 | EPI_ISL_4309460 | EPI_ISL_10153032 |
| EPI_ISL_3763330 | EPI_ISL_3809151 | EPI_ISL_3955978 | EPI_ISL_4119989 | EPI_ISL_4129851 | EPI_ISL_4310254 | EPI_ISL_10153033 |
| EPI_ISL_3763346 | EPI_ISL_3809219 | EPI_ISL_3956162 | EPI_ISL_4120050 | EPI_ISL_4129857 | EPI_ISL_4310258 | EPI_ISL_10153157 |
| EPI_ISL_3763350 | EPI_ISL_3809510 | EPI_ISL_3956164 | EPI_ISL_4120052 | EPI_ISL_4129863 | EPI_ISL_4310724 | EPI_ISL_11934759 |
| EPI_ISL_3764083 | EPI_ISL_3809778 | EPI_ISL_3956182 | EPI_ISL_4120066 | EPI_ISL_4129866 | EPI_ISL_4311005 | EPI_ISL_3776392 |
| EPI_ISL_3764250 | EPI_ISL_3809793 | EPI_ISL_3956583 | EPI_ISL_4120166 | EPI_ISL_4129873 | EPI_ISL_4311008 | EPI_ISL_3776412 |
| EPI_ISL_3764442 | EPI_ISL_3809803 | EPI_ISL_3956912 | EPI_ISL_4120185 | EPI_ISL_4129876 | EPI_ISL_4311257 | EPI_ISL_3776430 |
| EPI_ISL_3764464 | EPI_ISL_3809910 | EPI_ISL_3957003 | EPI_ISL_4120284 | EPI_ISL_4129878 | EPI_ISL_4311755 | EPI_ISL_3776433 |
| EPI_ISL_3764504 | EPI_ISL_3809922 | EPI_ISL_3957088 | EPI_ISL_4120295 | EPI_ISL_4129879 | EPI_ISL_4311808 | EPI_ISL_3776451 |
| EPI_ISL_3764535 | EPI_ISL_3809978 | EPI_ISL_3957217 | EPI_ISL_4120310 | EPI_ISL_4129889 | EPI_ISL_4312175 | EPI_ISL_3776504 |
| EPI_ISL_3764780 | EPI_ISL_3809992 | EPI_ISL_3958242 | EPI_ISL_4120382 | EPI_ISL_4129890 | EPI_ISL_4312203 | EPI_ISL_3776509 |
| EPI_ISL_3764933 | EPI_ISL_3810002 | EPI_ISL_3959016 | EPI_ISL_4120384 | EPI_ISL_4129892 | EPI_ISL_4312229 | EPI_ISL_3776312 |
| EPI_ISL_3765000 | EPI_ISL_3810122 | EPI_ISL_3960894 | EPI_ISL_4120493 | EPI_ISL_4129895 | EPI_ISL_4312231 | EPI_ISL_3776319 |
| EPI_ISL_3765101 | EPI_ISL_3810126 | EPI_ISL_3962804 | EPI_ISL_4120571 | EPI_ISL_4129901 | EPI_ISL_4312232 | EPI_ISL_3776338 |
| EPI_ISL_3765135 | EPI_ISL_3810156 | EPI_ISL_3963862 | EPI_ISL_4120655 | EPI_ISL_4129918 | EPI_ISL_4312233 | EPI_ISL_3776348 |
| EPI_ISL_3765159 | EPI_ISL_3810179 | EPI_ISL_3964905 | EPI_ISL_4120657 | EPI_ISL_4129930 | EPI_ISL_4312234 | EPI_ISL_3776378 |
| EPI_ISL_3765615 | EPI_ISL_3810202 | EPI_ISL_3965160 | EPI_ISL_4120805 | EPI_ISL_4129949 | EPI_ISL_4312250 | EPI_ISL_3776384 |
| EPI_ISL_3765645 | EPI_ISL_3810232 | EPI_ISL_3965249 | EPI_ISL_4120973 | EPI_ISL_4129968 | EPI_ISL_4312252 | EPI_ISL_3776391 |
| EPI_ISL_3766061 | EPI_ISL_3810510 | EPI_ISL_3965373 | EPI_ISL_4120975 | EPI_ISL_4129975 | EPI_ISL_4312259 | EPI_ISL_3950621 |
| EPI_ISL_3766153 | EPI_ISL_3810673 | EPI_ISL_3966600 | EPI_ISL_4121052 | EPI_ISL_4129980 | EPI_ISL_4312260 | EPI_ISL_3950942 |
| EPI_ISL_3766341 | EPI_ISL_3810712 | EPI_ISL_3966760 | EPI_ISL_4121081 | EPI_ISL_4129983 | EPI_ISL_4312264 | EPI_ISL_3951250 |
| EPI_ISL_3766700 | EPI_ISL_3810774 | EPI_ISL_3967048 | EPI_ISL_4121083 | EPI_ISL_4129987 | EPI_ISL_4312265 | EPI_ISL_3951426 |
| EPI_ISL_3766958 | EPI_ISL_3811506 | EPI_ISL_3967695 | EPI_ISL_4121438 | EPI_ISL_4130003 | EPI_ISL_4312271 | EPI_ISL_3951736 |
| EPI_ISL_3767148 | EPI_ISL_3811523 | EPI_ISL_3967827 | EPI_ISL_4121860 | EPI_ISL_4130004 | EPI_ISL_4312278 | EPI_ISL_3952074 |
| EPI_ISL_3767341 | EPI_ISL_3811557 | EPI_ISL_3967842 | EPI_ISL_4121873 | EPI_ISL_4130017 | EPI_ISL_4312290 | EPI_ISL_3952132 |
| EPI_ISL_3767484 | EPI_ISL_3811561 | EPI_ISL_3967941 | EPI_ISL_4121883 | EPI_ISL_4130022 | EPI_ISL_4312292 | EPI_ISL_3952465 |
| EPI_ISL_3767558 | EPI_ISL_3811683 | EPI_ISL_3968090 | EPI_ISL_4121989 | EPI_ISL_4130023 | EPI_ISL_4312320 | EPI_ISL_3952623 |
| EPI_ISL_3767643 | EPI_ISL_3811725 | EPI_ISL_3968268 | EPI_ISL_4122206 | EPI_ISL_4130031 | EPI_ISL_4312331 | EPI_ISL_3952679 |
| EPI_ISL_3767734 | EPI_ISL_3811914 | EPI_ISL_3968429 | EPI_ISL_4122227 | EPI_ISL_4130034 | EPI_ISL_4312335 | EPI_ISL_3952825 |
| EPI_ISL_3767907 | EPI_ISL_3811915 | EPI_ISL_3968477 | EPI_ISL_4122233 | EPI_ISL_4130035 | EPI_ISL_4313121 | EPI_ISL_3994585 |
| EPI_ISL_3768053 | EPI_ISL_3812066 | EPI_ISL_3968681 | EPI_ISL_4122337 | EPI_ISL_4130046 | EPI_ISL_4313196 | EPI_ISL_3994608 |
| EPI_ISL_3768058 | EPI_ISL_3812187 | EPI_ISL_3968738 | EPI_ISL_4122367 | EPI_ISL_4130048 | EPI_ISL_4313282 | EPI_ISL_3994660 |
| EPI_ISL_3768068 | EPI_ISL_3812405 | EPI_ISL_3968757 | EPI_ISL_4122398 | EPI_ISL_4130049 | EPI_ISL_4313288 | EPI_ISL_3995861 |
| EPI_ISL_3768221 | EPI_ISL_3836581 | EPI_ISL_3968767 | EPI_ISL_4122402 | EPI_ISL_4130051 | EPI_ISL_4314471 | EPI_ISL_3996592 |
| EPI_ISL_3768247 | EPI_ISL_3836670 | EPI_ISL_3968790 | EPI_ISL_4122413 | EPI_ISL_4130058 | EPI_ISL_4314475 | EPI_ISL_4020368 |
| EPI_ISL_3768265 | EPI_ISL_3837044 | EPI_ISL_3968909 | EPI_ISL_4122426 | EPI_ISL_4130059 | EPI_ISL_4314505 | EPI_ISL_4045893 |
| EPI_ISL_3768283 | EPI_ISL_3837161 | EPI_ISL_3969877 | EPI_ISL_4122621 | EPI_ISL_4130060 | EPI_ISL_4531312 | EPI_ISL_4112496 |
| EPI_ISL_3768487 | EPI_ISL_3837223 | EPI_ISL_3970092 | EPI_ISL_4122636 | EPI_ISL_4130063 | EPI_ISL_4532045 | EPI_ISL_4113197 |
| EPI_ISL_3768501 | EPI_ISL_3837330 | EPI_ISL_3970114 | EPI_ISL_4122654 | EPI_ISL_4130067 | EPI_ISL_4532046 | EPI_ISL_4113232 |
| EPI_ISL_3768572 | EPI_ISL_3837608 | EPI_ISL_3970150 | EPI_ISL_4122658 | EPI_ISL_4130074 | EPI_ISL_4532071 | EPI_ISL_4113240 |
| EPI_ISL_3768733 | EPI_ISL_3837673 | EPI_ISL_3970261 | EPI_ISL_4123902 | EPI_ISL_4130076 | EPI_ISL_4532834 | EPI_ISL_4128809 |
| EPI_ISL_3775592 | EPI_ISL_3882272 | EPI_ISL_3970711 | EPI_ISL_4124252 | EPI_ISL_4130080 | EPI_ISL_4771372 | EPI_ISL_4128825 |
| EPI_ISL_3775602 | EPI_ISL_3883140 | EPI_ISL_3970786 | EPI_ISL_4125638 | EPI_ISL_4130092 | EPI_ISL_4781102 | EPI_ISL_4128869 |
| EPI_ISL_3775614 | EPI_ISL_3883382 | EPI_ISL_3970923 | EPI_ISL_4127220 | EPI_ISL_4130093 | EPI_ISL_4781113 | EPI_ISL_4128879 |
| EPI_ISL_3775634 | EPI_ISL_3883551 | EPI_ISL_3990405 | EPI_ISL_4128588 | EPI_ISL_4130758 | EPI_ISL_4781115 | EPI_ISL_4128922 |
| EPI_ISL_3775638 | EPI_ISL_3883625 | EPI_ISL_3990472 | EPI_ISL_4128590 | EPI_ISL_4130765 | EPI_ISL_4781117 | EPI_ISL_4128932 |
| EPI_ISL_3775651 | EPI_ISL_3883758 | EPI_ISL_3990479 | EPI_ISL_4128598 | EPI_ISL_4130846 | EPI_ISL_4800216 | EPI_ISL_4128957 |
| EPI_ISL_3775660 | EPI_ISL_3883886 | EPI_ISL_3990533 | EPI_ISL_4128602 | EPI_ISL_4130851 | EPI_ISL_5040552 | EPI_ISL_4128975 |
| EPI_ISL_3775665 | EPI_ISL_3883922 | EPI_ISL_3991650 | EPI_ISL_4128610 | EPI_ISL_4130856 | EPI_ISL_5042269 | EPI_ISL_4128992 |
| EPI_ISL_3775667 | EPI_ISL_3884171 | EPI_ISL_3991657 | EPI_ISL_4128614 | EPI_ISL_4130862 | EPI_ISL_7362558 | EPI_ISL_4129004 |
| EPI_ISL_3775699 | EPI_ISL_3884871 | EPI_ISL_3992472 | EPI_ISL_4128618 | EPI_ISL_4130864 | EPI_ISL_7362567 | EPI_ISL_4129006 |
| EPI_ISL_3775749 | EPI_ISL_3885180 | EPI_ISL_3992932 | EPI_ISL_4128622 | EPI_ISL_4131003 | EPI_ISL_7362704 | EPI_ISL_4131413 |
| EPI_ISL_3775752 | EPI_ISL_3915627 | EPI_ISL_3992974 | EPI_ISL_4128711 | EPI_ISL_4131005 | EPI_ISL_7362729 | EPI_ISL_4131417 |
| EPI_ISL_3775808 | EPI_ISL_3950301 | EPI_ISL_3993029 | EPI_ISL_4128754 | EPI_ISL_4131008 | EPI_ISL_7362757 | EPI_ISL_4131458 |
| EPI_ISL_3775908 | EPI_ISL_3950408 | EPI_ISL_3994578 | EPI_ISL_4128781 | EPI_ISL_4131076 | EPI_ISL_7362963 | EPI_ISL_4131481 |
| EPI_ISL_10150914 | EPI_ISL_10150813 | EPI_ISL_10150716 | EPI_ISL_4131318 | EPI_ISL_4131190 | EPI_ISL_7363548 | EPI_ISL_4131505 |
| EPI_ISL_10151066 | EPI_ISL_10150910 | EPI_ISL_10150794 | EPI_ISL_4131404 | EPI_ISL_4131215 | EPI_ISL_10150344 | EPI_ISL_4131410 |
| EPI_ISL_10150715 | EPI_ISL_10150527 | EPI_ISL_10150429 | EPI_ISL_4131241 |  |  |  |

- USA

| EPI_ISL_4026867 | EPI_ISL_4004964 | EPI_ISL_4160991 | EPI_ISL_4399431 | EPI_ISL_5344773 | EPI_ISL_5345289 | EPI_ISL_17114596 |
| --- | --- | --- | --- | --- | --- | --- |
| EPI_ISL_4026880 | EPI_ISL_4021245 | EPI_ISL_4160994 | EPI_ISL_4399492 | EPI_ISL_5344803 | EPI_ISL_5345292 | EPI_ISL_17126181 |
| EPI_ISL_4161270 | EPI_ISL_4021579 | EPI_ISL_4161002 | EPI_ISL_4399596 | EPI_ISL_5344814 | EPI_ISL_5345293 | EPI_ISL_4245349 |
| EPI_ISL_4182591 | EPI_ISL_4022273 | EPI_ISL_4161017 | EPI_ISL_4400388 | EPI_ISL_5344831 | EPI_ISL_5345294 | EPI_ISL_4245443 |
| EPI_ISL_4246559 | EPI_ISL_4022311 | EPI_ISL_4161019 | EPI_ISL_4400390 | EPI_ISL_5344832 | EPI_ISL_5345296 | EPI_ISL_4262224 |
| EPI_ISL_4246595 | EPI_ISL_4022400 | EPI_ISL_4161418 | EPI_ISL_4400404 | EPI_ISL_5344841 | EPI_ISL_5345298 | EPI_ISL_4296761 |
| EPI_ISL_4246623 | EPI_ISL_4022404 | EPI_ISL_4161572 | EPI_ISL_4401403 | EPI_ISL_5344843 | EPI_ISL_5345299 | EPI_ISL_4296790 |
| EPI_ISL_4246701 | EPI_ISL_4022405 | EPI_ISL_4161667 | EPI_ISL_4401664 | EPI_ISL_5344889 | EPI_ISL_5345302 | EPI_ISL_4296807 |
| EPI_ISL_4344632 | EPI_ISL_4022408 | EPI_ISL_4163930 | EPI_ISL_4401669 | EPI_ISL_5344915 | EPI_ISL_5345304 | EPI_ISL_4302186 |
| EPI_ISL_4456990 | EPI_ISL_4022409 | EPI_ISL_4163933 | EPI_ISL_4401781 | EPI_ISL_5344917 | EPI_ISL_5345305 | EPI_ISL_4331545 |
| EPI_ISL_4673667 | EPI_ISL_4022411 | EPI_ISL_4163934 | EPI_ISL_4401821 | EPI_ISL_5344936 | EPI_ISL_5345309 | EPI_ISL_4339688 |
| EPI_ISL_4886948 | EPI_ISL_4022433 | EPI_ISL_4163940 | EPI_ISL_4410877 | EPI_ISL_5344937 | EPI_ISL_5345316 | EPI_ISL_4340621 |
| EPI_ISL_5080851 | EPI_ISL_4022439 | EPI_ISL_4163945 | EPI_ISL_4410955 | EPI_ISL_5344941 | EPI_ISL_5345326 | EPI_ISL_4340629 |
| EPI_ISL_5157136 | EPI_ISL_4022441 | EPI_ISL_4163950 | EPI_ISL_4418685 | EPI_ISL_5344951 | EPI_ISL_5345329 | EPI_ISL_4340637 |
| EPI_ISL_5174146 | EPI_ISL_4022447 | EPI_ISL_4163954 | EPI_ISL_4434994 | EPI_ISL_5344954 | EPI_ISL_5345345 | EPI_ISL_4340654 |
| EPI_ISL_5260328 | EPI_ISL_4022448 | EPI_ISL_4163956 | EPI_ISL_4435278 | EPI_ISL_5344956 | EPI_ISL_5345348 | EPI_ISL_4341066 |
| EPI_ISL_5548048 | EPI_ISL_4022449 | EPI_ISL_4163992 | EPI_ISL_4468483 | EPI_ISL_5344958 | EPI_ISL_5345354 | EPI_ISL_4341067 |
| EPI_ISL_5576239 | EPI_ISL_4022479 | EPI_ISL_4164000 | EPI_ISL_4507251 | EPI_ISL_5344964 | EPI_ISL_5345357 | EPI_ISL_4341428 |
| EPI_ISL_5580990 | EPI_ISL_4022484 | EPI_ISL_4164050 | EPI_ISL_4507298 | EPI_ISL_5344971 | EPI_ISL_5345360 | EPI_ISL_4385026 |
| EPI_ISL_5586128 | EPI_ISL_4022494 | EPI_ISL_4166736 | EPI_ISL_4511430 | EPI_ISL_5344989 | EPI_ISL_5345373 | EPI_ISL_4385070 |
| EPI_ISL_5586129 | EPI_ISL_4022501 | EPI_ISL_4169205 | EPI_ISL_4512233 | EPI_ISL_5344991 | EPI_ISL_5345374 | EPI_ISL_4392235 |
| EPI_ISL_5586133 | EPI_ISL_4022502 | EPI_ISL_4169684 | EPI_ISL_4512282 | EPI_ISL_5344993 | EPI_ISL_5345380 | EPI_ISL_4398779 |
| EPI_ISL_5612525 | EPI_ISL_4022524 | EPI_ISL_4173010 | EPI_ISL_4512289 | EPI_ISL_5344995 | EPI_ISL_5345383 | EPI_ISL_4398878 |
| EPI_ISL_5612694 | EPI_ISL_4022534 | EPI_ISL_4173022 | EPI_ISL_4512292 | EPI_ISL_5344997 | EPI_ISL_5345402 | EPI_ISL_4398918 |
| EPI_ISL_6035496 | EPI_ISL_4022608 | EPI_ISL_4173419 | EPI_ISL_4518894 | EPI_ISL_5345000 | EPI_ISL_5345408 | EPI_ISL_4399390 |
| EPI_ISL_6079202 | EPI_ISL_4022623 | EPI_ISL_4173536 | EPI_ISL_4549721 | EPI_ISL_5345012 | EPI_ISL_5345426 | EPI_ISL_5225988 |
| EPI_ISL_6390654 | EPI_ISL_4022632 | EPI_ISL_4175887 | EPI_ISL_4556153 | EPI_ISL_5345017 | EPI_ISL_5345433 | EPI_ISL_5226114 |
| EPI_ISL_6572087 | EPI_ISL_4022652 | EPI_ISL_4180318 | EPI_ISL_4557061 | EPI_ISL_5345018 | EPI_ISL_5345435 | EPI_ISL_5226685 |
| EPI_ISL_6572238 | EPI_ISL_4022954 | EPI_ISL_4180398 | EPI_ISL_4568624 | EPI_ISL_5345028 | EPI_ISL_5345438 | EPI_ISL_5226738 |
| EPI_ISL_6792508 | EPI_ISL_4022958 | EPI_ISL_4180407 | EPI_ISL_4578141 | EPI_ISL_5345030 | EPI_ISL_5345440 | EPI_ISL_5228857 |
| EPI_ISL_6907498 | EPI_ISL_4022963 | EPI_ISL_4180422 | EPI_ISL_4625211 | EPI_ISL_5345031 | EPI_ISL_5345442 | EPI_ISL_5228909 |
| EPI_ISL_7312010 | EPI_ISL_4022964 | EPI_ISL_4181211 | EPI_ISL_4625223 | EPI_ISL_5345035 | EPI_ISL_5345445 | EPI_ISL_5229157 |
| EPI_ISL_7568246 | EPI_ISL_4023111 | EPI_ISL_4193816 | EPI_ISL_4625254 | EPI_ISL_5345056 | EPI_ISL_5345453 | EPI_ISL_5229195 |
| EPI_ISL_7741558 | EPI_ISL_4023119 | EPI_ISL_4197363 | EPI_ISL_4625259 | EPI_ISL_5345063 | EPI_ISL_5345455 | EPI_ISL_5229198 |
| EPI_ISL_7742416 | EPI_ISL_4024394 | EPI_ISL_4197367 | EPI_ISL_4625312 | EPI_ISL_5345064 | EPI_ISL_5345459 | EPI_ISL_5229303 |
| EPI_ISL_8294918 | EPI_ISL_4024397 | EPI_ISL_4197370 | EPI_ISL_4658910 | EPI_ISL_5345066 | EPI_ISL_5345464 | EPI_ISL_5230279 |
| EPI_ISL_8472164 | EPI_ISL_4027039 | EPI_ISL_4197371 | EPI_ISL_4660019 | EPI_ISL_5345067 | EPI_ISL_5345469 | EPI_ISL_5230598 |
| EPI_ISL_8566991 | EPI_ISL_4027047 | EPI_ISL_4197372 | EPI_ISL_4667655 | EPI_ISL_5345068 | EPI_ISL_5345482 | EPI_ISL_5231549 |
| EPI_ISL_9579791 | EPI_ISL_4027063 | EPI_ISL_4197377 | EPI_ISL_4702376 | EPI_ISL_5345069 | EPI_ISL_5345505 | EPI_ISL_5232389 |
| EPI_ISL_9923426 | EPI_ISL_4027064 | EPI_ISL_4201895 | EPI_ISL_4728078 | EPI_ISL_5345070 | EPI_ISL_5345554 | EPI_ISL_5232393 |
| EPI_ISL_11171626 | EPI_ISL_4028464 | EPI_ISL_4201897 | EPI_ISL_4728085 | EPI_ISL_5345091 | EPI_ISL_5345571 | EPI_ISL_5232410 |
| EPI_ISL_11253193 | EPI_ISL_4028770 | EPI_ISL_4201899 | EPI_ISL_4744753 | EPI_ISL_5345092 | EPI_ISL_5345598 | EPI_ISL_5236736 |
| EPI_ISL_12233708 | EPI_ISL_4028922 | EPI_ISL_4201900 | EPI_ISL_4762205 | EPI_ISL_5345093 | EPI_ISL_5345629 | EPI_ISL_5290297 |
| EPI_ISL_17115320 | EPI_ISL_4028937 | EPI_ISL_4202245 | EPI_ISL_4847325 | EPI_ISL_5345094 | EPI_ISL_5345678 | EPI_ISL_5344692 |
| EPI_ISL_17116663 | EPI_ISL_4028956 | EPI_ISL_4202254 | EPI_ISL_4868115 | EPI_ISL_5345100 | EPI_ISL_5354252 | EPI_ISL_5344709 |
| EPI_ISL_3760959 | EPI_ISL_4072188 | EPI_ISL_4202267 | EPI_ISL_4921019 | EPI_ISL_5345106 | EPI_ISL_5421471 | EPI_ISL_5344751 |
| EPI_ISL_3762010 | EPI_ISL_4072198 | EPI_ISL_4202305 | EPI_ISL_4921850 | EPI_ISL_5345111 | EPI_ISL_5523585 | EPI_ISL_5344764 |
| EPI_ISL_3762038 | EPI_ISL_4139298 | EPI_ISL_4202434 | EPI_ISL_4922556 | EPI_ISL_5345121 | EPI_ISL_5523586 | EPI_ISL_5344766 |
| EPI_ISL_3762050 | EPI_ISL_4139400 | EPI_ISL_4202456 | EPI_ISL_4922595 | EPI_ISL_5345129 | EPI_ISL_5523594 | EPI_ISL_5345217 |
| EPI_ISL_3762080 | EPI_ISL_4139580 | EPI_ISL_4202472 | EPI_ISL_4924688 | EPI_ISL_5345134 | EPI_ISL_5523598 | EPI_ISL_5345218 |
| EPI_ISL_3762083 | EPI_ISL_4140853 | EPI_ISL_4202473 | EPI_ISL_4926362 | EPI_ISL_5345138 | EPI_ISL_5565781 | EPI_ISL_5345220 |
| EPI_ISL_3762088 | EPI_ISL_4141933 | EPI_ISL_4202477 | EPI_ISL_4940138 | EPI_ISL_5345139 | EPI_ISL_5604829 | EPI_ISL_5345221 |
| EPI_ISL_3762156 | EPI_ISL_4142078 | EPI_ISL_4202479 | EPI_ISL_4957831 | EPI_ISL_5345142 | EPI_ISL_5605147 | EPI_ISL_5345223 |
| EPI_ISL_3762183 | EPI_ISL_4142093 | EPI_ISL_4202491 | EPI_ISL_5095188 | EPI_ISL_5345146 | EPI_ISL_5628610 | EPI_ISL_5345227 |
| EPI_ISL_3762233 | EPI_ISL_4152174 | EPI_ISL_4202496 | EPI_ISL_5095189 | EPI_ISL_5345150 | EPI_ISL_5689570 | EPI_ISL_5345230 |
| EPI_ISL_3762235 | EPI_ISL_4152176 | EPI_ISL_4202507 | EPI_ISL_5095191 | EPI_ISL_5345151 | EPI_ISL_5986637 | EPI_ISL_5345232 |
| EPI_ISL_3762335 | EPI_ISL_4152180 | EPI_ISL_4202695 | EPI_ISL_5153081 | EPI_ISL_5345153 | EPI_ISL_6253440 | EPI_ISL_5345233 |
| EPI_ISL_3762367 | EPI_ISL_4160571 | EPI_ISL_4202750 | EPI_ISL_5153085 | EPI_ISL_5345155 | EPI_ISL_6253462 | EPI_ISL_5345236 |
| EPI_ISL_3777663 | EPI_ISL_4160575 | EPI_ISL_4210607 | EPI_ISL_5153092 | EPI_ISL_5345156 | EPI_ISL_6253463 | EPI_ISL_5345240 |
| EPI_ISL_3777991 | EPI_ISL_4160678 | EPI_ISL_4210618 | EPI_ISL_5153119 | EPI_ISL_5345162 | EPI_ISL_6337500 | EPI_ISL_5345251 |
| EPI_ISL_3778426 | EPI_ISL_4160814 | EPI_ISL_4212070 | EPI_ISL_5155109 | EPI_ISL_5345166 | EPI_ISL_6406464 | EPI_ISL_5345270 |
| EPI_ISL_3899806 | EPI_ISL_4160860 | EPI_ISL_4238655 | EPI_ISL_5155361 | EPI_ISL_5345168 | EPI_ISL_6406508 | EPI_ISL_5345272 |
| EPI_ISL_3900696 | EPI_ISL_4160925 | EPI_ISL_4239478 | EPI_ISL_5155513 | EPI_ISL_5345170 | EPI_ISL_6411001 | EPI_ISL_5345275 |
| EPI_ISL_3900712 | EPI_ISL_4160937 | EPI_ISL_4240028 | EPI_ISL_5195119 | EPI_ISL_5345183 | EPI_ISL_6411059 | EPI_ISL_5345276 |
| EPI_ISL_3906076 | EPI_ISL_4160965 | EPI_ISL_4240453 | EPI_ISL_5199650 | EPI_ISL_5345187 | EPI_ISL_6475816 | EPI_ISL_5345277 |
| EPI_ISL_3906108 | EPI_ISL_4160979 | EPI_ISL_4240900 | EPI_ISL_5204554 | EPI_ISL_5345190 | EPI_ISL_6475881 | EPI_ISL_5345278 |
| EPI_ISL_3944263 | EPI_ISL_4160982 | EPI_ISL_4245330 | EPI_ISL_5204556 | EPI_ISL_5345214 | EPI_ISL_6493631 | EPI_ISL_5345282 |
| EPI_ISL_7987232 | EPI_ISL_4160985 | EPI_ISL_10402223 | EPI_ISL_14794081 | EPI_ISL_17114541 | EPI_ISL_6568517 | EPI_ISL_5345283 |
| EPI_ISL_9540000 | EPI_ISL_6855194 | EPI_ISL_11301930 | EPI_ISL_15569230 | EPI_ISL_17114548 | EPI_ISL_6568529 | EPI_ISL_5345285 |
| EPI_ISL_9772597 | EPI_ISL_6855207 | EPI_ISL_12171580 | EPI_ISL_16124139 | EPI_ISL_17114549 | EPI_ISL_6568592 | EPI_ISL_5345287 |
| EPI_ISL_9773618 | EPI_ISL_6855443 | EPI_ISL_12171582 | EPI_ISL_16472671 | EPI_ISL_17114573 | EPI_ISL_6604118 | EPI_ISL_5345288 |

- Australia

| EPI_ISL_7265080 | EPI_ISL_4777784 | EPI_ISL_5535902 | EPI_ISL_5054907 | EPI_ISL_5656390 | EPI_ISL_8641986 | EPI_ISL_9302708 |
| --- | --- | --- | --- | --- | --- | --- |
| EPI_ISL_7288205 | EPI_ISL_4777789 | EPI_ISL_5535903 | EPI_ISL_5054908 | EPI_ISL_5778469 | EPI_ISL_8642037 | EPI_ISL_9303023 |
| EPI_ISL_7621547 | EPI_ISL_4777802 | EPI_ISL_5649492 | EPI_ISL_5054909 | EPI_ISL_5778470 | EPI_ISL_8642169 | EPI_ISL_9767721 |
| EPI_ISL_7621977 | EPI_ISL_4777827 | EPI_ISL_5649504 | EPI_ISL_5305455 | EPI_ISL_5778578 | EPI_ISL_8642180 | EPI_ISL_9951667 |
| EPI_ISL_7709574 | EPI_ISL_4777836 | EPI_ISL_5649508 | EPI_ISL_5305456 | EPI_ISL_5778603 | EPI_ISL_8642253 | EPI_ISL_9951669 |
| EPI_ISL_7709579 | EPI_ISL_4777867 | EPI_ISL_5649513 | EPI_ISL_5305457 | EPI_ISL_5778640 | EPI_ISL_8642340 | EPI_ISL_10561090 |
| EPI_ISL_7768173 | EPI_ISL_4777880 | EPI_ISL_5649519 | EPI_ISL_5535901 | EPI_ISL_5804834 | EPI_ISL_8642397 | EPI_ISL_10561102 |
| EPI_ISL_8331615 | EPI_ISL_4777892 | EPI_ISL_5649520 | EPI_ISL_4762266 | EPI_ISL_5804836 | EPI_ISL_8642425 | EPI_ISL_10561134 |
| EPI_ISL_8640639 | EPI_ISL_4786077 | EPI_ISL_5649524 | EPI_ISL_4762268 | EPI_ISL_6267946 | EPI_ISL_8642509 | EPI_ISL_10561145 |
| EPI_ISL_8641059 | EPI_ISL_4786093 | EPI_ISL_5656386 | EPI_ISL_4762269 | EPI_ISL_6588518 | EPI_ISL_8642857 | EPI_ISL_10561151 |
| EPI_ISL_8641654 | EPI_ISL_4786122 | EPI_ISL_7825540 | EPI_ISL_4762270 | EPI_ISL_6589113 | EPI_ISL_8642896 | EPI_ISL_10561188 |
| EPI_ISL_10260041 | EPI_ISL_4786144 | EPI_ISL_7825541 | EPI_ISL_4777661 | EPI_ISL_6589146 | EPI_ISL_8643123 | EPI_ISL_10561292 |
| EPI_ISL_3693303 | EPI_ISL_4877353 | EPI_ISL_7825542 | EPI_ISL_4777697 | EPI_ISL_6589805 | EPI_ISL_8643180 | EPI_ISL_10561293 |
| EPI_ISL_4254948 | EPI_ISL_4881032 | EPI_ISL_7825544 | EPI_ISL_4777712 | EPI_ISL_6590364 | EPI_ISL_8643285 | EPI_ISL_10561583 |
| EPI_ISL_4263109 | EPI_ISL_4881124 | EPI_ISL_7825545 | EPI_ISL_7748332 | EPI_ISL_6590488 | EPI_ISL_8643294 | EPI_ISL_10565680 |
| EPI_ISL_4263110 | EPI_ISL_4881139 | EPI_ISL_7825546 | EPI_ISL_7748341 | EPI_ISL_6827506 | EPI_ISL_8643296 | EPI_ISL_12701685 |
| EPI_ISL_4263113 | EPI_ISL_4881395 | EPI_ISL_7825547 | EPI_ISL_7749522 | EPI_ISL_7141550 | EPI_ISL_8643504 | EPI_ISL_12846185 |
| EPI_ISL_4303404 | EPI_ISL_4881780 | EPI_ISL_7825549 | EPI_ISL_7765931 | EPI_ISL_7161992 | EPI_ISL_8643607 | EPI_ISL_16360579 |
| EPI_ISL_4303408 | EPI_ISL_4881801 | EPI_ISL_7825550 | EPI_ISL_7765986 | EPI_ISL_7190299 | EPI_ISL_8645930 | EPI_ISL_16825899 |
| EPI_ISL_4346858 | EPI_ISL_4881814 | EPI_ISL_7825552 | EPI_ISL_7766519 | EPI_ISL_7190306 | EPI_ISL_8646080 | EPI_ISL_17374644 |
| EPI_ISL_4348498 | EPI_ISL_4881817 | EPI_ISL_7825558 | EPI_ISL_7767945 | EPI_ISL_7190314 | EPI_ISL_8646179 | EPI_ISL_17720716 |
| EPI_ISL_4348508 | EPI_ISL_4881823 | EPI_ISL_8211023 | EPI_ISL_7768068 | EPI_ISL_7190318 | EPI_ISL_8646273 | EPI_ISL_17811785 |
| EPI_ISL_4348522 | EPI_ISL_4881840 | EPI_ISL_8331521 | EPI_ISL_7768161 | EPI_ISL_7190327 | EPI_ISL_8646312 | EPI_ISL_17816263 |
| EPI_ISL_4762246 | EPI_ISL_4881844 | EPI_ISL_8333768 | EPI_ISL_7768204 | EPI_ISL_7264328 | EPI_ISL_8692463 | EPI_ISL_17999525 |
| EPI_ISL_4762248 | EPI_ISL_4881853 | EPI_ISL_8559400 | EPI_ISL_7769503 | EPI_ISL_7264720 | EPI_ISL_8768542 | EPI_ISL_17999528 |
| EPI_ISL_4762251 | EPI_ISL_5033180 | EPI_ISL_8597951 | EPI_ISL_7769507 | EPI_ISL_7264798 | EPI_ISL_8954806 | EPI_ISL_17999714 |
| EPI_ISL_4762252 | EPI_ISL_5033192 | EPI_ISL_8640209 | EPI_ISL_7769546 | EPI_ISL_7264941 | EPI_ISL_8955015 | EPI_ISL_17999759 |
| EPI_ISL_4762253 | EPI_ISL_5033193 | EPI_ISL_8640334 | EPI_ISL_7769654 | EPI_ISL_7265235 | EPI_ISL_8955017 | EPI_ISL_17999956 |
| EPI_ISL_4762254 | EPI_ISL_5033338 | EPI_ISL_8640507 | EPI_ISL_7770173 | EPI_ISL_7265254 | EPI_ISL_8955023 | EPI_ISL_4762263 |
| EPI_ISL_4762255 | EPI_ISL_5033341 | EPI_ISL_8640653 | EPI_ISL_7770256 | EPI_ISL_7569959 | EPI_ISL_8955360 | EPI_ISL_4762264 |
| EPI_ISL_4762257 | EPI_ISL_5033343 | EPI_ISL_8640734 | EPI_ISL_7770473 | EPI_ISL_7569963 | EPI_ISL_8955382 | EPI_ISL_4762265 |
| EPI_ISL_4762258 | EPI_ISL_5033344 | EPI_ISL_8641080 | EPI_ISL_7770565 | EPI_ISL_7569973 | EPI_ISL_8955691 | EPI_ISL_5054904 |
| EPI_ISL_4762259 | EPI_ISL_5033347 | EPI_ISL_8641113 | EPI_ISL_7771160 | EPI_ISL_7569995 | EPI_ISL_9285726 | EPI_ISL_5054905 |
| EPI_ISL_4762260 | EPI_ISL_5054901 | EPI_ISL_8641207 | EPI_ISL_7772209 | EPI_ISL_7621329 | EPI_ISL_9285730 | EPI_ISL_5054906 |
| EPI_ISL_4762261 | EPI_ISL_5054902 | EPI_ISL_8641280 | EPI_ISL_7772601 | EPI_ISL_7621487 | EPI_ISL_9301970 | EPI_ISL_8641619 |
| EPI_ISL_4762262 | EPI_ISL_5054903 | EPI_ISL_8641285 | EPI_ISL_7773906 | EPI_ISL_7621492 | EPI_ISL_9301974 | EPI_ISL_8641675 |
| EPI_ISL_7621759 | EPI_ISL_9302701 | EPI_ISL_8641523 | EPI_ISL_9302596 | EPI_ISL_7621720 | EPI_ISL_9302362 | EPI_ISL_7621721 |

1. Omicron

- China

| EPI_ISL_11873915 | EPI_ISL_16610270 | EPI_ISL_16923319 | EPI_ISL_16923416 | EPI_ISL_16923769 | EPI_ISL_16923859 | EPI_ISL_16923940 |
| --- | --- | --- | --- | --- | --- | --- |
| EPI_ISL_11873917 | EPI_ISL_16610273 | EPI_ISL_16923320 | EPI_ISL_16923417 | EPI_ISL_16923770 | EPI_ISL_16923860 | EPI_ISL_16923941 |
| EPI_ISL_11873919 | EPI_ISL_16610276 | EPI_ISL_16923321 | EPI_ISL_16923418 | EPI_ISL_16923771 | EPI_ISL_16923861 | EPI_ISL_16923942 |
| EPI_ISL_11873925 | EPI_ISL_16610277 | EPI_ISL_16923322 | EPI_ISL_16923419 | EPI_ISL_16923772 | EPI_ISL_16923862 | EPI_ISL_16923943 |
| EPI_ISL_11873928 | EPI_ISL_16610279 | EPI_ISL_16923323 | EPI_ISL_16923420 | EPI_ISL_16923773 | EPI_ISL_16923863 | EPI_ISL_16923945 |
| EPI_ISL_11873929 | EPI_ISL_16610285 | EPI_ISL_16923324 | EPI_ISL_16923421 | EPI_ISL_16923774 | EPI_ISL_16923864 | EPI_ISL_16923948 |
| EPI_ISL_11873937 | EPI_ISL_16610286 | EPI_ISL_16923325 | EPI_ISL_16923422 | EPI_ISL_16923775 | EPI_ISL_16923865 | EPI_ISL_16923949 |
| EPI_ISL_11905843 | EPI_ISL_16610287 | EPI_ISL_16923326 | EPI_ISL_16923423 | EPI_ISL_16923776 | EPI_ISL_16923866 | EPI_ISL_16923950 |
| EPI_ISL_11905848 | EPI_ISL_16610289 | EPI_ISL_16923327 | EPI_ISL_16923424 | EPI_ISL_16923777 | EPI_ISL_16923867 | EPI_ISL_16923953 |
| EPI_ISL_12030355 | EPI_ISL_16610290 | EPI_ISL_16923328 | EPI_ISL_16923425 | EPI_ISL_16923778 | EPI_ISL_16923868 | EPI_ISL_17104929 |
| EPI_ISL_12030356 | EPI_ISL_16610306 | EPI_ISL_16923329 | EPI_ISL_16923426 | EPI_ISL_16923779 | EPI_ISL_16923869 | EPI_ISL_17104930 |
| EPI_ISL_12030375 | EPI_ISL_16610319 | EPI_ISL_16923330 | EPI_ISL_16923427 | EPI_ISL_16923780 | EPI_ISL_16923870 | EPI_ISL_17261581 |
| EPI_ISL_12046159 | EPI_ISL_16610321 | EPI_ISL_16923331 | EPI_ISL_16923428 | EPI_ISL_16923781 | EPI_ISL_16923871 | EPI_ISL_16923847 |
| EPI_ISL_12046160 | EPI_ISL_16610322 | EPI_ISL_16923332 | EPI_ISL_16923429 | EPI_ISL_16923782 | EPI_ISL_16923873 | EPI_ISL_16923848 |
| EPI_ISL_13858927 | EPI_ISL_16610328 | EPI_ISL_16923333 | EPI_ISL_16923430 | EPI_ISL_16923783 | EPI_ISL_16923874 | EPI_ISL_16923849 |
| EPI_ISL_13858981 | EPI_ISL_16610329 | EPI_ISL_16923334 | EPI_ISL_16923431 | EPI_ISL_16923784 | EPI_ISL_16923875 | EPI_ISL_16923850 |
| EPI_ISL_13858982 | EPI_ISL_16610331 | EPI_ISL_16923335 | EPI_ISL_16923432 | EPI_ISL_16923785 | EPI_ISL_16923876 | EPI_ISL_16923851 |
| EPI_ISL_13858983 | EPI_ISL_16610332 | EPI_ISL_16923336 | EPI_ISL_16923433 | EPI_ISL_16923786 | EPI_ISL_16923877 | EPI_ISL_16923852 |
| EPI_ISL_13858984 | EPI_ISL_16610333 | EPI_ISL_16923337 | EPI_ISL_16923434 | EPI_ISL_16923787 | EPI_ISL_16923878 | EPI_ISL_16923853 |
| EPI_ISL_13858985 | EPI_ISL_16610335 | EPI_ISL_16923338 | EPI_ISL_16923435 | EPI_ISL_16923788 | EPI_ISL_16923879 | EPI_ISL_16923854 |
| EPI_ISL_13858986 | EPI_ISL_16610336 | EPI_ISL_16923339 | EPI_ISL_16923436 | EPI_ISL_16923789 | EPI_ISL_16923880 | EPI_ISL_16923855 |
| EPI_ISL_13858987 | EPI_ISL_16610337 | EPI_ISL_16923343 | EPI_ISL_16923437 | EPI_ISL_16923790 | EPI_ISL_16923881 | EPI_ISL_16923856 |
| EPI_ISL_13858988 | EPI_ISL_16610338 | EPI_ISL_16923344 | EPI_ISL_16923438 | EPI_ISL_16923791 | EPI_ISL_16923882 | EPI_ISL_16923857 |
| EPI_ISL_13858989 | EPI_ISL_16610339 | EPI_ISL_16923345 | EPI_ISL_16923439 | EPI_ISL_16923792 | EPI_ISL_16923883 | EPI_ISL_16923858 |
| EPI_ISL_13858990 | EPI_ISL_16610342 | EPI_ISL_16923346 | EPI_ISL_16923440 | EPI_ISL_16923793 | EPI_ISL_16923884 | EPI_ISL_16923757 |
| EPI_ISL_13858991 | EPI_ISL_16610343 | EPI_ISL_16923347 | EPI_ISL_16923441 | EPI_ISL_16923794 | EPI_ISL_16923885 | EPI_ISL_16923758 |
| EPI_ISL_13858992 | EPI_ISL_16610344 | EPI_ISL_16923348 | EPI_ISL_16923442 | EPI_ISL_16923795 | EPI_ISL_16923886 | EPI_ISL_16923759 |
| EPI_ISL_13858993 | EPI_ISL_16610345 | EPI_ISL_16923349 | EPI_ISL_16923443 | EPI_ISL_16923796 | EPI_ISL_16923887 | EPI_ISL_16923760 |
| EPI_ISL_13858994 | EPI_ISL_16610346 | EPI_ISL_16923354 | EPI_ISL_16923444 | EPI_ISL_16923797 | EPI_ISL_16923888 | EPI_ISL_16923761 |
| EPI_ISL_13858995 | EPI_ISL_16610348 | EPI_ISL_16923355 | EPI_ISL_16923445 | EPI_ISL_16923798 | EPI_ISL_16923889 | EPI_ISL_16923762 |
| EPI_ISL_13858996 | EPI_ISL_16610349 | EPI_ISL_16923356 | EPI_ISL_16923446 | EPI_ISL_16923799 | EPI_ISL_16923890 | EPI_ISL_16923763 |
| EPI_ISL_13858997 | EPI_ISL_16610352 | EPI_ISL_16923357 | EPI_ISL_16923447 | EPI_ISL_16923800 | EPI_ISL_16923891 | EPI_ISL_16923764 |
| EPI_ISL_13858998 | EPI_ISL_16610353 | EPI_ISL_16923358 | EPI_ISL_16923448 | EPI_ISL_16923801 | EPI_ISL_16923892 | EPI_ISL_16923765 |
| EPI_ISL_13858999 | EPI_ISL_16610354 | EPI_ISL_16923359 | EPI_ISL_16923449 | EPI_ISL_16923802 | EPI_ISL_16923893 | EPI_ISL_16923766 |
| EPI_ISL_13859000 | EPI_ISL_16610355 | EPI_ISL_16923360 | EPI_ISL_16923450 | EPI_ISL_16923803 | EPI_ISL_16923894 | EPI_ISL_16923767 |
| EPI_ISL_13859001 | EPI_ISL_16922339 | EPI_ISL_16923361 | EPI_ISL_16923451 | EPI_ISL_16923804 | EPI_ISL_16923895 | EPI_ISL_16923768 |
| EPI_ISL_13859002 | EPI_ISL_16922340 | EPI_ISL_16923362 | EPI_ISL_16923452 | EPI_ISL_16923805 | EPI_ISL_16923896 | EPI_ISL_16923404 |
| EPI_ISL_13859003 | EPI_ISL_16922341 | EPI_ISL_16923363 | EPI_ISL_16923453 | EPI_ISL_16923806 | EPI_ISL_16923897 | EPI_ISL_16923405 |
| EPI_ISL_13859004 | EPI_ISL_16922345 | EPI_ISL_16923364 | EPI_ISL_16923454 | EPI_ISL_16923807 | EPI_ISL_16923898 | EPI_ISL_16923406 |
| EPI_ISL_13859005 | EPI_ISL_16922349 | EPI_ISL_16923365 | EPI_ISL_16923455 | EPI_ISL_16923808 | EPI_ISL_16923899 | EPI_ISL_16923407 |
| EPI_ISL_13859006 | EPI_ISL_16922351 | EPI_ISL_16923366 | EPI_ISL_16923456 | EPI_ISL_16923809 | EPI_ISL_16923900 | EPI_ISL_16923408 |
| EPI_ISL_13859007 | EPI_ISL_16922357 | EPI_ISL_16923367 | EPI_ISL_16923457 | EPI_ISL_16923810 | EPI_ISL_16923901 | EPI_ISL_16923409 |
| EPI_ISL_13859008 | EPI_ISL_16922359 | EPI_ISL_16923368 | EPI_ISL_16923458 | EPI_ISL_16923811 | EPI_ISL_16923902 | EPI_ISL_16923410 |
| EPI_ISL_13859009 | EPI_ISL_16922360 | EPI_ISL_16923369 | EPI_ISL_16923459 | EPI_ISL_16923812 | EPI_ISL_16923903 | EPI_ISL_16923411 |
| EPI_ISL_13859010 | EPI_ISL_16922361 | EPI_ISL_16923370 | EPI_ISL_16923460 | EPI_ISL_16923813 | EPI_ISL_16923904 | EPI_ISL_16923412 |
| EPI_ISL_13859011 | EPI_ISL_16922364 | EPI_ISL_16923371 | EPI_ISL_16923461 | EPI_ISL_16923814 | EPI_ISL_16923905 | EPI_ISL_16923413 |
| EPI_ISL_13859012 | EPI_ISL_16922367 | EPI_ISL_16923372 | EPI_ISL_16923462 | EPI_ISL_16923815 | EPI_ISL_16923906 | EPI_ISL_16923414 |
| EPI_ISL_13859013 | EPI_ISL_16922368 | EPI_ISL_16923373 | EPI_ISL_16923463 | EPI_ISL_16923816 | EPI_ISL_16923907 | EPI_ISL_16923415 |
| EPI_ISL_13859014 | EPI_ISL_16922374 | EPI_ISL_16923374 | EPI_ISL_16923464 | EPI_ISL_16923817 | EPI_ISL_16923908 | EPI_ISL_16923318 |
| EPI_ISL_13859015 | EPI_ISL_16922377 | EPI_ISL_16923375 | EPI_ISL_16923465 | EPI_ISL_16923818 | EPI_ISL_16923909 | EPI_ISL_17261583 |
| EPI_ISL_13859016 | EPI_ISL_16922380 | EPI_ISL_16923376 | EPI_ISL_16923466 | EPI_ISL_16923819 | EPI_ISL_16923910 | EPI_ISL_17261584 |
| EPI_ISL_13859017 | EPI_ISL_16922381 | EPI_ISL_16923377 | EPI_ISL_16923467 | EPI_ISL_16923820 | EPI_ISL_16923911 | EPI_ISL_18070341 |
| EPI_ISL_13859018 | EPI_ISL_16922382 | EPI_ISL_16923378 | EPI_ISL_16923468 | EPI_ISL_16923821 | EPI_ISL_16923912 | EPI_ISL_18070342 |
| EPI_ISL_13859019 | EPI_ISL_16922383 | EPI_ISL_16923379 | EPI_ISL_16923469 | EPI_ISL_16923822 | EPI_ISL_16923913 | EPI_ISL_18070344 |
| EPI_ISL_13859020 | EPI_ISL_16923281 | EPI_ISL_16923380 | EPI_ISL_16923470 | EPI_ISL_16923823 | EPI_ISL_16923914 | EPI_ISL_18070345 |
| EPI_ISL_13859021 | EPI_ISL_16923282 | EPI_ISL_16923381 | EPI_ISL_16923471 | EPI_ISL_16923824 | EPI_ISL_16923915 | EPI_ISL_18070346 |
| EPI_ISL_13859022 | EPI_ISL_16923283 | EPI_ISL_16923382 | EPI_ISL_16923472 | EPI_ISL_16923825 | EPI_ISL_16923916 | EPI_ISL_18070356 |
| EPI_ISL_13859023 | EPI_ISL_16923284 | EPI_ISL_16923383 | EPI_ISL_16923473 | EPI_ISL_16923826 | EPI_ISL_16923917 | EPI_ISL_18070378 |
| EPI_ISL_13859024 | EPI_ISL_16923285 | EPI_ISL_16923384 | EPI_ISL_16923474 | EPI_ISL_16923827 | EPI_ISL_16923918 | EPI_ISL_18070382 |
| EPI_ISL_13859025 | EPI_ISL_16923286 | EPI_ISL_16923385 | EPI_ISL_16923475 | EPI_ISL_16923828 | EPI_ISL_16923919 | EPI_ISL_18070385 |
| EPI_ISL_13859026 | EPI_ISL_16923287 | EPI_ISL_16923386 | EPI_ISL_16923476 | EPI_ISL_16923829 | EPI_ISL_16923920 | EPI_ISL_18070386 |
| EPI_ISL_13859027 | EPI_ISL_16923288 | EPI_ISL_16923387 | EPI_ISL_16923477 | EPI_ISL_16923830 | EPI_ISL_16923921 | EPI_ISL_18070391 |
| EPI_ISL_13859028 | EPI_ISL_16923289 | EPI_ISL_16923388 | EPI_ISL_16923478 | EPI_ISL_16923831 | EPI_ISL_16923922 | EPI_ISL_18070395 |
| EPI_ISL_13859029 | EPI_ISL_16923290 | EPI_ISL_16923389 | EPI_ISL_16923479 | EPI_ISL_16923832 | EPI_ISL_16923923 | EPI_ISL_18070407 |
| EPI_ISL_13859030 | EPI_ISL_16923291 | EPI_ISL_16923390 | EPI_ISL_16923480 | EPI_ISL_16923833 | EPI_ISL_16923924 | EPI_ISL_18070409 |
| EPI_ISL_13859031 | EPI_ISL_16923292 | EPI_ISL_16923391 | EPI_ISL_16923481 | EPI_ISL_16923834 | EPI_ISL_16923925 | EPI_ISL_18070410 |
| EPI_ISL_13859032 | EPI_ISL_16923293 | EPI_ISL_16923392 | EPI_ISL_16923482 | EPI_ISL_16923835 | EPI_ISL_16923927 | EPI_ISL_18070411 |
| EPI_ISL_13859033 | EPI_ISL_16923294 | EPI_ISL_16923393 | EPI_ISL_16923746 | EPI_ISL_16923836 | EPI_ISL_16923928 | EPI_ISL_18070415 |
| EPI_ISL_14770174 | EPI_ISL_16923295 | EPI_ISL_16923394 | EPI_ISL_16923747 | EPI_ISL_16923837 | EPI_ISL_16923929 | EPI_ISL_18070418 |
| EPI_ISL_16610206 | EPI_ISL_16923296 | EPI_ISL_16923395 | EPI_ISL_16923748 | EPI_ISL_16923838 | EPI_ISL_16923930 | EPI_ISL_18070419 |
| EPI_ISL_16610213 | EPI_ISL_16923297 | EPI_ISL_16923396 | EPI_ISL_16923749 | EPI_ISL_16923839 | EPI_ISL_16923931 | EPI_ISL_18070423 |
| EPI_ISL_16610214 | EPI_ISL_16923298 | EPI_ISL_16923397 | EPI_ISL_16923750 | EPI_ISL_16923840 | EPI_ISL_16923932 | EPI_ISL_18070424 |
| EPI_ISL_16610215 | EPI_ISL_16923299 | EPI_ISL_16923398 | EPI_ISL_16923751 | EPI_ISL_16923841 | EPI_ISL_16923933 | EPI_ISL_18070425 |
| EPI_ISL_16610223 | EPI_ISL_16923300 | EPI_ISL_16923399 | EPI_ISL_16923752 | EPI_ISL_16923842 | EPI_ISL_16923934 | EPI_ISL_18070430 |
| EPI_ISL_16610225 | EPI_ISL_16923301 | EPI_ISL_16923400 | EPI_ISL_16923753 | EPI_ISL_16923843 | EPI_ISL_16923935 | EPI_ISL_18070431 |
| EPI_ISL_16610229 | EPI_ISL_16923302 | EPI_ISL_16923401 | EPI_ISL_16923754 | EPI_ISL_16923844 | EPI_ISL_16923936 | EPI_ISL_18070436 |
| EPI_ISL_16610232 | EPI_ISL_16923303 | EPI_ISL_16923402 | EPI_ISL_16923755 | EPI_ISL_16923845 | EPI_ISL_16923937 | EPI_ISL_18070438 |
| EPI_ISL_16610234 | EPI_ISL_16923304 | EPI_ISL_16923403 | EPI_ISL_16923756 | EPI_ISL_16923846 | EPI_ISL_16923938 | EPI_ISL_18435548 |
| EPI_ISL_16610236 | EPI_ISL_16923305 | EPI_ISL_16610245 | EPI_ISL_16610255 | EPI_ISL_16923309 | EPI_ISL_16923312 | EPI_ISL_16923315 |
| EPI_ISL_16610238 | EPI_ISL_16923308 | EPI_ISL_16610246 | EPI_ISL_16610257 | EPI_ISL_16923310 | EPI_ISL_16923313 | EPI_ISL_16923316 |
| EPI_ISL_16610239 | EPI_ISL_16610244 | EPI_ISL_16610248 | EPI_ISL_16610260 | EPI_ISL_16923311 | EPI_ISL_16923314 | EPI_ISL_16923317 |

- Ghana

| EPI_ISL_13392289 | EPI_ISL_13833357 | EPI_ISL_15014133 | EPI_ISL_15280402 | EPI_ISL_15280448 | EPI_ISL_15781094 | EPI_ISL_15781102 |
| --- | --- | --- | --- | --- | --- | --- |
| EPI_ISL_13392293 | EPI_ISL_13833358 | EPI_ISL_15187041 | EPI_ISL_15280409 | EPI_ISL_15781085 | EPI_ISL_15781096 | EPI_ISL_15781103 |
| EPI_ISL_13392322 | EPI_ISL_13833375 | EPI_ISL_15280392 | EPI_ISL_15280443 | EPI_ISL_15781087 | EPI_ISL_15781097 | EPI_ISL_15781108 |
| EPI_ISL_13392352 | EPI_ISL_15014118 | EPI_ISL_15280393 | EPI_ISL_15280444 | EPI_ISL_15781089 | EPI_ISL_15781098 | EPI_ISL_15887118 |
| EPI_ISL_13393947 | EPI_ISL_15014128 | EPI_ISL_15280401 | EPI_ISL_15280447 | EPI_ISL_15781090 | EPI_ISL_15781099 | EPI_ISL_15887119 |
| EPI_ISL_13833324 | EPI_ISL_15887124 | EPI_ISL_15887129 | EPI_ISL_16275891 | EPI_ISL_15781093 | EPI_ISL_15781101 |  |

- England

| EPI_ISL_9991425 | EPI_ISL_10049698 | EPI_ISL_10051812 | EPI_ISL_10053389 | EPI_ISL_10105967 | EPI_ISL_10107271 | EPI_ISL_10108145 |
| --- | --- | --- | --- | --- | --- | --- |
| EPI_ISL_9991440 | EPI_ISL_10049703 | EPI_ISL_10051822 | EPI_ISL_10053391 | EPI_ISL_10105974 | EPI_ISL_10107278 | EPI_ISL_10108149 |
| EPI_ISL_9991450 | EPI_ISL_10049715 | EPI_ISL_10051840 | EPI_ISL_10053396 | EPI_ISL_10105982 | EPI_ISL_10107287 | EPI_ISL_10108150 |
| EPI_ISL_9991471 | EPI_ISL_10049757 | EPI_ISL_10051842 | EPI_ISL_10053397 | EPI_ISL_10105989 | EPI_ISL_10107288 | EPI_ISL_10108158 |
| EPI_ISL_9991501 | EPI_ISL_10049759 | EPI_ISL_10051877 | EPI_ISL_10053398 | EPI_ISL_10105997 | EPI_ISL_10107290 | EPI_ISL_10108160 |
| EPI_ISL_9991502 | EPI_ISL_10049766 | EPI_ISL_10052386 | EPI_ISL_10053411 | EPI_ISL_10106010 | EPI_ISL_10107291 | EPI_ISL_10108164 |
| EPI_ISL_9991509 | EPI_ISL_10049814 | EPI_ISL_10052407 | EPI_ISL_10053413 | EPI_ISL_10106018 | EPI_ISL_10107306 | EPI_ISL_10108183 |
| EPI_ISL_9991544 | EPI_ISL_10049842 | EPI_ISL_10052411 | EPI_ISL_10053416 | EPI_ISL_10106019 | EPI_ISL_10107371 | EPI_ISL_10108253 |
| EPI_ISL_9991552 | EPI_ISL_10049867 | EPI_ISL_10052468 | EPI_ISL_10053422 | EPI_ISL_10106032 | EPI_ISL_10107400 | EPI_ISL_10108256 |
| EPI_ISL_9991577 | EPI_ISL_10049875 | EPI_ISL_10052495 | EPI_ISL_10053429 | EPI_ISL_10106039 | EPI_ISL_10107589 | EPI_ISL_10108291 |
| EPI_ISL_9991580 | EPI_ISL_10049879 | EPI_ISL_10052513 | EPI_ISL_10053430 | EPI_ISL_10106049 | EPI_ISL_10107652 | EPI_ISL_10108656 |
| EPI_ISL_9991593 | EPI_ISL_10049981 | EPI_ISL_10052517 | EPI_ISL_10053434 | EPI_ISL_10106055 | EPI_ISL_10107655 | EPI_ISL_10108657 |
| EPI_ISL_9991600 | EPI_ISL_10049989 | EPI_ISL_10052523 | EPI_ISL_10053436 | EPI_ISL_10106172 | EPI_ISL_10107676 | EPI_ISL_10108659 |
| EPI_ISL_9991605 | EPI_ISL_10050005 | EPI_ISL_10052557 | EPI_ISL_10053442 | EPI_ISL_10106362 | EPI_ISL_10107680 | EPI_ISL_10108674 |
| EPI_ISL_9991609 | EPI_ISL_10050028 | EPI_ISL_10052580 | EPI_ISL_10053445 | EPI_ISL_10106373 | EPI_ISL_10107681 | EPI_ISL_10108679 |
| EPI_ISL_9991613 | EPI_ISL_10050033 | EPI_ISL_10052605 | EPI_ISL_10053459 | EPI_ISL_10106396 | EPI_ISL_10107736 | EPI_ISL_10108689 |
| EPI_ISL_9991614 | EPI_ISL_10050053 | EPI_ISL_10052669 | EPI_ISL_10053467 | EPI_ISL_10106403 | EPI_ISL_10107741 | EPI_ISL_10108690 |
| EPI_ISL_9991615 | EPI_ISL_10050061 | EPI_ISL_10052690 | EPI_ISL_10053485 | EPI_ISL_10106408 | EPI_ISL_10107744 | EPI_ISL_10108696 |
| EPI_ISL_9991626 | EPI_ISL_10050074 | EPI_ISL_10052697 | EPI_ISL_10053488 | EPI_ISL_10106428 | EPI_ISL_10107747 | EPI_ISL_10108704 |
| EPI_ISL_9991633 | EPI_ISL_10050081 | EPI_ISL_10052698 | EPI_ISL_10053503 | EPI_ISL_10106457 | EPI_ISL_10107773 | EPI_ISL_10108705 |
| EPI_ISL_9991636 | EPI_ISL_10050089 | EPI_ISL_10052718 | EPI_ISL_10053505 | EPI_ISL_10106486 | EPI_ISL_10107788 | EPI_ISL_10108708 |
| EPI_ISL_9991638 | EPI_ISL_10050132 | EPI_ISL_10052748 | EPI_ISL_10053507 | EPI_ISL_10106528 | EPI_ISL_10107796 | EPI_ISL_10108710 |
| EPI_ISL_9991651 | EPI_ISL_10051088 | EPI_ISL_10052754 | EPI_ISL_10053512 | EPI_ISL_10106538 | EPI_ISL_10107848 | EPI_ISL_10108716 |
| EPI_ISL_9991676 | EPI_ISL_10051091 | EPI_ISL_10052755 | EPI_ISL_10053527 | EPI_ISL_10106549 | EPI_ISL_10107851 | EPI_ISL_10108717 |
| EPI_ISL_9991685 | EPI_ISL_10051107 | EPI_ISL_10052777 | EPI_ISL_10053536 | EPI_ISL_10106556 | EPI_ISL_10107873 | EPI_ISL_10108722 |
| EPI_ISL_9991695 | EPI_ISL_10051115 | EPI_ISL_10052811 | EPI_ISL_10053539 | EPI_ISL_10106606 | EPI_ISL_10107888 | EPI_ISL_10108724 |
| EPI_ISL_9991700 | EPI_ISL_10051117 | EPI_ISL_10052841 | EPI_ISL_10053540 | EPI_ISL_10106607 | EPI_ISL_10107896 | EPI_ISL_10108726 |
| EPI_ISL_9991705 | EPI_ISL_10051136 | EPI_ISL_10052842 | EPI_ISL_10053549 | EPI_ISL_10106608 | EPI_ISL_10107903 | EPI_ISL_10108728 |
| EPI_ISL_9991731 | EPI_ISL_10051137 | EPI_ISL_10052968 | EPI_ISL_10053583 | EPI_ISL_10106625 | EPI_ISL_10107915 | EPI_ISL_10108734 |
| EPI_ISL_9991745 | EPI_ISL_10051138 | EPI_ISL_10052987 | EPI_ISL_10053587 | EPI_ISL_10106633 | EPI_ISL_10107916 | EPI_ISL_10108736 |
| EPI_ISL_9991749 | EPI_ISL_10051144 | EPI_ISL_10053042 | EPI_ISL_10053590 | EPI_ISL_10106653 | EPI_ISL_10107939 | EPI_ISL_10108738 |
| EPI_ISL_9991751 | EPI_ISL_10051158 | EPI_ISL_10053099 | EPI_ISL_10053596 | EPI_ISL_10106655 | EPI_ISL_10107959 | EPI_ISL_10108741 |
| EPI_ISL_9991766 | EPI_ISL_10051159 | EPI_ISL_10053113 | EPI_ISL_10053604 | EPI_ISL_10106657 | EPI_ISL_10107962 | EPI_ISL_10108743 |
| EPI_ISL_9991794 | EPI_ISL_10051160 | EPI_ISL_10053138 | EPI_ISL_10053605 | EPI_ISL_10106675 | EPI_ISL_10107968 | EPI_ISL_10108757 |
| EPI_ISL_9991802 | EPI_ISL_10051170 | EPI_ISL_10053146 | EPI_ISL_10053610 | EPI_ISL_10106679 | EPI_ISL_10107969 | EPI_ISL_10108764 |
| EPI_ISL_9991807 | EPI_ISL_10051175 | EPI_ISL_10053163 | EPI_ISL_10053645 | EPI_ISL_10106681 | EPI_ISL_10107981 | EPI_ISL_10108766 |
| EPI_ISL_9991808 | EPI_ISL_10051178 | EPI_ISL_10053185 | EPI_ISL_10053655 | EPI_ISL_10106707 | EPI_ISL_10107986 | EPI_ISL_10108794 |
| EPI_ISL_9991818 | EPI_ISL_10051179 | EPI_ISL_10053238 | EPI_ISL_10053667 | EPI_ISL_10106859 | EPI_ISL_10107991 | EPI_ISL_10108799 |
| EPI_ISL_9991820 | EPI_ISL_10051184 | EPI_ISL_10053244 | EPI_ISL_10053671 | EPI_ISL_10106863 | EPI_ISL_10107996 | EPI_ISL_10108809 |
| EPI_ISL_9991824 | EPI_ISL_10051195 | EPI_ISL_10053251 | EPI_ISL_10053679 | EPI_ISL_10106866 | EPI_ISL_10108002 | EPI_ISL_10108817 |
| EPI_ISL_9991834 | EPI_ISL_10051197 | EPI_ISL_10053252 | EPI_ISL_10053683 | EPI_ISL_10106874 | EPI_ISL_10108003 | EPI_ISL_10108830 |
| EPI_ISL_9991859 | EPI_ISL_10051201 | EPI_ISL_10053255 | EPI_ISL_10053726 | EPI_ISL_10106877 | EPI_ISL_10108005 | EPI_ISL_10108831 |
| EPI_ISL_9991861 | EPI_ISL_10051206 | EPI_ISL_10053257 | EPI_ISL_10053741 | EPI_ISL_10106879 | EPI_ISL_10108008 | EPI_ISL_10108842 |
| EPI_ISL_9991874 | EPI_ISL_10051207 | EPI_ISL_10053260 | EPI_ISL_10053752 | EPI_ISL_10106884 | EPI_ISL_10108011 | EPI_ISL_10108846 |
| EPI_ISL_9991876 | EPI_ISL_10051214 | EPI_ISL_10053262 | EPI_ISL_10053789 | EPI_ISL_10106889 | EPI_ISL_10108020 | EPI_ISL_10108848 |
| EPI_ISL_9991889 | EPI_ISL_10051219 | EPI_ISL_10053270 | EPI_ISL_10053801 | EPI_ISL_10106900 | EPI_ISL_10108022 | EPI_ISL_10108853 |
| EPI_ISL_9991896 | EPI_ISL_10051237 | EPI_ISL_10053272 | EPI_ISL_10053811 | EPI_ISL_10106904 | EPI_ISL_10108027 | EPI_ISL_10108859 |
| EPI_ISL_9991900 | EPI_ISL_10051239 | EPI_ISL_10053287 | EPI_ISL_10053816 | EPI_ISL_10106911 | EPI_ISL_10108028 | EPI_ISL_10108860 |
| EPI_ISL_9991948 | EPI_ISL_10051241 | EPI_ISL_10053291 | EPI_ISL_10053819 | EPI_ISL_10106922 | EPI_ISL_10108031 | EPI_ISL_10108866 |
| EPI_ISL_9991988 | EPI_ISL_10051246 | EPI_ISL_10053295 | EPI_ISL_10054109 | EPI_ISL_10106927 | EPI_ISL_10108032 | EPI_ISL_10108867 |
| EPI_ISL_9991995 | EPI_ISL_10051250 | EPI_ISL_10053300 | EPI_ISL_10054112 | EPI_ISL_10106933 | EPI_ISL_10108034 | EPI_ISL_10108872 |
| EPI_ISL_9992017 | EPI_ISL_10051253 | EPI_ISL_10053307 | EPI_ISL_10054128 | EPI_ISL_10106944 | EPI_ISL_10108042 | EPI_ISL_10108873 |
| EPI_ISL_9992020 | EPI_ISL_10051267 | EPI_ISL_10053321 | EPI_ISL_10054138 | EPI_ISL_10106954 | EPI_ISL_10108047 | EPI_ISL_10108874 |
| EPI_ISL_9992025 | EPI_ISL_10051270 | EPI_ISL_10053329 | EPI_ISL_10054166 | EPI_ISL_10106965 | EPI_ISL_10108054 | EPI_ISL_10108876 |
| EPI_ISL_9992032 | EPI_ISL_10051277 | EPI_ISL_10053338 | EPI_ISL_10054198 | EPI_ISL_10106975 | EPI_ISL_10108056 | EPI_ISL_10108878 |
| EPI_ISL_9992046 | EPI_ISL_10051304 | EPI_ISL_10053348 | EPI_ISL_10054199 | EPI_ISL_10106984 | EPI_ISL_10108058 | EPI_ISL_10108880 |
| EPI_ISL_9992051 | EPI_ISL_10051306 | EPI_ISL_10053350 | EPI_ISL_10054260 | EPI_ISL_10106986 | EPI_ISL_10108060 | EPI_ISL_10108899 |
| EPI_ISL_9992056 | EPI_ISL_10051315 | EPI_ISL_10053353 | EPI_ISL_10054272 | EPI_ISL_10106989 | EPI_ISL_10108065 | EPI_ISL_10108907 |
| EPI_ISL_9992085 | EPI_ISL_10051321 | EPI_ISL_10053358 | EPI_ISL_10054281 | EPI_ISL_10106993 | EPI_ISL_10108068 | EPI_ISL_10108908 |
| EPI_ISL_9992089 | EPI_ISL_10051325 | EPI_ISL_10053361 | EPI_ISL_10054338 | EPI_ISL_10106997 | EPI_ISL_10108071 | EPI_ISL_10108914 |
| EPI_ISL_9992096 | EPI_ISL_10051326 | EPI_ISL_10053363 | EPI_ISL_10054341 | EPI_ISL_10107014 | EPI_ISL_10108075 | EPI_ISL_10108115 |
| EPI_ISL_9992106 | EPI_ISL_10051335 | EPI_ISL_10053365 | EPI_ISL_10054369 | EPI_ISL_10107018 | EPI_ISL_10108076 | EPI_ISL_10108123 |
| EPI_ISL_9992114 | EPI_ISL_10051343 | EPI_ISL_10053376 | EPI_ISL_10054391 | EPI_ISL_10107019 | EPI_ISL_10108078 | EPI_ISL_10108124 |
| EPI_ISL_9992116 | EPI_ISL_10051381 | EPI_ISL_10053386 | EPI_ISL_10054397 | EPI_ISL_10107031 | EPI_ISL_10108086 | EPI_ISL_10108132 |
| EPI_ISL_10048678 | EPI_ISL_10051403 | EPI_ISL_10107119 | EPI_ISL_10054402 | EPI_ISL_10107034 | EPI_ISL_10108106 | EPI_ISL_10108136 |
| EPI_ISL_10048731 | EPI_ISL_10051429 | EPI_ISL_10107121 | EPI_ISL_10054404 | EPI_ISL_10107035 | EPI_ISL_10108109 | EPI_ISL_10108142 |
| EPI_ISL_10048816 | EPI_ISL_10051460 | EPI_ISL_10107133 | EPI_ISL_10054417 | EPI_ISL_10107038 | EPI_ISL_10108110 | EPI_ISL_10108143 |
| EPI_ISL_10048925 | EPI_ISL_10051474 | EPI_ISL_10107145 | EPI_ISL_10054420 | EPI_ISL_10107050 | EPI_ISL_10054502 | EPI_ISL_10107080 |
| EPI_ISL_10049013 | EPI_ISL_10051580 | EPI_ISL_10107147 | EPI_ISL_10054440 | EPI_ISL_10107053 | EPI_ISL_10054521 | EPI_ISL_10107088 |
| EPI_ISL_10049028 | EPI_ISL_10051593 | EPI_ISL_10107169 | EPI_ISL_10054444 | EPI_ISL_10107057 | EPI_ISL_10054532 | EPI_ISL_10107090 |
| EPI_ISL_10049063 | EPI_ISL_10051628 | EPI_ISL_10107187 | EPI_ISL_10054480 | EPI_ISL_10107073 | EPI_ISL_10054542 | EPI_ISL_10107094 |
| EPI_ISL_10049078 | EPI_ISL_10051636 | EPI_ISL_10107201 | EPI_ISL_10054481 | EPI_ISL_10107074 | EPI_ISL_10054549 | EPI_ISL_10107103 |
| EPI_ISL_10049130 | EPI_ISL_10051647 | EPI_ISL_10107212 | EPI_ISL_10054487 | EPI_ISL_10107075 | EPI_ISL_10049588 | EPI_ISL_10049473 |
| EPI_ISL_10049202 | EPI_ISL_10051746 | EPI_ISL_10107227 | EPI_ISL_10051770 | EPI_ISL_10107253 | EPI_ISL_10049623 | EPI_ISL_10049483 |
| EPI_ISL_10049321 | EPI_ISL_10051763 | EPI_ISL_10107229 | EPI_ISL_10051787 | EPI_ISL_10107264 | EPI_ISL_10049638 | EPI_ISL_10049573 |
| EPI_ISL_10049341 | EPI_ISL_10049467 | EPI_ISL_10107240 | EPI_ISL_10051791 | EPI_ISL_10107268 | EPI_ISL_10049681 | EPI_ISL_10049585 |

- USA

| EPI_ISL_10075517 | EPI_ISL_10371845 | EPI_ISL_10471775 | EPI_ISL_10567151 | EPI_ISL_10713291 | EPI_ISL_10832861 | EPI_ISL_11134395 |
| --- | --- | --- | --- | --- | --- | --- |
| EPI_ISL_10080567 | EPI_ISL_10371859 | EPI_ISL_10472183 | EPI_ISL_10567153 | EPI_ISL_10713368 | EPI_ISL_10832863 | EPI_ISL_11134520 |
| EPI_ISL_10103561 | EPI_ISL_10374582 | EPI_ISL_10472192 | EPI_ISL_10567190 | EPI_ISL_10713418 | EPI_ISL_10832866 | EPI_ISL_11178998 |
| EPI_ISL_10103585 | EPI_ISL_10374584 | EPI_ISL_10472451 | EPI_ISL_10567211 | EPI_ISL_10713544 | EPI_ISL_10832870 | EPI_ISL_11206732 |
| EPI_ISL_10103650 | EPI_ISL_10374586 | EPI_ISL_10480154 | EPI_ISL_10567232 | EPI_ISL_10713675 | EPI_ISL_10832874 | EPI_ISL_11247266 |
| EPI_ISL_10103655 | EPI_ISL_10374587 | EPI_ISL_10480920 | EPI_ISL_10567258 | EPI_ISL_10713773 | EPI_ISL_10832880 | EPI_ISL_11247290 |
| EPI_ISL_10103658 | EPI_ISL_10377160 | EPI_ISL_10481017 | EPI_ISL_10567266 | EPI_ISL_10713911 | EPI_ISL_10834286 | EPI_ISL_11247413 |
| EPI_ISL_10131999 | EPI_ISL_10377378 | EPI_ISL_10481662 | EPI_ISL_10567279 | EPI_ISL_10714094 | EPI_ISL_10834301 | EPI_ISL_11247417 |
| EPI_ISL_10132035 | EPI_ISL_10377386 | EPI_ISL_10481698 | EPI_ISL_10567319 | EPI_ISL_10714152 | EPI_ISL_10834330 | EPI_ISL_11247437 |
| EPI_ISL_10132062 | EPI_ISL_10377409 | EPI_ISL_10481709 | EPI_ISL_10567336 | EPI_ISL_10714245 | EPI_ISL_10834339 | EPI_ISL_11247458 |
| EPI_ISL_10132132 | EPI_ISL_10377628 | EPI_ISL_10481861 | EPI_ISL_10567368 | EPI_ISL_10714253 | EPI_ISL_10839552 | EPI_ISL_11293593 |
| EPI_ISL_10132234 | EPI_ISL_10378061 | EPI_ISL_10482392 | EPI_ISL_10567372 | EPI_ISL_10714263 | EPI_ISL_10839599 | EPI_ISL_11293739 |
| EPI_ISL_10132238 | EPI_ISL_10381177 | EPI_ISL_10482911 | EPI_ISL_10567418 | EPI_ISL_10714266 | EPI_ISL_10839668 | EPI_ISL_11293743 |
| EPI_ISL_10132299 | EPI_ISL_10388138 | EPI_ISL_10483385 | EPI_ISL_10567419 | EPI_ISL_10714270 | EPI_ISL_10839733 | EPI_ISL_11295157 |
| EPI_ISL_10183741 | EPI_ISL_10388355 | EPI_ISL_10483839 | EPI_ISL_10567467 | EPI_ISL_10714275 | EPI_ISL_10839755 | EPI_ISL_11356623 |
| EPI_ISL_10233303 | EPI_ISL_10388463 | EPI_ISL_10484222 | EPI_ISL_10567468 | EPI_ISL_10714279 | EPI_ISL_10839815 | EPI_ISL_11356682 |
| EPI_ISL_10233362 | EPI_ISL_10388515 | EPI_ISL_10484744 | EPI_ISL_10567477 | EPI_ISL_10714289 | EPI_ISL_10839816 | EPI_ISL_11357216 |
| EPI_ISL_10279661 | EPI_ISL_10388672 | EPI_ISL_10484810 | EPI_ISL_10567544 | EPI_ISL_10714290 | EPI_ISL_10865999 | EPI_ISL_11357218 |
| EPI_ISL_10307636 | EPI_ISL_10388925 | EPI_ISL_10484954 | EPI_ISL_10567549 | EPI_ISL_10714291 | EPI_ISL_10870465 | EPI_ISL_11357228 |
| EPI_ISL_10307697 | EPI_ISL_10388935 | EPI_ISL_10490508 | EPI_ISL_10567579 | EPI_ISL_10714294 | EPI_ISL_10870467 | EPI_ISL_11357364 |
| EPI_ISL_10307718 | EPI_ISL_10388985 | EPI_ISL_10490600 | EPI_ISL_10567599 | EPI_ISL_10714298 | EPI_ISL_10870468 | EPI_ISL_11358237 |
| EPI_ISL_10307807 | EPI_ISL_10389031 | EPI_ISL_10490703 | EPI_ISL_10567701 | EPI_ISL_10714300 | EPI_ISL_10870471 | EPI_ISL_11378804 |
| EPI_ISL_10308969 | EPI_ISL_10389375 | EPI_ISL_10490747 | EPI_ISL_10567750 | EPI_ISL_10714301 | EPI_ISL_10870472 | EPI_ISL_11414293 |
| EPI_ISL_10309945 | EPI_ISL_10389737 | EPI_ISL_10490845 | EPI_ISL_10567764 | EPI_ISL_10714302 | EPI_ISL_10874409 | EPI_ISL_11414297 |
| EPI_ISL_10309957 | EPI_ISL_10389760 | EPI_ISL_10490885 | EPI_ISL_10567769 | EPI_ISL_10714303 | EPI_ISL_10892661 | EPI_ISL_11487076 |
| EPI_ISL_10310006 | EPI_ISL_10389914 | EPI_ISL_10490889 | EPI_ISL_10567798 | EPI_ISL_10714304 | EPI_ISL_10892738 | EPI_ISL_11531897 |
| EPI_ISL_10310161 | EPI_ISL_10390211 | EPI_ISL_10490954 | EPI_ISL_10567800 | EPI_ISL_10714306 | EPI_ISL_10892739 | EPI_ISL_11573984 |
| EPI_ISL_10310171 | EPI_ISL_10390225 | EPI_ISL_10491016 | EPI_ISL_10567917 | EPI_ISL_10714307 | EPI_ISL_10896218 | EPI_ISL_11134146 |
| EPI_ISL_10310174 | EPI_ISL_10392578 | EPI_ISL_10491082 | EPI_ISL_10595498 | EPI_ISL_10714308 | EPI_ISL_10903316 | EPI_ISL_11134258 |
| EPI_ISL_10310362 | EPI_ISL_10392580 | EPI_ISL_10491245 | EPI_ISL_10595505 | EPI_ISL_10714335 | EPI_ISL_10903411 | EPI_ISL_11574009 |
| EPI_ISL_10310388 | EPI_ISL_10392581 | EPI_ISL_10491251 | EPI_ISL_10596444 | EPI_ISL_10714398 | EPI_ISL_10907106 | EPI_ISL_11626828 |
| EPI_ISL_10310572 | EPI_ISL_10397664 | EPI_ISL_10491267 | EPI_ISL_10596722 | EPI_ISL_10714418 | EPI_ISL_10957745 | EPI_ISL_11673573 |
| EPI_ISL_10310790 | EPI_ISL_10430714 | EPI_ISL_10491283 | EPI_ISL_10596930 | EPI_ISL_10715064 | EPI_ISL_10972679 | EPI_ISL_11673575 |
| EPI_ISL_10310904 | EPI_ISL_10430715 | EPI_ISL_10491308 | EPI_ISL_10597059 | EPI_ISL_10715514 | EPI_ISL_10979520 | EPI_ISL_11673602 |
| EPI_ISL_10310979 | EPI_ISL_10430716 | EPI_ISL_10491441 | EPI_ISL_10597372 | EPI_ISL_10715608 | EPI_ISL_10979681 | EPI_ISL_11673717 |
| EPI_ISL_10311109 | EPI_ISL_10430885 | EPI_ISL_10492321 | EPI_ISL_10597439 | EPI_ISL_10715631 | EPI_ISL_10980306 | EPI_ISL_11673737 |
| EPI_ISL_10311141 | EPI_ISL_10431274 | EPI_ISL_10492325 | EPI_ISL_10597913 | EPI_ISL_10715912 | EPI_ISL_11012824 | EPI_ISL_11673763 |
| EPI_ISL_10311156 | EPI_ISL_10431303 | EPI_ISL_10492405 | EPI_ISL_10604521 | EPI_ISL_10715947 | EPI_ISL_11012843 | EPI_ISL_11673764 |
| EPI_ISL_10311162 | EPI_ISL_10431325 | EPI_ISL_10492415 | EPI_ISL_10604530 | EPI_ISL_10716271 | EPI_ISL_11013323 | EPI_ISL_11673839 |
| EPI_ISL_10311246 | EPI_ISL_10431333 | EPI_ISL_10492417 | EPI_ISL_10620321 | EPI_ISL_10723376 | EPI_ISL_11014955 | EPI_ISL_11673851 |
| EPI_ISL_10311268 | EPI_ISL_10431340 | EPI_ISL_10492472 | EPI_ISL_10620324 | EPI_ISL_10723434 | EPI_ISL_11014956 | EPI_ISL_11673885 |
| EPI_ISL_10311283 | EPI_ISL_10431365 | EPI_ISL_10492474 | EPI_ISL_10620325 | EPI_ISL_10761186 | EPI_ISL_11014964 | EPI_ISL_11797511 |
| EPI_ISL_10311377 | EPI_ISL_10431391 | EPI_ISL_10492475 | EPI_ISL_10620326 | EPI_ISL_10761192 | EPI_ISL_11032159 | EPI_ISL_11797519 |
| EPI_ISL_10311380 | EPI_ISL_10431399 | EPI_ISL_10492550 | EPI_ISL_10620327 | EPI_ISL_10761193 | EPI_ISL_11032193 | EPI_ISL_11797520 |
| EPI_ISL_10311427 | EPI_ISL_10431474 | EPI_ISL_10492566 | EPI_ISL_10620538 | EPI_ISL_10761195 | EPI_ISL_11134129 | EPI_ISL_11797522 |
| EPI_ISL_10311451 | EPI_ISL_10431486 | EPI_ISL_10492574 | EPI_ISL_10623672 | EPI_ISL_10761212 | EPI_ISL_11134144 | EPI_ISL_11893394 |
| EPI_ISL_10311468 | EPI_ISL_10431491 | EPI_ISL_10539571 | EPI_ISL_10624521 | EPI_ISL_10761215 | EPI_ISL_10791210 | EPI_ISL_11893522 |
| EPI_ISL_10311523 | EPI_ISL_10431596 | EPI_ISL_10549590 | EPI_ISL_10624576 | EPI_ISL_10761216 | EPI_ISL_10832416 | EPI_ISL_12147415 |
| EPI_ISL_10311546 | EPI_ISL_10431623 | EPI_ISL_10550941 | EPI_ISL_10624579 | EPI_ISL_10761222 | EPI_ISL_10832417 | EPI_ISL_12275271 |
| EPI_ISL_10311631 | EPI_ISL_10431646 | EPI_ISL_10550946 | EPI_ISL_10624633 | EPI_ISL_10761231 | EPI_ISL_10832418 | EPI_ISL_12415178 |
| EPI_ISL_10311653 | EPI_ISL_10431677 | EPI_ISL_10557942 | EPI_ISL_10624791 | EPI_ISL_10761264 | EPI_ISL_10832419 | EPI_ISL_12415179 |
| EPI_ISL_10311655 | EPI_ISL_10431710 | EPI_ISL_10557973 | EPI_ISL_10624837 | EPI_ISL_10771186 | EPI_ISL_10832420 | EPI_ISL_12470728 |
| EPI_ISL_10311659 | EPI_ISL_10431712 | EPI_ISL_10558000 | EPI_ISL_10624985 | EPI_ISL_10712845 | EPI_ISL_10832421 | EPI_ISL_12470731 |
| EPI_ISL_10311662 | EPI_ISL_10431719 | EPI_ISL_10558016 | EPI_ISL_10625879 | EPI_ISL_10561718 | EPI_ISL_10832422 | EPI_ISL_12615660 |
| EPI_ISL_10311664 | EPI_ISL_10431723 | EPI_ISL_10558044 | EPI_ISL_10635426 | EPI_ISL_10561733 | EPI_ISL_10832424 | EPI_ISL_12615661 |
| EPI_ISL_10311667 | EPI_ISL_10431730 | EPI_ISL_10558094 | EPI_ISL_10636340 | EPI_ISL_10561768 | EPI_ISL_10832425 | EPI_ISL_12693365 |
| EPI_ISL_10311669 | EPI_ISL_10431734 | EPI_ISL_10558107 | EPI_ISL_10646704 | EPI_ISL_10562248 | EPI_ISL_10832427 | EPI_ISL_12718060 |
| EPI_ISL_10311671 | EPI_ISL_10431794 | EPI_ISL_10558159 | EPI_ISL_10650695 | EPI_ISL_10566530 | EPI_ISL_10832428 | EPI_ISL_12718065 |
| EPI_ISL_10311673 | EPI_ISL_10431795 | EPI_ISL_10558324 | EPI_ISL_10654431 | EPI_ISL_10566557 | EPI_ISL_10832430 | EPI_ISL_12800285 |
| EPI_ISL_10311675 | EPI_ISL_10431843 | EPI_ISL_10558828 | EPI_ISL_10654453 | EPI_ISL_10566570 | EPI_ISL_10832432 | EPI_ISL_12921883 |
| EPI_ISL_10311680 | EPI_ISL_10431854 | EPI_ISL_10559079 | EPI_ISL_10686509 | EPI_ISL_10566602 | EPI_ISL_10832761 | EPI_ISL_12921965 |
| EPI_ISL_10311682 | EPI_ISL_10431855 | EPI_ISL_10559095 | EPI_ISL_10695391 | EPI_ISL_10566705 | EPI_ISL_10832765 | EPI_ISL_12921968 |
| EPI_ISL_10311684 | EPI_ISL_10461229 | EPI_ISL_10559779 | EPI_ISL_10704127 | EPI_ISL_10566733 | EPI_ISL_10832768 | EPI_ISL_12921970 |
| EPI_ISL_10311687 | EPI_ISL_10461742 | EPI_ISL_10559797 | EPI_ISL_10707356 | EPI_ISL_10566812 | EPI_ISL_10832777 | EPI_ISL_12921972 |
| EPI_ISL_10311689 | EPI_ISL_10461870 | EPI_ISL_10559812 | EPI_ISL_10707357 | EPI_ISL_10566824 | EPI_ISL_10832794 | EPI_ISL_12921974 |
| EPI_ISL_10311692 | EPI_ISL_10462279 | EPI_ISL_10559848 | EPI_ISL_10707358 | EPI_ISL_10566844 | EPI_ISL_10832798 | EPI_ISL_12922295 |
| EPI_ISL_10311694 | EPI_ISL_10462327 | EPI_ISL_10559851 | EPI_ISL_10708048 | EPI_ISL_10566845 | EPI_ISL_10832802 | EPI_ISL_12922339 |
| EPI_ISL_10311697 | EPI_ISL_10462738 | EPI_ISL_10559856 | EPI_ISL_10708049 | EPI_ISL_10566863 | EPI_ISL_10832835 | EPI_ISL_12922380 |
| EPI_ISL_10311699 | EPI_ISL_10462750 | EPI_ISL_10559940 | EPI_ISL_10708050 | EPI_ISL_10566896 | EPI_ISL_10832836 | EPI_ISL_12922396 |
| EPI_ISL_10311733 | EPI_ISL_10463475 | EPI_ISL_10560010 | EPI_ISL_10708052 | EPI_ISL_10566908 | EPI_ISL_10832849 | EPI_ISL_13295489 |
| EPI_ISL_10311813 | EPI_ISL_10463955 | EPI_ISL_10560068 | EPI_ISL_10708053 | EPI_ISL_10566924 | EPI_ISL_10832855 | EPI_ISL_13374358 |
| EPI_ISL_10311815 | EPI_ISL_10463979 | EPI_ISL_10560171 | EPI_ISL_10708054 | EPI_ISL_10567045 | EPI_ISL_10771283 | EPI_ISL_13797119 |
| EPI_ISL_10311816 | EPI_ISL_10463983 | EPI_ISL_10560408 | EPI_ISL_10708055 | EPI_ISL_10467209 | EPI_ISL_10771391 | EPI_ISL_14723082 |
| EPI_ISL_10311821 | EPI_ISL_10464460 | EPI_ISL_10560868 | EPI_ISL_10708056 | EPI_ISL_10467533 | EPI_ISL_10777834 | EPI_ISL_15047138 |
| EPI_ISL_10311825 | EPI_ISL_10464499 | EPI_ISL_10560924 | EPI_ISL_10708057 | EPI_ISL_10467542 | EPI_ISL_10778001 | EPI_ISL_16844619 |
| EPI_ISL_10311834 | EPI_ISL_10465023 | EPI_ISL_10560962 | EPI_ISL_10708059 | EPI_ISL_10468488 | EPI_ISL_10780889 | EPI_ISL_17125159 |
| EPI_ISL_10311877 | EPI_ISL_10465879 | EPI_ISL_10561083 | EPI_ISL_10708060 | EPI_ISL_10468554 | EPI_ISL_10781712 | EPI_ISL_17132875 |
| EPI_ISL_10311888 | EPI_ISL_10466360 | EPI_ISL_10561205 | EPI_ISL_10708061 | EPI_ISL_10468559 | EPI_ISL_10782036 | EPI_ISL_17132892 |
| EPI_ISL_10311937 | EPI_ISL_10466451 | EPI_ISL_10561340 | EPI_ISL_10708065 | EPI_ISL_10470928 | EPI_ISL_10789727 | EPI_ISL_17546523 |
| EPI_ISL_10322745 | EPI_ISL_10466803 | EPI_ISL_10561378 | EPI_ISL_10708066 | EPI_ISL_10471209 | EPI_ISL_10791197 | EPI_ISL_17576155 |
| EPI_ISL_10322748 | EPI_ISL_10466932 | EPI_ISL_10561390 | EPI_ISL_10708067 | EPI_ISL_10471219 | EPI_ISL_10771199 | EPI_ISL_17845835 |
| EPI_ISL_10322749 | EPI_ISL_10467075 | EPI_ISL_10561463 | EPI_ISL_10708068 | EPI_ISL_10471286 | EPI_ISL_10771200 | EPI_ISL_17846224 |
| EPI_ISL_10322750 | EPI_ISL_10467196 | EPI_ISL_10561501 | EPI_ISL_10708094 | EPI_ISL_10471386 | EPI_ISL_10771251 | EPI_ISL_17847434 |
| EPI_ISL_10322751 | EPI_ISL_10328523 | EPI_ISL_10326954 | EPI_ISL_10326756 | EPI_ISL_10471743 | EPI_ISL_10325983 | EPI_ISL_10324575 |
| EPI_ISL_10324328 | EPI_ISL_10330826 | EPI_ISL_10327029 | EPI_ISL_10326812 | EPI_ISL_10327037 | EPI_ISL_10326556 | EPI_ISL_10326834 |

- Australia

| EPI_ISL_9986474 | EPI_ISL_10433117 | EPI_ISL_10672742 | EPI_ISL_10774338 | EPI_ISL_11175584 | EPI_ISL_11176434 | EPI_ISL_11629906 |
| --- | --- | --- | --- | --- | --- | --- |
| EPI_ISL_10044928 | EPI_ISL_10433121 | EPI_ISL_10672743 | EPI_ISL_10774341 | EPI_ISL_11175585 | EPI_ISL_11176444 | EPI_ISL_11629907 |
| EPI_ISL_10044933 | EPI_ISL_10433123 | EPI_ISL_10672745 | EPI_ISL_10842365 | EPI_ISL_11175591 | EPI_ISL_11176450 | EPI_ISL_11629921 |
| EPI_ISL_10115969 | EPI_ISL_10433126 | EPI_ISL_10672746 | EPI_ISL_10848589 | EPI_ISL_11175595 | EPI_ISL_11176486 | EPI_ISL_11629977 |
| EPI_ISL_10116915 | EPI_ISL_10433128 | EPI_ISL_10672750 | EPI_ISL_10848590 | EPI_ISL_11175597 | EPI_ISL_11176492 | EPI_ISL_11629978 |
| EPI_ISL_10116963 | EPI_ISL_10433131 | EPI_ISL_10672753 | EPI_ISL_10973435 | EPI_ISL_11175599 | EPI_ISL_11176495 | EPI_ISL_11629982 |
| EPI_ISL_10264992 | EPI_ISL_10433136 | EPI_ISL_10672758 | EPI_ISL_10973437 | EPI_ISL_11175765 | EPI_ISL_11176496 | EPI_ISL_11629983 |
| EPI_ISL_10264995 | EPI_ISL_10433144 | EPI_ISL_10672764 | EPI_ISL_10973438 | EPI_ISL_11175783 | EPI_ISL_11176502 | EPI_ISL_11629984 |
| EPI_ISL_10264996 | EPI_ISL_10433149 | EPI_ISL_10672766 | EPI_ISL_10973439 | EPI_ISL_11175785 | EPI_ISL_11176505 | EPI_ISL_11629985 |
| EPI_ISL_10430134 | EPI_ISL_10433164 | EPI_ISL_10672767 | EPI_ISL_10973440 | EPI_ISL_11175832 | EPI_ISL_11176531 | EPI_ISL_11631379 |
| EPI_ISL_10432895 | EPI_ISL_10433168 | EPI_ISL_10672796 | EPI_ISL_10973441 | EPI_ISL_11175853 | EPI_ISL_11176532 | EPI_ISL_11651964 |
| EPI_ISL_10432896 | EPI_ISL_10433179 | EPI_ISL_10672800 | EPI_ISL_10973451 | EPI_ISL_11175882 | EPI_ISL_11176533 | EPI_ISL_11656688 |
| EPI_ISL_10432897 | EPI_ISL_10433184 | EPI_ISL_10672802 | EPI_ISL_10973452 | EPI_ISL_11175911 | EPI_ISL_11176546 | EPI_ISL_11656689 |
| EPI_ISL_10432899 | EPI_ISL_10433185 | EPI_ISL_10672804 | EPI_ISL_10973454 | EPI_ISL_11175996 | EPI_ISL_11176553 | EPI_ISL_11656690 |
| EPI_ISL_10432900 | EPI_ISL_10433187 | EPI_ISL_10672807 | EPI_ISL_10973457 | EPI_ISL_11176025 | EPI_ISL_11176580 | EPI_ISL_11656692 |
| EPI_ISL_10432969 | EPI_ISL_10433207 | EPI_ISL_10672808 | EPI_ISL_10973835 | EPI_ISL_11176105 | EPI_ISL_11176585 | EPI_ISL_11656693 |
| EPI_ISL_10432970 | EPI_ISL_10433217 | EPI_ISL_10672810 | EPI_ISL_10975182 | EPI_ISL_11176108 | EPI_ISL_11176598 | EPI_ISL_11656694 |
| EPI_ISL_10432971 | EPI_ISL_10433218 | EPI_ISL_10672818 | EPI_ISL_10975199 | EPI_ISL_11176110 | EPI_ISL_11176601 | EPI_ISL_11656696 |
| EPI_ISL_10432974 | EPI_ISL_10433225 | EPI_ISL_10672839 | EPI_ISL_10975307 | EPI_ISL_11176115 | EPI_ISL_11176604 | EPI_ISL_11656697 |
| EPI_ISL_10432975 | EPI_ISL_10433227 | EPI_ISL_10672860 | EPI_ISL_10975330 | EPI_ISL_11176119 | EPI_ISL_11176605 | EPI_ISL_11656698 |
| EPI_ISL_10432976 | EPI_ISL_10433229 | EPI_ISL_10672868 | EPI_ISL_10975383 | EPI_ISL_11176122 | EPI_ISL_11176609 | EPI_ISL_11656699 |
| EPI_ISL_10432977 | EPI_ISL_10433230 | EPI_ISL_10675265 | EPI_ISL_10975517 | EPI_ISL_11176128 | EPI_ISL_11176615 | EPI_ISL_11656700 |
| EPI_ISL_10432978 | EPI_ISL_10433231 | EPI_ISL_10730273 | EPI_ISL_10975529 | EPI_ISL_11176132 | EPI_ISL_11176616 | EPI_ISL_11656701 |
| EPI_ISL_10432979 | EPI_ISL_10433235 | EPI_ISL_10730295 | EPI_ISL_10975553 | EPI_ISL_11176135 | EPI_ISL_11176619 | EPI_ISL_11656702 |
| EPI_ISL_10432980 | EPI_ISL_10433236 | EPI_ISL_10730296 | EPI_ISL_10975713 | EPI_ISL_11176139 | EPI_ISL_11176621 | EPI_ISL_11656703 |
| EPI_ISL_10432981 | EPI_ISL_10433241 | EPI_ISL_10730302 | EPI_ISL_10975716 | EPI_ISL_11176144 | EPI_ISL_11176623 | EPI_ISL_11656704 |
| EPI_ISL_10432982 | EPI_ISL_10433242 | EPI_ISL_10730305 | EPI_ISL_10975718 | EPI_ISL_11176147 | EPI_ISL_11176625 | EPI_ISL_11656705 |
| EPI_ISL_10432983 | EPI_ISL_10433244 | EPI_ISL_10730306 | EPI_ISL_10975805 | EPI_ISL_11176149 | EPI_ISL_11176628 | EPI_ISL_11656706 |
| EPI_ISL_10432985 | EPI_ISL_10433269 | EPI_ISL_10730307 | EPI_ISL_10981507 | EPI_ISL_11176151 | EPI_ISL_11176632 | EPI_ISL_11656707 |
| EPI_ISL_10432986 | EPI_ISL_10433276 | EPI_ISL_10730311 | EPI_ISL_10981588 | EPI_ISL_11176154 | EPI_ISL_11176633 | EPI_ISL_11656708 |
| EPI_ISL_10432989 | EPI_ISL_10433278 | EPI_ISL_10730312 | EPI_ISL_10981589 | EPI_ISL_11176161 | EPI_ISL_11176635 | EPI_ISL_11656709 |
| EPI_ISL_10432991 | EPI_ISL_10433293 | EPI_ISL_10730314 | EPI_ISL_10981590 | EPI_ISL_11176167 | EPI_ISL_11176642 | EPI_ISL_11656711 |
| EPI_ISL_10432993 | EPI_ISL_10433300 | EPI_ISL_10730346 | EPI_ISL_10981616 | EPI_ISL_11176169 | EPI_ISL_11176661 | EPI_ISL_11656712 |
| EPI_ISL_10432994 | EPI_ISL_10433301 | EPI_ISL_10730352 | EPI_ISL_11157187 | EPI_ISL_11176178 | EPI_ISL_11176666 | EPI_ISL_11656713 |
| EPI_ISL_10432996 | EPI_ISL_10433302 | EPI_ISL_10730357 | EPI_ISL_11157194 | EPI_ISL_11176180 | EPI_ISL_11332015 | EPI_ISL_11656714 |
| EPI_ISL_10432999 | EPI_ISL_10433303 | EPI_ISL_10730403 | EPI_ISL_11157203 | EPI_ISL_11176186 | EPI_ISL_11332121 | EPI_ISL_11656715 |
| EPI_ISL_10433000 | EPI_ISL_10433307 | EPI_ISL_10774040 | EPI_ISL_11157205 | EPI_ISL_11176189 | EPI_ISL_11332122 | EPI_ISL_11656716 |
| EPI_ISL_10433001 | EPI_ISL_10433324 | EPI_ISL_10774044 | EPI_ISL_11175365 | EPI_ISL_11176194 | EPI_ISL_11332399 | EPI_ISL_11656717 |
| EPI_ISL_10433002 | EPI_ISL_10433330 | EPI_ISL_10774046 | EPI_ISL_11175366 | EPI_ISL_11176196 | EPI_ISL_11332402 | EPI_ISL_11656718 |
| EPI_ISL_10433004 | EPI_ISL_10433335 | EPI_ISL_10774049 | EPI_ISL_11175367 | EPI_ISL_11176212 | EPI_ISL_11332411 | EPI_ISL_11656721 |
| EPI_ISL_10433006 | EPI_ISL_10433339 | EPI_ISL_10774055 | EPI_ISL_11175368 | EPI_ISL_11176221 | EPI_ISL_11361199 | EPI_ISL_11656722 |
| EPI_ISL_10433010 | EPI_ISL_10433340 | EPI_ISL_10774061 | EPI_ISL_11175369 | EPI_ISL_11176224 | EPI_ISL_11361240 | EPI_ISL_11656723 |
| EPI_ISL_10433011 | EPI_ISL_10433341 | EPI_ISL_10774083 | EPI_ISL_11175370 | EPI_ISL_11176287 | EPI_ISL_11361252 | EPI_ISL_11656724 |
| EPI_ISL_10433012 | EPI_ISL_10433345 | EPI_ISL_10774096 | EPI_ISL_11175371 | EPI_ISL_11176288 | EPI_ISL_11361262 | EPI_ISL_11656725 |
| EPI_ISL_10433013 | EPI_ISL_10433347 | EPI_ISL_10774102 | EPI_ISL_11175372 | EPI_ISL_11176291 | EPI_ISL_11361277 | EPI_ISL_11656727 |
| EPI_ISL_10433014 | EPI_ISL_10433349 | EPI_ISL_10774112 | EPI_ISL_11175373 | EPI_ISL_11176308 | EPI_ISL_11361279 | EPI_ISL_11656728 |
| EPI_ISL_10433015 | EPI_ISL_10433353 | EPI_ISL_10774152 | EPI_ISL_11175380 | EPI_ISL_11176309 | EPI_ISL_11361282 | EPI_ISL_11656730 |
| EPI_ISL_10433016 | EPI_ISL_10433354 | EPI_ISL_10774153 | EPI_ISL_11175401 | EPI_ISL_11176311 | EPI_ISL_11361285 | EPI_ISL_11656731 |
| EPI_ISL_10433017 | EPI_ISL_10564489 | EPI_ISL_10774155 | EPI_ISL_11175402 | EPI_ISL_11176312 | EPI_ISL_11361299 | EPI_ISL_11656732 |
| EPI_ISL_10433018 | EPI_ISL_10564497 | EPI_ISL_10774157 | EPI_ISL_11175404 | EPI_ISL_11176314 | EPI_ISL_11361302 | EPI_ISL_11656733 |
| EPI_ISL_10433019 | EPI_ISL_10564881 | EPI_ISL_10774167 | EPI_ISL_11175405 | EPI_ISL_11176320 | EPI_ISL_11361415 | EPI_ISL_11656734 |
| EPI_ISL_10433020 | EPI_ISL_10565515 | EPI_ISL_10774171 | EPI_ISL_11175406 | EPI_ISL_11176324 | EPI_ISL_11361422 | EPI_ISL_11656735 |
| EPI_ISL_10433021 | EPI_ISL_10565522 | EPI_ISL_10774175 | EPI_ISL_11175407 | EPI_ISL_11176328 | EPI_ISL_11361428 | EPI_ISL_11656736 |
| EPI_ISL_10433022 | EPI_ISL_10565538 | EPI_ISL_10774193 | EPI_ISL_11175408 | EPI_ISL_11176329 | EPI_ISL_11361536 | EPI_ISL_11656737 |
| EPI_ISL_10433023 | EPI_ISL_10565544 | EPI_ISL_10774223 | EPI_ISL_11175409 | EPI_ISL_11176332 | EPI_ISL_11361537 | EPI_ISL_11656738 |
| EPI_ISL_10433028 | EPI_ISL_10565545 | EPI_ISL_10774231 | EPI_ISL_11175410 | EPI_ISL_11176335 | EPI_ISL_11361545 | EPI_ISL_11656740 |
| EPI_ISL_10433030 | EPI_ISL_10565546 | EPI_ISL_10774234 | EPI_ISL_11175411 | EPI_ISL_11176336 | EPI_ISL_11361590 | EPI_ISL_11656741 |
| EPI_ISL_10433031 | EPI_ISL_10565550 | EPI_ISL_10774236 | EPI_ISL_11175412 | EPI_ISL_11176337 | EPI_ISL_11361591 | EPI_ISL_11656742 |
| EPI_ISL_10433035 | EPI_ISL_10565717 | EPI_ISL_10774241 | EPI_ISL_11175413 | EPI_ISL_11176347 | EPI_ISL_11361592 | EPI_ISL_11657316 |
| EPI_ISL_10433036 | EPI_ISL_10565828 | EPI_ISL_10774244 | EPI_ISL_11175421 | EPI_ISL_11176351 | EPI_ISL_11361593 | EPI_ISL_11657327 |
| EPI_ISL_10433037 | EPI_ISL_10565855 | EPI_ISL_10774246 | EPI_ISL_11175424 | EPI_ISL_11176358 | EPI_ISL_11361594 | EPI_ISL_11657334 |
| EPI_ISL_10433038 | EPI_ISL_10565877 | EPI_ISL_10774251 | EPI_ISL_11175426 | EPI_ISL_11176360 | EPI_ISL_11361595 | EPI_ISL_11657338 |
| EPI_ISL_10433039 | EPI_ISL_10565879 | EPI_ISL_10774252 | EPI_ISL_11175428 | EPI_ISL_11176364 | EPI_ISL_11361613 | EPI_ISL_11657344 |
| EPI_ISL_10433041 | EPI_ISL_10565886 | EPI_ISL_10774256 | EPI_ISL_11175429 | EPI_ISL_11176369 | EPI_ISL_11361614 | EPI_ISL_11657365 |
| EPI_ISL_10433046 | EPI_ISL_10565894 | EPI_ISL_10774264 | EPI_ISL_11175430 | EPI_ISL_11176375 | EPI_ISL_11361615 | EPI_ISL_11657401 |
| EPI_ISL_10433067 | EPI_ISL_10565895 | EPI_ISL_10774269 | EPI_ISL_11175432 | EPI_ISL_11176376 | EPI_ISL_11361616 | EPI_ISL_11657408 |
| EPI_ISL_10433070 | EPI_ISL_10566123 | EPI_ISL_10774277 | EPI_ISL_11175435 | EPI_ISL_11176378 | EPI_ISL_11361890 | EPI_ISL_11657409 |
| EPI_ISL_10433083 | EPI_ISL_10566200 | EPI_ISL_10774287 | EPI_ISL_11175437 | EPI_ISL_11176415 | EPI_ISL_11384008 | EPI_ISL_11657410 |
| EPI_ISL_10433084 | EPI_ISL_10672679 | EPI_ISL_10774299 | EPI_ISL_11175440 | EPI_ISL_11176416 | EPI_ISL_11629889 | EPI_ISL_11657435 |
| EPI_ISL_10433085 | EPI_ISL_10672726 | EPI_ISL_10774300 | EPI_ISL_11175449 | EPI_ISL_11176417 | EPI_ISL_11629893 | EPI_ISL_11657455 |
| EPI_ISL_10433089 | EPI_ISL_10672730 | EPI_ISL_10774326 | EPI_ISL_11175459 | EPI_ISL_11176427 | EPI_ISL_11629894 | EPI_ISL_11657456 |
| EPI_ISL_10433099 | EPI_ISL_10672732 | EPI_ISL_10774327 | EPI_ISL_11175522 | EPI_ISL_11176433 | EPI_ISL_11629895 | EPI_ISL_11657484 |
| EPI_ISL_12678877 | EPI_ISL_12275852 | EPI_ISL_12032615 | EPI_ISL_11686057 | EPI_ISL_11657565 | EPI_ISL_11657518 | EPI_ISL_11657487 |
| EPI_ISL_12846301 | EPI_ISL_12275854 | EPI_ISL_12039729 | EPI_ISL_11834234 | EPI_ISL_11686052 | EPI_ISL_11657551 | EPI_ISL_11657513 |
