# Supplementary material for: Genetic features of SARS-CoV-2 Alpha, Delta, and Omicron variants and their association with the clinical severity of COVID-19 in Vietnam
Source: IJID Reg. 2024 Mar 13;11:100348. doi: 10.1016/j.ijregi.2024.03.003 (PMC11004080; doi:10.1016/j.ijregi.2024.03.003)
Supplement: Supplementary file 2 [file mmc2.docx]

**Supplement table**

Supplement table 1

Correlation between age group and comorbidities in patients with alpha, delta, and omicron variants

| **Characteristics** | B.1.1.7  (n = 48) | B.1.617.2  (n = 42) | AY.57  (n = 54) | BA.1.1  (n = 9) | BA.2  (n = 87) | **p** |
| --- | --- | --- | --- | --- | --- | --- |
| Age group (years), n (%) | |  |  |  |  |  |
| <18 | 2 (4.2) | 6 (14.3) | 4 (7.4) | 1 (11.1) | 10 (11.5) | >0.05 |
| 18-40 | 22 (45.8) | 16 (38.1) | 22 (40.7) | 4 (44.4) | 29 (33.3) |  |
| 40-60 | 15 (31.2) | 10 (23.8) | 16 (29.6) | 2 (22.2) | 28 (32.2) |  |
| >60 | 9 (18.8) | 10 (23.8) | 12 (22.3) | 2 (22.2) | 20 (23.0) |  |
| Mean ± SD | 43.56 ± 17.84 | 43.93 ± 24.83 | 50.17 ± 25.42 | 50.33 ± 27.29 | 43.52 ± 24.77 |  |
| **Comorbidities, n (%)** | |  |  |  |  |  |
| Diabetes | 5 (10.4) | 5 (11.9) | 7 (12.9) | 1 (11.1) | 10 (11.5) | >0.05 |
| Cardiovascular | 12 (25.0) | 10 (23.8) | 12 (22.2) | 2 (22.2) | 21 (24.1) |  |
| Cancer | 1 (2.1) | 3 (7.1) | 3 (5.6) | 0 (0.0) | 5 (5.7) |  |
| Pneumonia | 2 (4.2) | 2 (4.8) | 4 (7.4) | 1 (11.1) | 6 (6.9) |  |
| Stroke | 1 (2.1) | 2 (4.8) | 3 (5.6) | 0 (0.0) | 5 (5.7) |  |
| Obesity | 2 (4.2) | 4 (9.5) | 3 (5.6) | 1 (11.1) | 6 (6.9) |  |
| Kidney disease | 0 (0.0) | 3 (7.1) | 4 (7.4) | 0 (0.0) | 7 (8.0) |  |

Abbreviation: SD, standard deviation.

**Supplement table 2**

Regression analysis for variables affecting the mortality of COVID-19

|  | | Characteristics  n (%) or mean (SD) | | Regression model | | | |
| --- | --- | --- | --- | --- | --- | --- | --- |
|  |  | Deaths | Survivors | Univariate | *P* | Multivariate | *P* |
|  |  |  |  | OR (95% CI) |  | OR  (95% CI) |  |
| Age | | 68.71 ± 18.73 | 42.89 ± 23.15 | 1.06  (1.03-1.08) | <0.001 | 1.05  (1.03-1.08) | <0.001^*^ |
| Gender | Female | 11 (35.5) | 113 (54.1) | 2.14  (0.98-4.69) | >0.05 | 2.53  (0.95-6.75) | >0.05^*^ |
|  | Male | 20 (64.5) | 96 (45.9) |  |  |  |  |
| Comorbidities | Yes | 25 (80.6) | 89 (42.6) | 5.62  (2.21-14.27) | <0.001 | 2.63  (0.87-7.94) | >0.05^*^ |
|  | No | 06 (19.4) | 120 (57.4) |  |  |  |  |
| Variants | Delta | 27 (87.1) | 69 (33.0) | 13.70  (4.61-40.69) | <0.001 | 12.49  (3.95-39.51) | <0.001^*^ |
|  | Others | 04 (12.9) | 140 (67.0) |  |  |  |  |
| Vaccination  (≥2 dose) | Yes | 09 (29.0) | 62 (29.7) | 0.97  (0.42-2.23) | >0.05 | 0.77  (0.27-2.15) | >0.05^*^ |
|  | No | 22 (71.0) | 147 (70.3) |  |  |  |  |

Abbreviations: COVID-19, coronavirus disease 2019; SD, standard deviation.

**Supplement table 3**

The comparison of the disease levels, vaccination status, and treatment outcome of this study with other studies

|  | | **This study** | **Frank P. Esper [1]** | **Radhakrishnan [2]** | **Skarbinski [3]** | **Bahl [4]** |
| --- | --- | --- | --- | --- | --- | --- |
| Alpha | Mild | 15 (31.2) | - | - | - | - |
|  | Moderate | 28 (58.3) | - | - | - | - |
|  | Severe | 05 (10.5) | - | - | - | - |
| Delta | Mild | 24 (25.0) | - | 45 (18.7) | - | - |
|  | Moderate | 13 (13.5) | - | 46 (19.1) | - | - |
|  | Severe | 59 (61.5) | - | 150 (62.3) | - | - |
| Omicron | Mild | 54 (56.2) | - | 131 (36.0) | - | - |
|  | Moderate | 20 (20.8) | - | 74 (20.3) | - | - |
|  | Severe | 22 (23.0) | - | 143 (39.3) | - | - |
| Alpha | Survive | 48 (100.0) | 1138(98.7) | - | - | 135(98.55) |
|  | Death | 00 (0.0) | 15 (1.3) | - | - | 2 (1.45) |
| Delta | Survive | 69 (71.9) | 800(99.0) | 240(95.0) | 67,807(99.3) | 251(100) |
|  | Death | 27 (28.1) | 8 (1.0) | 12 (5.0) | 478 (0.7) | 0 (0.0) |
| Omicron | Survive | 92 (95.8) | 693 (99.4) | 340(92.9) | (99.8) | 250(99.6) |
|  | Death | 04 (4.2) | 3 (0.4) | 26 (7.1) | 83 (0.2) | 1 (0.4) |
| Alpha | Not vaccine | 48 (100.0) | 1119(96.8) | - | - | 132(95.65) |
|  | Full vaccine | 00 (0.0) | 37 (3.2) | - | - | 6 (4.35) |
| Delta | Not vaccine | 68 (80.8) | 592(73.1) | 231(98.3) | 39,055 (56) | 171(95.53) |
|  | Full vaccine | 28 (19.2) | 218 (26.9) | 4 (1.7) | 30,686(44) | 8 (4.47) |
| Omicron | Not vaccine | 53 (55.2) | 186(26.7) | 325(89.3) | 10,635 (22) | 228(91.57) |
|  | Full vaccine | 43 (44.8) | 513 (73.3) | 39 (10.7) | 37,705(78) | 21 (8.43) |

*Note:*

[1] Esper FP, Adhikari TM, Tu ZJ, Cheng YW, El-Haddad K, Farkas DH, et al. Alpha to Omicron: Disease Severity and Clinical Outcomes of Major SARS-CoV-2 Variants. J Infect Dis. 2023;227:344-52.

[2] Radhakrishnan N, Liu M, Idowu B, Bansari A, Rathi K, Magar S, et al. Comparison of the clinical characteristics of SARS-CoV-2 Delta (B.1.617.2) and Omicron (B.1.1.529) infected patients from a single hospitalist service. BMC Infect Dis. 2023;23:747.

[3] Skarbinski J, Wood MS, Chervo TC, Schapiro JM, Elkin EP, Valice E, et al. Risk of severe clinical outcomes among persons with SARS-CoV-2 infection with differing levels of vaccination during widespread Omicron (B.1.1.529) and Delta (B.1.617.2) variant circulation in Northern California: A retrospective cohort study. Lancet Reg Health Am. 2022;12:100297.

[4] Bahl A, Mielke N, Johnson S, Desai A, Qu L. Severe COVID-19 outcomes in pediatrics: An observational cohort analysis comparing Alpha, Delta, and Omicron variants. Lancet Reg Health Am. 2023;18:100405.
